# Reversible mechanofluorochromism of aniline-terminated phenylene ethynylenes†
†Electronic supplementary information (ESI) available: Experimental methods, synthetic methods, NMR spectra, HRMS, photophysical data, DSC data, PXRD data, crystallographic tables. CCDC 1826630–1826633. For ESI and crystallographic data in CIF or other electronic format see DOI: 10.1039/c8sc00980e


**DOI:** 10.1039/c8sc00980e

**Published:** 2018-05-24

**Authors:** Seth A. Sharber, Kuo-Chih Shih, Arielle Mann, Fanny Frausto, Terry E. Haas, Mu-Ping Nieh, Samuel W. Thomas

**Affiliations:** a Department of Chemistry , Tufts University , 62 Talbot Avenue , Medford , MA 02155 , USA . Email: sam.thomas@tufts.edu; b Department of Chemical & Biomolecular Engineering , University of Connecticut , 97 North Eagleville Road, Storrs , CT 06269 , USA

## Abstract

Seven three-ring phenylene-ethynylene (PE) structural analogs, differing only in the lengths of alkyl chains on terminal aniline substituents, show 50–62 nm bathochromic shifts in emission maxima in response to mechanical force (mechanofluorochromism, MC). These shifts are fully reversible with heat or solvent fuming. Shearing of these solids yields a transition from green-emitting crystalline phases to orange-emitting amorphous phases as established by differential scanning calorimetry and X-ray diffraction. Molecules with shorter alkyl chain lengths required higher temperatures to recover the hypsochromically shifted crystalline phases after grinding, while the recovery with chain lengths longer than butyl occurred at room temperature. In addition to this structure-dependent thermochromism, these compounds retain their MC properties in polymer hosts to various extents. The crystalline phases of these materials have PE chromophores that are twisted due to non-covalent perfluoroarene–arene (ArF–ArH) interactions involving perfluorophenyl pendants and the terminal rings of the PE chromophore, resulting in interrupted conjugation and an absence of chromophore aggregation. The MC behavior of an analog without the perfluoroarene rings is severely attenuated. This work demonstrates the general utility of twisted PEs as stimuli-responsive moieties and reveals clear structure–property relationships regarding the effects of alkyl chain length on these materials.

## Abstract 2

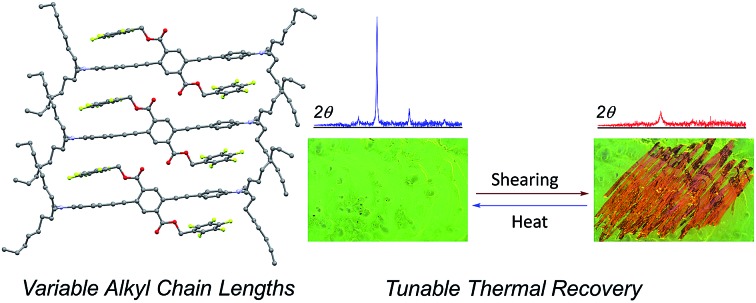
Alkyl chain length tunes the reversion temperature of mechanofluorochromic phenylene-ethynylenes that show reversible force-induced change of fluorescence from green to orange.

## Introduction

This paper describes a series of dialkylaniline-based PEs that display reversible mechanofluorochromism with alkyl chain length dependent properties. Mechanofluorochromic (MC) materials, which show changes in the intensity and/or wavelengths of luminescence in response to mechanical force, have experienced extensive development in the last decade.^1^ This response to mechanical force is often reversible with application of light, heat, or exposure to solvent vapor (fuming). MC materials have shown promise for applications in pressure sensing,^2^,^3^ including in biologically relevant applications and wearable technology.^4^–^6^ Other applications of MC materials have centered on molecular electronics, optical data storage, secure encoding of information, and logic gates.^7^–^11^ Development of MC responsive solids beyond small molecule assemblies into polymers^12^ and functional materials is currently expanding the utility of these materials.

While mechanisms of MC transitions are diverse, the general phenomenon depends upon molecular assemblies converting between sufficiently dissimilar states, each with differing conformations and/or intermolecular interactions that yield unique optical properties. Including competitive non-covalent interactions that stabilize differing states,^13^ such as weak aromatic interactions that prefer dissimilar crystal phases,^14^ or hydrogen-bonded assemblies that can be disrupted with mechanical force,^15^ is a common strategy for designing MC behavior. Structure–property relationships often provide mechanistic insight for MC materials; these studies include how chromophore structure impacts the force-induced changes in luminescence efficiency, the magnitude and direction of spectral shift,^14^,^16^–^24^ as well as the sensitivity to mechanical force.^25^–^27^ Though alkyl side chains are not traditionally considered key to the optoelectronics of conjugated materials, the concept of side chain engineering has become increasingly popular for the optimization of optoelectronic devices in recent years.^28^,^29^ In addition, their structures can influence MC behavior^30^–^32^ through perturbations of the differences in energies between polymorphs.^33^–^35^ In some thermally reversible MC materials, the lengths of alkyl substituents can tune the cold-crystallization temperature (*T*_cc_, or the heat-recovery temperature) for reversion from the force-induced metastable state; an example of this trend has been observed in cruciform divinylanthracenes with alkyl substituents.^36^,^37^ The ability to tune sensitivity to external stimulus through straightforward structural modification is valuable for practical applications of MC molecular assemblies, as shown in examples of thermochromic devices.^38^–^40^

**Fig. 1:**
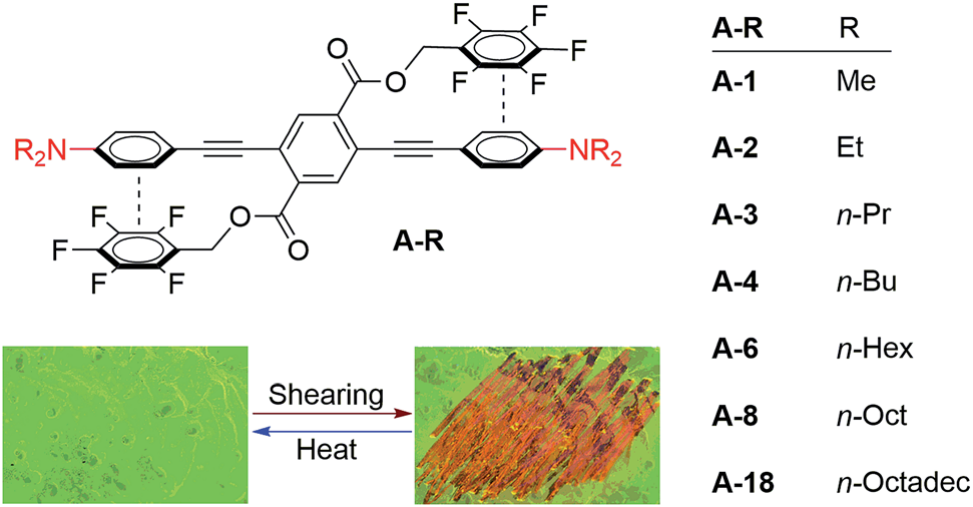
Chemical structure and example mechanofluorochromic response of aniline terminated three-ring phenylene-ethynylenes.

As a broad category of chromophores, the optoelectronic properties of PEs can be highly sensitive to perturbation, making them attractive candidates for stimuli-responsive materials. PEs generally have small energy barriers for rotation, while the extent of coplanarity of rings along PEs has a large influence on the energies of molecular orbitals and radiative transitions.^41^,^42^ There have been numerous creative examples of controlling the coplanarity (or lack thereof) of PEs, and thereby their optical properties, through covalent bonding or non-covalent interactions.^43^–^54^ Moreover, the propensity of solid PEs to aggregate and show bathochromic shifts and quenched luminescence presents additional opportunities and challenges for rational design of chemical structure to optimize PE-based solid-state fluorophores.^44^,^51^,^55^–^63^ Our group has reported a class of three-ring PEs in which fluorinated aromatic side chains interact cofacially with non-fluorinated terminal rings of the PE chromophore (ArF–ArH interactions), resulting in large inter-ring torsional angles of 63–88° (Fig. 1) along the PE backbone.^64^,^65^ This structural motif both interrupts conjugation along the PE and prevents intermolecular aggregation, yielding solids that have absorbance and fluorescence spectra either hypsochromically shifted from or similar to those in dilute solution. In contrast, solids of compounds lacking these side chain/main chain ArF–ArH interactions have planarized and/or aggregated PE chromophores, with absorbance and fluorescence spectra bathochromically shifted from solution. Our work has also enabled rational design of these solid-state properties: tuning the energy of the ArF–ArH interaction through the electronic effects of substituents, as well as integrating other competing non-covalent interactions such as hydrogen bonds, can dictate the solid-state packing structure and the consequent optical properties. This sensitivity of the balance of non-covalent interactions, molecular packing, and optical properties to small structural changes can also render these solids responsive to mechanical force. As an example, we reported the MC behavior of one such compound, an octyloxy-substituted PE with absorbance and fluorescence that shifted bathochromically (from blue to green emissive) upon application of mechanical force.^64^

Herein we describe a series of seven 3-ring PE analogs, each with different lengths of alkyl chains on the nitrogen atoms of terminal aniline rings. These compounds show reversible, force-induced bathochromic shifting from green to orange emission. The objectives of this work are the following: (i) develop a series of PE-based MC materials with emission spectra red-shifted relative to those we reported previously, (ii) determine how the length of the alkyl substituents impact the force-responsive spectroscopy and thermal reversibility of the MC transitions, (iii) clarify the nature of the polymorphs accessed before and after MC transition of these materials, and (iv) determine the extent to which polymer films can be viable hosts for these materials while retaining MC activity. These results are important for the development of new MC compounds that respond to different temperatures, improving our understanding of MC luminogens, and extending the viability of MC function into polymeric materials.

## Results and discussion

**Scheme 1:**
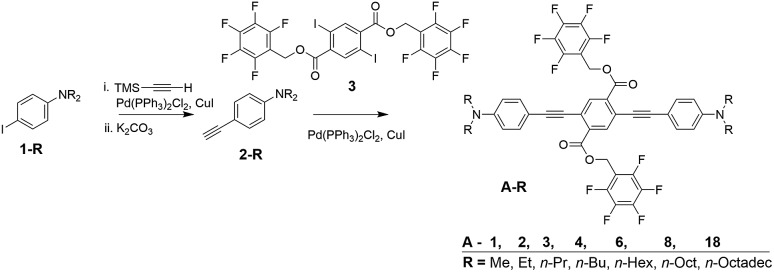
Syntheses of aniline-substituted PEs **A-R** that vary in alkyl chain lengths.

Scheme 1 shows the series of seven molecules that we prepared and studied in this work. These three-ring PEs have the same general structural features: a central perfluorobenzyl terephthalate ring flanked by substituted arylacetylenes. We chose *p*-dialkylaniline terminal rings for this study because the strong electron donating effect of the amine substituent is expected to yield particularly strong cofacial stacking interactions with the perfluorobenzene side chains, which dictate the twisted conformation of PEs from the perspective of electrostatic considerations. The correlation of traditional Hammett-type substituent effects with ArF–ArH interaction strength have been established in both experimental and theoretical studies comprising monosubstituted ArH rings.^65^–^69^ The series of bis(dialkylaniline) PEs discussed here vary in the lengths of the *N*-alkyl chains, ranging from as short as methyl to as long as *n*-octadecyl.

The syntheses of these molecules, **A-R**, in which “**R**” designates the length of the alkyl substituents, was straightforward (Scheme 1). Dialkylation of 4-iodoaniline under basic conditions yielded **1-R**, except for **1-1**, which is commercially available. Sonogashira coupling with trimethylsilylacetylene and subsequent deprotection gave the terminal arylacetylenes **2-R**. A final double Sonogashira coupling between each of **2-R** and the previously described 2,5-diiodoterephthalate **3**^64^ yielded the target compounds **A-R**, each of which were purified by recrystallization.^64^,^65^ A control compound bearing non-fluorinated benzyl ester pendants and therefore lacking the ArF–ArH interaction, **A-1F0**, was analogously synthesized from non-fluorinated 2,5-diiodoterephthalate precursor **3-F0**.^29^

### Photophysical properties in solution and solids

**Fig. 2:**
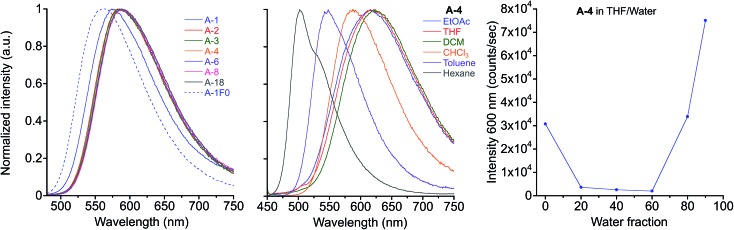
Emission of **A-R** compounds in chloroform solution, *λ*_ex_ = 450 nm (left) and emission of **A-4** in various solvents, *λ*_ex_ = 440 nm (center). Fluorescence intensity at 600 nm of **A-4** in THF/water mixtures (right).

Photophysical properties of the target compounds in solution are similar throughout the series of compounds (Table 1 & Fig. 2). Absorbance and emission maxima of yellow to orange emitting chloroform solutions show lower energy optical spectra with alkyl chain lengths longer than methyl due to increased donor character of the terminal aniline substituents (Fig. S1†). All compounds display positive solvatochromism, which is consistent with the donor–acceptor nature of these PE chromophores, with emission maxima varying up to 122 nm from hexane (*λ*_max_ = 502 nm for **A-4**) to dichloromethane (DCM) solutions (*λ*_max_ = 624 nm for **A-4**). Fluorescence is noticeably quenched in higher polarity solvents: for **A-4** we observe a quantum yield of 0.72 and lifetime of 2.7 ns in hexane, which decrease to 0.23 and 1.7 ns in chloroform, and drop sharply to 0.03 and 0.4 ns in orange emitting DCM solution. This quenching increases with *E*_T_(30) values of the solvents, and indicates that non-radiative decay pathways become dominant in polar solvents (Fig. S2†). While *k*_r_ drops four-fold between hexane and ethyl acetate, *k*_nr_ increases over thirty-fold. This coincidence of decreasing *k*_r_ with increasing *E*_T_(30) values is consistent with the “positive solvatokinetic effect,”^70^ of donor–acceptor type compounds, and demonstrates intramolecular charge transfer (ICT) in the excited state, in which an increase in dipole moment of the excited state relative to the ground state with solvent polarity enhances non-radiative decay.^71^–^76^ Further, while quenched in pure tetrahydrofuran (THF) solution (*Φ*_F_ = 0.03 for **A-4**, 0.04 for **A-18**), these materials show aggregation-induced enhanced emission (AIEE)^77^–^80^ in THF/water mixtures, which often occurs in force responsive materials (Fig. 2).^13^,^20^,^81^ While the emission intensity varies among the compounds, a representative sample of the series (**A-2**, **A-4**, **A-8**, **A-18**) consistently shows turn-on fluorescence at or above 60% water fraction (*f*_w_) with roughly a three-fold increase of intensity relative to pure THF solution and gradual hypsochromic shift in emission spectra with increasing water fraction (38 nm shift in *λ*_max_ from 0% to 90%, Fig. S3†). We attribute the observed AIEE effect to a combination of reduction in the local polarity of nanoprecipitated fluorophores and the prevention of cofacial interactions between PE chromophore units by ArF–ArH interactions (*vide infra*).

**Table 1:** Summary of optical spectra of all compounds in dilute chloroform solution

	Abs. *λ*_max_ (nm)	Em. *λ*_max_ (nm)	*Φ*_F_	*τ* (ns)
**A-1**	436	575	0.21	1.6
**A-2**	448	585	0.19	1.7
**A-3**	450	585	0.20	1.6
**A-4**	450	588	0.23	1.7
**A-6**	452	590	0.19	1.7
**A-8**	455	590	0.20	1.7
**A-18**	453	588	0.23	1.7
**A-1F0**	432	562	0.41	2.7

As powders or drop-cast films, compounds **A-R** are yellow with green fluorescence, with absorbance maxima ranging from 400–424 nm and emission maxima from 510–550 nm after heating to 100–220 °C to remove solvent and allow relaxation toward a more thermodynamically stable phase. This initial annealing has only modest effects on the absorbance and fluorescence of drop-cast films, which have uniform green emission. The rapidly deposited films resulting from spin casting, however, are green emissive, yellow/orange emissive, or both. Subsequent thermal annealing hypsochromically shifts this emission to uniform green color, similar to those observed in drop-cast films. While emission maxima blue-shift somewhat with alkyl chains longer than methyl and ethyl (up to 40 nm shift between **A-1** and **A-18**), minor changes in the position of thin film absorbance and emission spectra between compounds do not correlate with increasing alkyl chain length.

The luminescence of these films is most akin to compounds dissolved in toluene, and the spectra are hypsochromically shifted relative to chloroform solutions by 15–50 nm in absorbance *λ*_max_ and 25–50 nm in emission *λ*_max_. As determined in our previous work, this lack of a significant red-shift for this class of PE solids indicates solid-state packing environments in which the conjugated chromophores lack two features common in other PE-based materials: (i) highly coplanar aryl rings along the PE backbones and (ii) intermolecular aggregation of PE chromophores. The combination of intramolecular and intermolecular ArF–ArH interactions between the perfluorobenzyl pendants on the central terephthalate ring and the terminal rings of the PE backbone both enforce these twisted conformations and prevent aggregation between the PE chromophores. For reference, compounds we reported previously that show the twisted, non-aggregated arrangement in crystal structures showed modest solution-to-solid hypsochromic shifts relative to chloroform solutions either in absorbance and/or emission (15–25 nm in absorbance *λ*_max_ and 5–20 nm in emission *λ*_max_). In contrast, analogs that showed coplanar and/or aggregated PE chromophores in their crystal structures showed significant bathochromic shifts as solids (14–42 nm in absorbance *λ*_max_ and 60–128 nm in emission *λ*_max_).^65^ Only **A-1F0**, which lacks fluorinated pendants, demonstrates this bathochromic shift in recrystallized and annealed solids, and never shows the hypsochromically shifted green emission.

### Crystallography

**Fig. 3:**
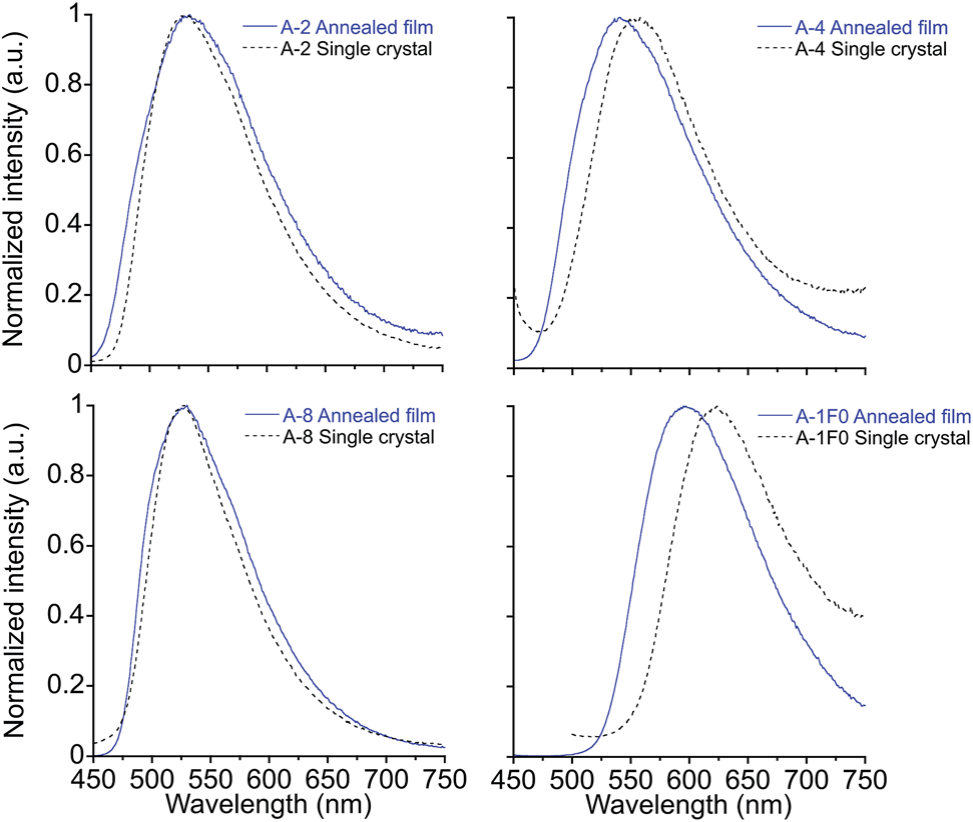
Emission spectra of annealed thin films and single crystals for **A-2**, **A-4**, **A-8** and **A-1F0**. *λ*_ex_ = 430 nm for **A-2**, **A-4**, and **A-1F0**. *λ*_ex_ = 420 nm for **A-8**.

Crystal structures obtained for compounds **A-2**, **A-4**, **A-8**, and **A-1F0** and provide key insight for the stimuli-responsive behavior of this series of molecules. As with the annealed films, single crystals of these compounds appeared as yellow needles with green fluorescence. Emission spectra of the single crystals resemble those of the films, as seen in Fig. 3 with only modest shifts between film and crystal emission *λ*_max_ (533 *vs.* 530 nm in **A-2**, 535 *vs.* 556 nm in **A-4**, 530 *vs.* 526 nm in **A-8**, and 597 *vs.* 625 nm in **A-1F0**). Similar correlations between excitation spectra and onset wavelengths exist.

The green emitting crystals of **A-2**, **A-4**, and **A-8** have twisted crystal structures (Fig. 5) similar to the previously reported **A-1**, with ArF–ArH cofacial interactions as the central features. These interactions occur both intramolecularly and intermolecularly, resulting in ArF–ArH stacks that propagate along infinite columns with centroid–centroid distances of 3.6–4.0 Å. These ArF–ArH interactions, combined with the tendency of the ester functional groups to remain coplanar with the central phenylene ring, result in heavily twisted PE backbones. The inter-ring torsional angles along the PE chromophores are between 63.5° and 88.2°. The alkyl chains in structures of **A-2**, **A-4**, and **A-8** extend in a roughly perpendicular trajectory from planes defined by the aniline rings of the PE backbones, which likely maintains close packing between neighboring stacks of the twisted PEs, as opposed to the possibility of alkyl chains extending parallel to the aniline rings, which may disrupt close packing.

**Fig. 4:**
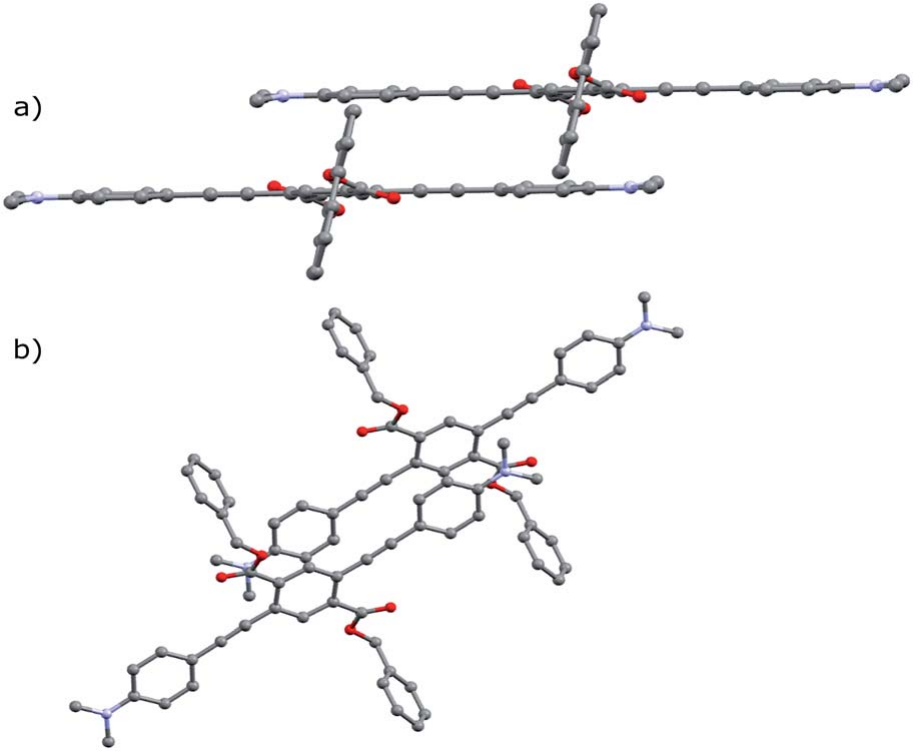
Crystal structure of **A-1F0** viewed along (a) coplanar PE backbone (b) *b*-axis of unit cell. Hydrogen atoms omitted for clarity.

**Fig. 5:**
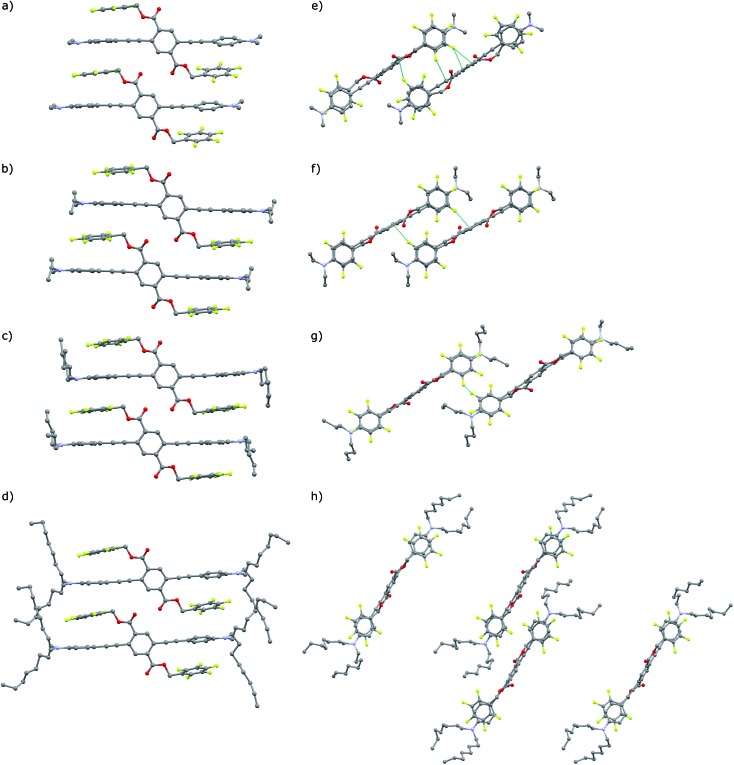
(a–d) Crystal structures of **A-1**, **A-2**, **A-4**, **A-8**, with highlighted close contacts between molecules in neighboring columns dominated by intra and intermolecular ArF–ArH stacked arenes. (e) and (f) View down the *a*-axis of **A-1** and **A-2**. (g) Perspective is replicated in **A-4**. (h) Unit cell of **A-8** viewed down the *b*-axis. Hydrogen atoms and disordered carbon atoms in alkyl chains (**A-4**, **A-8**) omitted for clarity.

Notably, the number of intermolecular aromatic interactions between neighboring PEs decreases with alkyl chain length. The structure for **A-1** shows C–H···π and C–F···π interactions between neighboring ArF rings and terminal anilines to terephthalates (2.9 Å closest contacts). These interactions are mostly conserved in the structure for **A-2**, though the number of close contacts between stacks is reduced (3.0 Å closest contacts). Packing in **A-4** changes significantly; these C–H···π and C–F···π interactions are lost as neighboring PEs show a 7 Å pitch displacement relative to **A-1** and **A-2** (Fig. 5 and S4†). As the alkyl chains occupy a greater volume in the structure, the ArF rings and anilines are farther away from neighboring terephthalates. Only F···F interactions are present at 2.9 Å in **A-4**. A dramatic change is seen in **A-8**, where there are no close contacts between neighboring chromophores, and a greater portion of the volume of the unit cell is occupied only by the octyl chains. This inhibition of close packing is accompanied by significant disorder in the alkyl chains of **A-4** and **A-8** (whole molecule disorder in **A-4**), coinciding with significant difficulty in obtaining single crystals of these compounds. These differences in crystal packing have important consequences for the variation of MC properties (*vide infra*). The correlation of optical spectra and crystal structures in this series of compounds support all aniline-terminated 3-ring PEs **A1–A18** having twisted PE backbones without aggregation. This relative similarity in fluorescence in all cases where crystal structures could be obtained supports the conclusion that molecular packing in thin films favors the twisted, non-aggregated motif seen in crystal structures. Powder X-ray diffraction (PXRD) studies of drop-cast films also supports this conclusion. In contrast, an orange emitting crystal of **A-1F0** (Fig. 4) shows aggregated PE chromophores with coplanar arenes.

### Reversible mechanofluorochromism

**Fig. 6:**
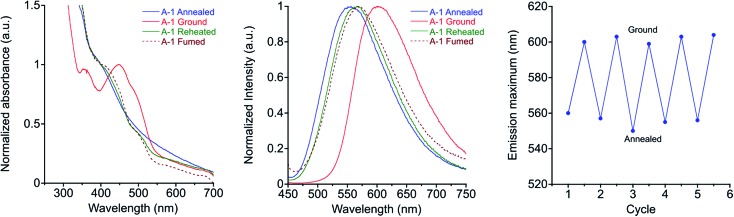
**A-1** characteristic reversible MC behaviour of films drop-cast from chloroform: absorbance (left), emission (center) spectra after annealing, grinding, second heating, and fuming with solvent vapor, *λ*_ex_ = 430 nm. Plot of emission maxima over five heat–grinding cycles (right).

These seven compounds all show easily noticeable bathochromic shifts in both color (yellow to orange, 37–50 nm shift in absorbance *λ*_max_) and fluorescence (green to orange, 50–62 nm shift in emission *λ*_max_) in response to mechanical force (Fig. 6 and S5, S6†). This bathochromic shift is consistent with planarization/aggregation of at least some of the PE chromophores.^56^,^57^ Though this transition can be achieved to some degree with hydrostatic pressure (15 000 psi), powders and films are far more sensitive to shear force such as rubbing with a spatula. The extent of the bathochromic shift in optical spectra is similar across the compounds with modest differences primarily observed in the emission of the annealed films. This response is fully reversible upon thermal annealing or with solvent vapor annealing and does not fatigue over multiple cycles of heating and grinding for any of the target compounds (Fig. 6 and S7, S8†). The films become increasingly waxy with decreased melting points with longer alkyl chains (282 °C for **A-1** to 76 °C for **A-18**), and are more easily deformed by rubbing.

The persistence of metastable orange emission from ground films varies greatly across the series. For compounds with alkyl chains longer than propyl, the orange emission of ground films relaxes to yellow at room temperature within minutes. After grinding, the original fluorescence of **A-4** mostly recovers at room temperature within 15 minutes. The ground phase becomes increasingly transient for **A-6** and **A-8** where the orange color may be seen on the timescale of 1 s, and is not observable whatsoever for **A-18** at room temperature, but only momentarily *ca.* –80 °C by cooling over a dry ice/acetone bath. Therefore, the recorded emission maxima for sheared films of **A-6** and **A-8** do not reach *ca.* 600 nm, as do those of **A-1** through **A-4**, instead showing smaller bathochromic shifts to 550 nm. An interesting feature of this transient response is that films of **A-8** recover their green emission immediately after grinding, and may be sheared continuously with no loss of MC behavior, with a single film maintaining its activity over the course of at least a year.

**Fig. 7:**
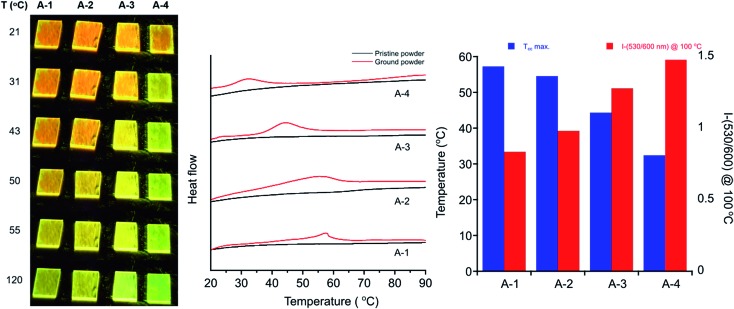
(Left) Images of fluorescent drop-cast films of **A-1** through **A-4** after grinding while illuminating with a hand-held UV lamp. Films were heated at 5 °C min^–1^ and photographs taken for each progressive initialization of recovery toward green emission. (Center) DSC traces of ground powders (red) and pristine powders (black) (exo up). Ground powder curves rotated for clarity. (Right) Graph of *T*_cc_ maximum for **A-R** plotted with emission intensity ratio of 530/600 nm after heating ground powders at 100 °C for 15 minutes.

Differential scanning calorimetry (DSC) of these molecules also demonstrates the effect of alkyl chain length on recovery from the metastable polymorph created upon grinding. After thermal annealing, no transitions are visible for these compounds between ambient temperature and their melting points. Ground powders, however, display broad exothermic transitions spanning roughly 15–30 °C that are not visible in subsequent heating cycles (Fig. 7). Such transitions of metastable polymorphs are often indicative of cold-crystallization from an amorphous to crystalline morphology, suggesting crystalline-to-amorphous MC transitions upon grinding.^33^,^36^,^82^–^97^ The *T*_cc_ ranges decrease with alkyl chain length from **A-1** to **A-4**, with peak maxima of 57 °C for **A-1** to 32 °C for **A-4** (Table 2). For those molecules with longer alkyl chains, the transient nature of the ground phase at room temperature yields featureless thermograms (Fig. S9†) with the exception of **A-18**, which shows an endotherm at 50–60 °C in the first heat for both annealed and ground powders, and then multiple reversible endothermic transitions in the second and third heating cycles (Fig. S10†). Such transitions have been observed in our lab in related compounds with long alkyl chains, and will be the subject of future study.

**Table 2:** Summary of **A-R** photophysical and thermal data

	Thin films Abs. *λ*_max_ (nm)			Thin films Em. *λ*_max_ (nm)			*T*_cc_ max (°C)	*T*_m_ (°C)
	Annealed	Ground	Shift	Annealed	Ground	Shift		
**A-1**	400	450	50	550	605	55	57	282 (dec)
**A-2**	420	455	35	533	595	62	54	256 (dec)
**A-3**	412	456	44	530	589	59	44	204–206
**A-4**	420	457	37	540	590	50	32	149–151
**A-6**	415	417	2	520	550	30	—	150–151
**A-8**	424	424	0	530	550	20	—	107–108
**A-18**	400	400	0	510	510	0	—	75–76
**A-1F0**	456	455	–1	597	602	5	—	201–203

In addition, heating through these exothermic transitions of **A-1** to **A-4** recovers most of the fluorescence of the annealed films, while heating to higher temperatures below the melting points completely recovers the original emission spectrum. Fig. 7 shows the visual response of the photoluminescence of films of these compounds at increasing temperatures and the gradual recovery process over a temperature range. Emission spectra show the same behavior; emission *λ*_max_ of **A-1** recovers from 605 nm to 567 nm when heated to 100 °C for 15 minutes after grinding, and heating at 220 °C results in complete reversion of the *λ*_max_ to 550 nm. Similar results are seen in **A-2**, **A-3**, and **A-4**.

Furthermore, Fig. 7 shows a gradient of recovered emission during thermal recovery where compounds with longer alkyl chain lengths show hypsochromically-shifted emission relative to those with shorter alkyl chain lengths at a given temperature. For example, in the 50 °C image in Fig. 7, **A-4** emission is the most blue-shifted and **A-1** emission is the most red-shifted. This gradient in recovered emission is reproducible when heating at a fixed temperature. Annealing at 100 °C, **A-4** achieves full recovery to emission *λ*_max_ of 545 nm, **A-3** recovers to 552 nm, **A-2** 557 nm, and **A-1** to 567 nm; this behavior persists over 5 heat–grinding cycles (Fig. S8†). In conjunction with decreasing *T*_cc_, the temperatures required for post-grinding thermochromic recovery decreases with alkyl chain length. Other families of mechanochromic compounds have shown a similar structure–property relationship.^36^,^37^,^85^,^86^,^89^,^90^,^96^,^98^

### Structural analysis of MC transition

**Fig. 8:**
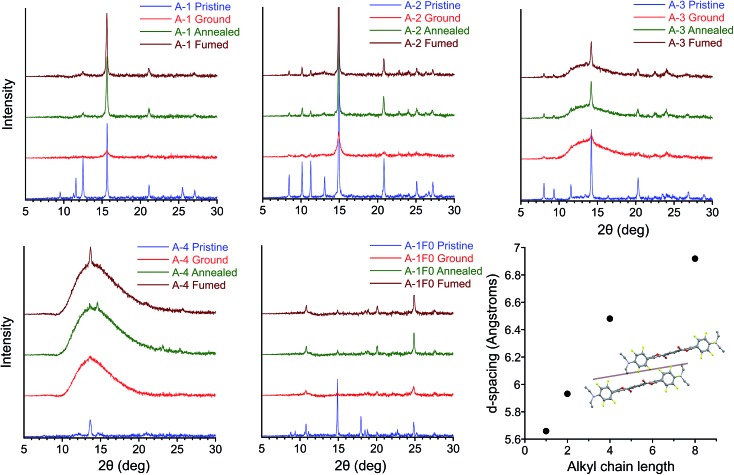
PXRD patterns of pristine, ground, annealed, and fumed films of **A-1**, **A-2**, **A-3**, **A-4**, and **A-1F0**. (Lower right) plot of alkyl chain length *vs. d*-spacing for conserved peak 2*θ* = 12.5–15.7° for **A-1**, **A-2**, **A-4**, and **A-8**, corresponding in each case to the plane of reflections lying between columns of twisted PEs.

The results of PXRD experiments are consistent with a reversible crystalline-to-amorphous transition upon grinding, which explains the nature of MC behavior in this system. Patterns were collected for films over four sequential steps (i) pristine (ii) ground (iii) thermally annealed (iv) solvent vapor annealed (fumed) after thermal annealing. X-ray diffraction of pristine and annealed films agree with simulated powder patterns from single crystal structures for **A-1**, **A-2**, **A-4**, **A-8**, and **A-1F0** with strong diffraction peaks at 2*θ* = 15.7, 14.9, 13.7, 12.8, and 24.8°, respectively. However, grinding of the films results in significantly reduced peak intensity or featureless PXRD patterns (Fig. 8). The intensities of the crystalline peaks recover after thermal and solvent annealing, which in conjunction with the exothermic transitions observed in DSC during thermal recovery, make evident that mechanical force causes a crystalline-to-amorphous transition. Subsequent heating through the *T*_cc_ range of the ground films shows some restoration of the crystalline patterns. The peaks of annealed and fumed films are significantly less intense than in the pristine film due to disruption of the crystal structure and reduction of film thickness during the grinding process, but comparison with the patterns of mechanically ground films demonstrate clear amorphous-to-crystalline recovery with annealing or vapor fuming.

Increasing alkyl chain length leads to important differences between the pristine patterns. Compounds **A-1**, **A-2**, and **A-1F0** show greater crystallinity relative to samples with longer alkyl chain lengths. We see greater amorphous character (semi-crystalline material) in the pristine patterns of **A-4**, **A-6**, **A-8**, and an almost entirely amorphous pattern in **A-18** (Fig. S11†). The increase in the amorphous nature of the films presumably originates from the weakening of intermolecular interactions across adjacent planes of PEs, particularly in structures for **A-4** and **A-8**. Control compound **A-1F0** also shows a reversible crystalline-to-amorphous transition with grinding. However, there is no change in the luminescence of the solids with grinding. The bathochromically shifted emission in both the crystalline and amorphous phases of **A-1F0**, relative to the fluorinated compounds, is consistent with our model for the MC transition in this system, in which initially twisted and non-aggregated PEs become increasingly planarized and aggregated upon application of mechanical force.^33^

Comparing the experimental and calculated patterns, it is possible to identify reflection planes and corresponding *d*-spacings from the crystal structures that are present in films. Importantly, a plane of reflections lying between columns of twisted PEs gives rise to the most intense diffraction peak 2*θ* = 15.7, 14.9, 13.7, and 12.8° in **A-1**, **A-2**, **A-4**, and **A-8**, respectively. This peak is seen in all pristine patterns except for **A-18** (Fig. S11†) and is the only peak that is recovered with significant intensity among all samples in post-grinding annealed films. This conserved peak corresponds to the well-ordered nature of twisted PE columns formed by infinite ArF–ArH stacks (Fig. 8). The relative intensity of this peak decreases with alkyl chain length in conjunction with the decreasing crystallinity of film samples, which coincides with general trends in material properties for these compounds (*vide infra*). The increasingly amorphous nature of the films is observed in the broad amorphous peak from 2*θ* = 11–15° appearing in pristine samples of **A-6** and **A-8**, and the highly amorphous pattern of **A-18**. This loss of diffraction peaks indicates that increasing alkyl chain length decreases crystallinity of powder samples to the extent that, while pristine films always display green emission corresponding to twisted, non-aggregated PEs, the long-range order of parallel columns of infinitely stacked ArF–ArH units is lost. In agreement with photophysical data, these stacks of twisted PEs are disrupted with grinding and restored with annealing/fuming. Additionally, this peak shifts to smaller 2*θ* (corresponding to greater *d*-spacing) with increasing alkyl chain length, from 5.6 Å in **A-1** to 6.9 Å in **A-8** (Fig. 8), which is consistent with looser molecular packing with longer chains that occupy more volume and result in elongated inter-chromophore distances. A peak at 2*θ* = 14.9°, corresponding to plane of reflections lying between parallel columns of coplanar, aggregated PEs is also present in the **A-1F0** diffraction pattern. It should be noted that in the case of **A-1F0** the peak at 2*θ* = 24.8°, corresponding to coplanar backbone, which contributes to the bathochromically-shifted emission in solids, is the most intense peak after recovery, whereas the peak 2*θ* = 14.9° is minimally recovered. The fact that the peak corresponding to ordered columns of PEs is significantly recovered in all cases from **A-1** through **A-8**, but not in **A-1F0**, suggests the ArF–ArH interactions are essential for such structural restoration.

The crystal structures of **A-1**, **A-2**, **A-4**, and **A-8**, confirmed with PXRD data, provide an explanation for the decrease in *T*_cc_ with increasing alkyl chain length. As alkyl chain length increases, intermolecular aromatic–π interactions are reduced (*vide supra*), and alkyl chains, with propensity for disorder, take up greater volume fractions in the structures, resulting in greater *d*-spacing between columns of PEs. These trends agree with the increasingly amorphous character of pristine films (Fig. 8 and S11†), as well as the observation of decreasing material rigidity among compounds with longer alkyl chains, the powders of which are more easily deformed with grinding. Further, these structural parameters coincide with decreasing melting points as alkyl chain length increases (282, 256, 149, and 107 °C for **A-1**, **A-2**, **A-4**, and **A-8** respectively). We propose the decrease in *T*_cc_ may originate from a complementary weakening of interactions in the amorphous phase and inhibition of close packing, which results in easier deformation of the supramolecular structures and more facile thermal restoration of the crystalline phase with increasing chain length. This is consistent with reported examples of mechanofluorochromic crystalline-to-amorphous transitions where *T*_cc_ decreases with increasing alkyl chain length due to weaker intermolecular interactions between chromophores.^85^,^89^,^90^,^98^

### Polymer hosts

In the interest of developing MC functional coatings and sensors, crystalline molecular assemblies are typically not practical for device fabrication due to their poor mechanical properties. To address this challenge, MC polymers have been reported,^12^,^99^–^101^ some with quantitation of mechanical stress.^102^ Aside from preparation of homopolymers and supramolecular polymers, other reports have demonstrated the utility of MC luminogens incorporated into polymer matrices.^26^,^103^,^104^ Considering the potential for incorporating the side-chain controlled PE design into functional polymers, we have assessed the MC behavior of the target compounds doped into various polymethacrylate hosts that differ in glass transition temperature (*T*_g_). We found that poly(methyl methacrylate) (PMMA) films doped with 20% (w/w) **A-R** and drop-cast from chloroform showed reliable MC responses in all cases with spectral shifts comparable to the pure PE films. Intriguingly, orange emission after grinding in films doped with **A-18** was persistent at room temperature, which cannot be observed in pure **A-18** films.

**Fig. 9:**
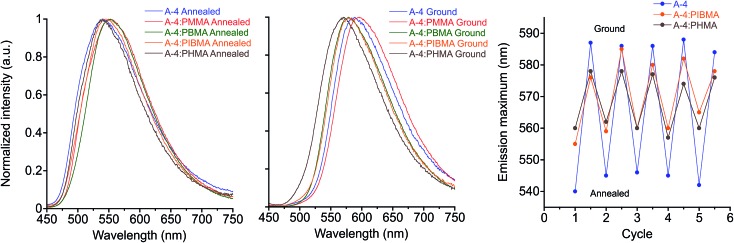
Emission spectra of **A-4** in various polymethacrylate hosts: annealed (left) and ground (center), *λ*_ex_ = 430 nm. Plot of emission maximum over five heat–grinding cycles of **A-4** and **A-4** 20% (w/w) in PIBMA, PHMA (right).

While maintaining the same conditions for preparation, MC response of these films can be maintained in other polymer hosts (Fig. 9 & S12†), though the optical shift in emission *λ*_max_ is considerably reduced. Comparison of MC properties for methacrylate films doped at 20% (w/w) with **A-4** across PMMA (*T*_g_ = 99 °C), poly(butyl methacrylate) (PBMA, *T*_g_ = 65 °C), poly(isobutyl methacrylate) (PIBMA, *T*_g_ = 20 °C), and poly(hexyl methacrylate) (PHMA, *T*_g_ = –5 °C) hosts showed a dependence of the optical shift, sensitivity, and reversibility on the host. Annealed PMMA films showed both green and orange emitting domains. This polymorphism persisted in PBMA and PIBMA films, though green emission dominated in the PIBMA film, and PHMA host yielded uniform green emission. Sensitivity to mechanical force was notably higher in PHMA films than the other hosts, and the MC transition could be achieved without breaking or deforming the film. The annealed films showed a gradual hypsochromic shift in emission with decreasing *T*_g_ of polymer host. At the same time, the MC bathochromic shift in emission decreased (Fig. 9). Furthermore, green emission in the PMMA and PBMA films could not be recovered by annealing; only the lower *T*_g_ hosts PIBMA and PHMA showed reversible MC response over multiple heating–grinding cycles, though the optical shifts in emission maxima are muted relative to **A-4** films. This variable response across the hosts indicates that thermal recovery from planarized, aggregated assemblies to the twisted, non-aggregated state depends on the relative mobility of assemblies. In a rigid host such as PMMA with high *T*_g_, the original fluorescence may not be recovered after grinding even though **A-4** normally recovers its green fluorescence within minutes at room temperature. In contrast, hosts well above the *T*_g_ at ambient temperature such as PHMA allows for facile molecular rearrangement, which in turn diminishes the extent of the bathochromic shift. These results not only demonstrate the MC capability of these PEs in polymer scaffolds, but reveal a simple means for tuning MC behavior.

## Conclusions

Seven structurally analogous three-ring PEs with dialkylaniline terminal substituents show MC behavior that is fully reversible upon thermal or solvent vapor annealing. Mechanical force causes a crystalline-to-amorphous transition as shown by DSC and PXRD, during which the fluorescence bathochromically shifts from green to orange as PE chromophores rearrange from twisted, non-aggregated assemblies as seen in single crystal structures to increasingly planarized and/or aggregated states in the amorphous phase. The MC behavior is lost in the absence of the ArF–ArH interaction, which dictates the optical properties and crystal packing of these molecules. The transition is fully reversible with heat or solvent vapor annealing, and the temperature required for thermal recovery of the crystalline phase from the ground phase decreases as the lengths of alkyl chains on the aniline substituents increase. This trend coincides with weakening intermolecular interactions in the crystal structures, allowing for greater molecular mobility of the chromophores. The MC properties of these compounds translate to polymer hosts, and can be tuned simply by changing the environment of the PE through choice of the polymer. Varying the length of alkyl substituents can be an effective method for tuning thermal recovery and material properties in small molecule MC luminogens that show crystalline-to-amorphous transitions.

Overall, this work demonstrates that this class of PEs shows robust, reversible MC behavior with structurally tunable thermochromism, elucidates details of their MC behavior, and shows how the thermal recovery depends on crystal packing, as well as how important features of the crystalline assembly in thin films respond to mechanical grinding and annealing/fuming. This knowledge will guide rational design of future MC molecular assemblies, especially in using non-covalent control to direct crystal packing in related stimuli-responsive systems.

## Conflicts of interest

The authors declare no conflicts of interest.

## Supplementary Material

Supplementary information

Crystal structure data
