# Supplementary material for: Reversible mechanofluorochromism of aniline-terminated phenylene ethynylenes
Source: Chem Sci. 2018 May 24;9(24):5415–26. doi: 10.1039/c8sc00980e (PMC6009520; doi:10.1039/c8sc00980e)
Supplement: Supplementary file 1 [file SC-009-C8SC00980E-s001.pdf]

Electronic Supplementary Material (ESI) for Chemical Science.
This journal is © The Royal Society of Chemistry 2018

Reversible Mechanofluorochromism of Aniline-Terminated Phenylene Ethynylenes

Seth A. Sharber,[a] Kuo-Chih Shih,[b] Arielle Mann,[a] Fanny Frausto,[a] Terry E. Haas,[a] Mu-Ping Nieh,[b] Samuel W. Thomas III[a]*

ELECTRONIC SUPPLEMENTARY INFORMATION

Contents

1. Experimental Methods 1
2. Synthetic Methods 3
3. NMR Data 15
4. High Resolution Mass Spectrometry 40
5. Photophysical Data in Solution 42
6. Additional crystal structure perspectives 44
7. MC Response in Thin Films 45
8. DSC Data 48
9. PXRD Data 49
10. MC Response of A-4 in polymer hosts 50
11. Crystallographic Tables 51

1. **Experimental Methods**

All synthetic manipulations were performed under standard air-free conditions under an atmosphere of argon gas with magnetic stirring unless otherwise mentioned. Flash chromatography was performed using silica gel (230-400 mesh) as the stationary phase. NMR spectra were acquired on a Bruker Avance III 500 or Bruker DPX-300 spectrometer. Melting points were measured on an SRS Digimelt MPA 160, and were recorded in triplicate to ensure melting point convergence, or by differential scanning calorimetry (DSC). High-resolution mass spectra (HRMS) were obtained at the MIT Department of Chemistry Instrumentation Facility using ESI Q-TOF and analyzed by a peak-matching protocol to determine the mass and error range of the molecular ion. All reactants and solvents were purchased from commercial suppliers and used without further purification, unless otherwise noted.

UV/Visible absorbance spectra were acquired with a Varian Cary-100 spectrophotometer in double-beam mode using a solvent-containing cuvette for background subtraction spectra when acquiring solution spectra, or a clean glass slide when acquiring spectra of thin films. Fluorescence emission spectra were obtained by using a PTI Quantum Master 4 equipped with a 75 W Xe lamp and time-correlated single photon counting (TCSPC) module. All fluorescence spectra are corrected for the output of the lamp and the dependence of detector response to the wavelength of emitted light. Absorbance and fluorescence spectra of solutions were acquired using sample absorbances less than 0.1 to minimize inner filter effects.

Relative quantum yields in dilute chloroform, dichloromethane, ethyl acetate, and tetrahydrofuran solutions were determined relative to tris(2,2′-bipyridyl)dichlororuthenium(II) hexahydrate in distilled water using the value Φ_f = 0.040 ± 0.002 reported by Suzuki et al.[1] Quantum yields in dilute toluene and hexane solutions, as well as the quantum yield for **A-1F0** in chloroform were determined relative to Coumarin 6 in ethanol using the value φ_f = 0.78 reported by Reynolds and Drexhage.[2] Fluorescence lifetimes were determined using a 403 nm pulsed LED light source for time correlated single photon counting (TCSPC). Ludox (diluted in deionized water) was used to determine the instrument response function. Data was analyzed using FelixGX version 4 data analysis, with 1 to 4 exponential lifetime analysis.

Absorbance and fluorescence spectra of solutions were recorded in chloroform unless otherwise noted. Absorbance and fluorescence spectra of solids were recorded from films of materials that had been drop-cast from solutions in chloroform onto quartz slides (2 mg / mL), which were evaporated slowly under a watch glass. Spin-coated films were prepared from chloroform solutions (2 mg / mL). Film samples were ground by rubbing with a spatula until the bathochromic shift was observed by fluorescence. Films were annealed in a pre-heated oven for 15 minute cycles at specified temperatures unless otherwise noted. Films were solvent vapor annealed by suspending in a closed jar with a small volume of dichloromethane for 60 s.

Crystals were grown in 1.0 and 1.5 dram vials by slow evaporation from chloroform or dichloromethane/hexanes solutions over the course of days, or by slow diffusion of hexanes into chloroform. Specific details on crystallization are given in the synthetic methods section. Single crystal fluorescence spectroscopy (emission and excitation) probed samples from the same crystallization trial of the crystal chosen for X-ray diffraction or samples grown by the same method and demonstrating visually identical fluorescence and having similar appearance.

Low-temperature single crystal diffraction data were collected on a Bruker D8 Quest diffractometer coupled to a Photon CMOS detector with Mo Kα radiation (λ = 0.71073 Å) from a fine-focused sealed tube source for crystal sample of **A-2**. Data were collected on a Bruker-AXS X8 Kappa Duo four-circle diffractometer coupled to a Smart Apex2 CCD detector with Cu Kα radiation ((λ = 1.54184 Å) from an IμS micro-source for samples of **A-1** (previously reported), **A-4**, and **A-8** in collaboration with Peter Müller and the MIT X-Ray Diffraction Facility, performing φ- and ω-scans in all cases. Data collection on this instrument is possible through the generous support of the National Science Foundation under grant CHE-0946721. The structures were solved by direct methods using SHELXS-97[3] and refined against F^2 on all data by full-matrix least squares with SHELXL-2014[4,5] following established refinement strategies.[6] With the exception of **A-4**, all non-hydrogen atoms were refined anisotropically; all hydrogen atoms were included into the model at geometrically calculated positions and refined using a riding model. The isotropic displacement parameters of all hydrogen atoms were fixed to 1.2 times the U value of the atoms they are linked to (1.5 times for methyl groups).

In the **A-4** structure, whole molecule disorder was observed and the positions of all carbons in butyl chains could not be fully solved and refined. Butyl chains were modeled for disorder and refined anisotropically where possible with inclusion of hydrogens. Butyl chains that could not be fully solved where disorder was significant were refined isotropically, and multiple butyl chains were either left incomplete or without complete bond connectivity. A level alert PLAT082_ALERT_2_A and PLAT084_ALERT_3_A for high R1 and wR2 values are present due to poor data quality, whole molecule disorder, and difficulty in high-quality modeling of the data. In addition, CHEMW03_ALERT_2_A and PLAT043_ALERT_1_A alerts are present for significant differences in the calculated and reported molecular weights, given that positions of all atoms could

not be discretely solved and anisotropically refined, resulting in an incorrect chemical formula sum. Alerts PLAT097_ALERT_2_A, PLAT234_ALERT_4_A, PLAT601_ALERT_2_A, and PLAT934_ALERT_3_A were similarly observed for issues with positive residual density, large Hirshfield difference, solvent accessible voids, and (Iobs-Icalc)/SigmaW outliers, which all originate from the low data quality and lack of completeness in solving and refining the disordered butyl chains, in addition to whole molecule disorder. Despite the relatively low quality of the data and resulting model, the crystal structure clearly shows the twisted nature of the PEs with ArF-ArH interactions, as well as volume occupied by electron density from alkyl chains rather than PE chromophores and resulting decrease in aromatic-π interactions between neighboring stacks of PEs relative to the **A-1** structure. This effect is intensified in the **A-8** structure.

In the **A-8** structure, disorder was modeled in two of the octyl chains, and all non-hydrogen atoms were refined anisotropically and hydrogens included into the model as described above. Thermal ellipsoids near the ends of octyl chains were elongated, giving rise to A-level alert PLAT213 for atom C85A given significant disorder and thermal motion in the chains.

Differential scanning calorimetry (DSC) was performed on a TA Discovery DSC 250 equipped with a finned-air cooling system (FACS). Scans were recorded in TZero pans sealed with TZero lids at a rate of 10 °C/min. Pristine powder samples (1-2 mg) were loaded into pans. For ground powder experiments, powders were ground upon glass slides silanized with (3-aminopropyl)triethoxysilane (APTES), then loaded into TZero pans. Grinding powders upon the non-stick side of Scotch tape gave optimal results for sample loading.

Powder X-Ray Diffraction (PXRD) data was recorded at the Institute of Materials Science's X-ray lab at University of Connecticut on a Bruker D2 PHASER 1D PXRD instrument. The generator was set to a voltage of 30 kV and current of 10 mA. Scans were recorded using Cu Kα radiation ((λ = 1.54184 Å) across 2-Theta values 5 – 50° with step size 0.02° and integration time of 1 s. Samples were prepared by drop-casting films from saturated chloroform solutions onto glass microscope cover slips. Glass microscope cover slip background signal was subtracted from experimental scans.

2. Synthetic Methods

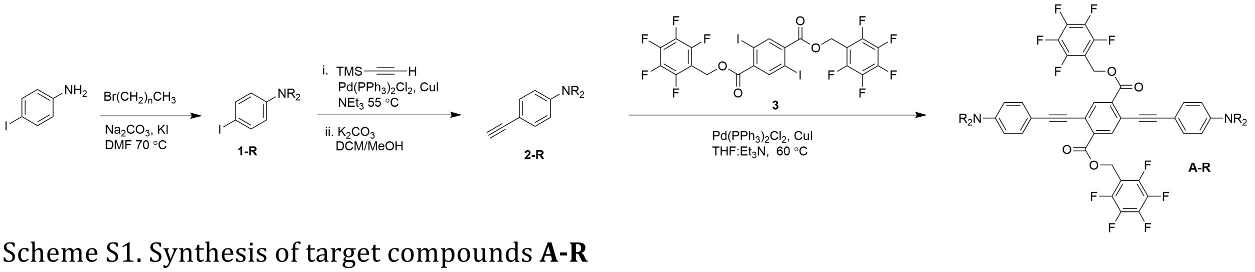

Scheme S1. Synthesis of target compounds **A-R**

General procedure for syntheses of **1-R**

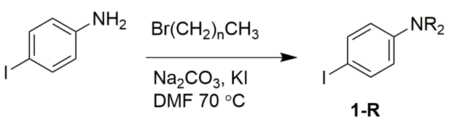

The procedure to make these compounds was adapted from the literature.[7] Into a 100 mL 2-neck round bottom flask equipped with a reflux condenser was added the alkyl bromide, potassium

iodide, and anhydrous, argon-sparged DMF (resulting in 0.5 M 4-iodoaniline). The mixture was stirred for 30 min. at 95 °C. The 4-iodoaniline (1.0 eq.) and sodium carbonate were added, and the heterogeneous mixture stirred at 95 °C 18 – 26 h, unless otherwise noted. Upon consumption of the 4-iodoaniline as shown by TLC, the mixture was cooled to room temperature and diluted with ethyl acetate. The mixture was poured over water and extracted with ethyl acetate. The combined organic layers were washed with water and brine, dried over magnesium sulfate, vacuum filtered, and evaporated *in vacuo* giving a brown oil. The oil was purified by flash chromatography on silica gel to give a colorless oil.

General procedure for syntheses of **2-R**

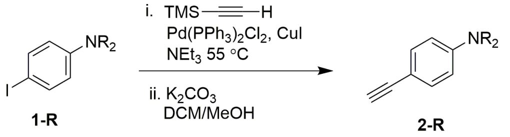

This procedure for ethynylation and subsequent deprotection was adapted from the literature.[8,9] To a 50 mL 2-neck round bottom flask equipped with condenser was added **1-R**, trimethylsilyl acetylene, 5 mol% $Pd(PPh_3)_2Cl_2$, and 10 mol% CuI dissolved in deoxygenated 3:1 v/v Et_3N:THF. The mixture was stirred 16 h at 60 °C. Upon consumption of the aryl iodide, the mixture was cooled to RT, filtered through Celite, immobilized on silica gel, and purified by flash column chromatography. The resulting oil was dissolved in 1:10 v/v DCM:MeOH with potassium carbonate (2 eq.) and stirred 2 h at room temperature, unless otherwise noted. The mixture was poured over water and extracted with DCM. The combined organic layers were washed with water and brine, dried over magnesium sulfate, filtered, and evaporated *in vacuo* giving a green or orange oil and the product was carried on without further purification.

General procedure for synthesis of **A-R**

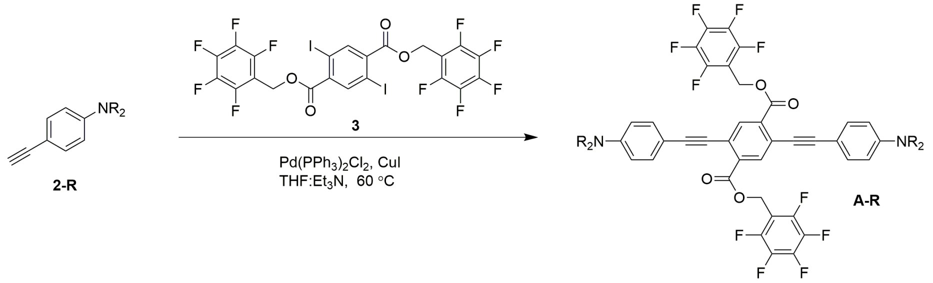

This procedure was performed as previously reported[10]. To a Schlenk tube or oven-dried round bottom flask with condenser was added **3**, 2-5 mol% $Pd(PPh_3)_2Cl_2$, 4-10 mol% CuI, and **2-R** dissolved in 1:1 (v/v) deoxygenated THF:NEt_3. The mixture was stirred at 60 °C for 16 – 24 h unless otherwise noted. Upon reaction completion (shown by flowing white precipitate and a fluorescent product spot by TLC) the mixture was cooled to room temperature, filtered through Celite, and evaporated *in vacuo*. The resulting powder was adsorbed onto silica gel and purified by flash column chromatography. The yellow powder was further purified by recrystallization from DCM/hexanes unless otherwise noted.

1-2

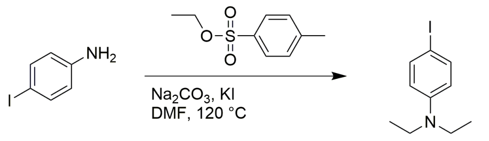

This reaction followed the general procedure for **1-R** using 3.25 g (16.2 mmol, 4.4 eq.) ethyl *p*-toluene sulfonate (rather than 1-bromoethane), 2.75 g potassium iodide (15.0 mmol, 3.3 eq.), 1.62 g (15.2 mmol, 4.2 eq.) sodium carbonate, and 802 mg (3.66 mmol, 1.0 eq.) 4-iodoaniline in 12 mL DMF. The mixture was stirred 120 h at 120 °C, worked up as described in the general procedure, and purified by flash chromatography on silica gel (1:1 DCM:hexanes, 0.2% Et_3N) giving 503 mg colorless oil in 50% yield.

^{1}H NMR (500 MHz, $CDCl_3$) δ: 7.47 (d, J = 9.0 Hz, 2H), 6.49 (d, J = 10.0 Hz, 2H), 3.35 (q, J = 7.1 Hz, 4H), 1.18 (t, J = 7.1 Hz, 6H) in agreement with the literature.[11]

1-3

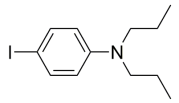

This reaction followed the general procedure for **1-R** using 1.80 mL (19.8 mmol, 4.3 eq.) 1-bromopropane, 2.75 g potassium iodide (15.1 mmol, 3.3 eq.), 1.48 g (13.9 mmol, 3.0 eq.) sodium carbonate, and 1.00 g (4.57 mmol, 1.0 eq.) 4-iodoaniline in 10 mL DMF. The mixture was stirred 25 h at 70 °C, worked up as described in the general procedure, and purified by flash chromatography on silica gel (1:19 DCM:hexanes, 0.2% Et_3N) giving 887 mg colorless oil in 50% yield.

^{1}H NMR (500 MHz, $CDCl_3$) δ: 7.32 (d, J = 9.1 Hz, 2H), 6.32 (d, J = 9.2 Hz, 2H), 3.11 (t, J = 7.7 Hz, 4H), 1.49 (sextet, J = 7.5 Hz, 4H), 0.83 (t, J = 7.4 Hz, 6H)

^{13}C NMR (125 MHz, $CDCl_3$) δ: 147.7, 137.7, 114.0, 75.4, 52.8, 20.3, 11.5 in agreement with the literature.[12]

1-4

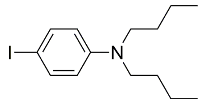

This reaction followed the general procedure for **1-R** using 1.80 mL (16.7 mmol, 3.6 eq.) 1-bromobutane, 2.50 g potassium iodide (15.1 mmol, 3.3 eq.), 867 mg (8.18 mmol, 1.8 eq.) sodium carbonate, and 1.00 g (4.58 mmol, 1.0 eq.) 4-iodoaniline in 10 mL DMF. The mixture was stirred 26 h at 95 °C, worked up as written, and purified by flash chromatography on silica gel (hexanes, then gradient to 3:17 DCM:hexanes) giving 853 mg colorless oil in 56% yield.

^{1}H NMR (500 MHz, $CDCl_3$) δ: 7.42 (d, J = 9.1 Hz, 2H), 6.42 (d, J = 9.0 Hz, 2H), 3.23 (t, J = 7.6 Hz, 4H), 1.54 (quintet, J = 7.6 Hz, 4H), 1.34 (sextet, J = 7.4 Hz, 4H), 0.96 (t, J = 7.3 Hz, 6H)

^{13}C NMR (125 MHz, $CDCl_3$) δ: 147.7, 137.7, 114.1, 75.4, 50.8, 29.2, 20.4, 14.0 in agreement with the literature.[13]

1-6

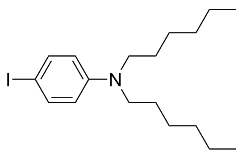

This reaction followed the general procedure for **1-R** using 4.23 mL 1-bromohexanes (30.2 mmol, 3.3 eq.), 5.01 g potassium iodide (30.2 mmol, 3.3 eq.), 1.74 g (16.4 mmol, 1.8 eq.) sodium carbonate, and 2.00 g (9.13 mmol, 1.0 eq.) 4-iodoaniline in 10 mL DMF. The mixture was stirred 20 h at 115 °C, worked up as described in the general procedure, and purified by flash chromatography on silica gel (1:19 DCM:hexanes, 0.2% Et_3N) giving 936 mg colorless oil in 35% yield.

^{1}H NMR (500 MHz, $CDCl_3$) δ: 7.43 (d, J = 9.1 Hz, 2H), 6.42 (d, J = 9.1 Hz, 2H), 3.24 (t, J = 7.7 Hz 4H), 1.54 (m, 4H), 1.33 (m, 12H), 0.93 (m, 6H) in agreement with the literature.[14]

1-8

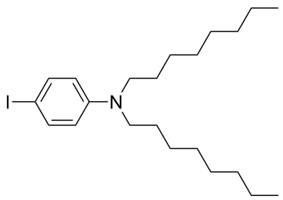

This reaction followed the general procedure for **1-R** using 2.40 mL (15.8_mmol, 3.5 eq.) 1-bromooctane, 2.639 g (15.9 mmol, 3.5 eq.) potassium iodide, 910 mg (8.59 mmol, 1.9 eq.) sodium carbonate, and 998 mg (4.56 mmol, 1.0 eq.) 4-iodoaniline in 10 mL DMF. The mixture was stirred 21 h at 95 °C, worked up as described in the general procedure, and purified by flash chromatography on silica gel (hexanes) giving 1.48 g colorless oil in 73% yield.

^{1}H NMR (500 MHz, $CDCl_3$) δ: 7.44 (d, J = 9.0 Hz, 4H), 6.43 (d, J = 9.8 Hz, 4H), 3.23 (t, J = 7.7 Hz, 4H), 1.57 (m, 4H), 1.32 (m, 20H), 0.92 (t, J = 6.9Hz 6H).

^{13}C NMR (125 MHz, $CDCl_3$) δ: 147.6, 137.6, 114.0, 75.3, 51.0, 31.8, 29.5, 29.3, 27.1, 27.0, 22.7, 14.1 in agreement with the literature.[15]

1-18

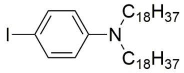

This reaction followed the general procedure for **1-R** using 4.95 g (14.9 mmol, 3.1 eq.) 1-bromooctadecane, 2.670 g (16.08 mmol, 3.4 eq.) potassium iodide, 1.97 g (18.6 mmol, 3.9 eq.) sodium carbonate, and 1.05 g (47.8 mmol, 1.0 eq.) 4-iodoaniline in 10 mL DMF. The mixture was stirred 120 h at 115 °C, worked up as described in the general procedure, and purified by flash chromatography on silica gel (hexanes) giving 680 mg colorless solid in 21 % yield.

^{1}H NMR (500 MHz, $CDCl_3$) δ: 7.43 (d, J = 9.2 Hz, 2H), 6.42 (d, J = 8.9 Hz, 2H), 3.23 (t, J = 7.7 Hz, 4H), 1.56 (s, 2H), 1.31-1.28 (m, 62H), 0.91 (t, J = 6.9 Hz, 6H)

^{13}C NMR (125 MHz, $CDCl_3$) δ: 147.6, 137.6, 114.0, 75.3, 51.0, 31.9, 29.7, 29.6, 29.5, 29.4, 29.1, 27.1, 22.7, 14.4.

2-2

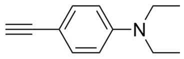

This reaction followed the general procedure for **2-R** using 400 mg (1.45 mmol) **1-2**, 0.68 mL (4.9 mmol, 3.4 eq.) trimethylsilylacetylene, 52 mg (0.074 mmol, 5 mol%) $Pd(PPh_3)_2Cl_2$, and 31 mg (0.16 mmol, 11 mol%) CuI in 10 mL deoxygenated 3:1 v/v Et_3N:THF stirred 17 h at 50 °C. The resulting oil was purified by flash chromatography on silica gel (1:1 DCM:hexanes, 0.2% Et_3N) giving 334 mg TMS protected product. 120 mg of the product was deprotected following the general procedure for 2-**R** using 137 mg (0.993 mmol, 2.0 eq.) potassium carbonate in 7 mL 1:10 DCM:MeOH, stirred 2 h at room temperature. The mixture was worked up as described in the general procedure giving 82 mg oil in 90% overall yield, which was carried on without further purification.

^1H NMR (300 MHz, $CDCl_3$) δ: 7.33 (d, J = 8.9 Hz, 2H), 6.56 (d, J = 8.9 Hz, 2H), 3.34 (q, J = 7.1 Hz, 4H), 2.96 (s, 1H), 1.16 (t, J = 7.1 Hz, 6H) in agreement with the literature.[16]

2-3

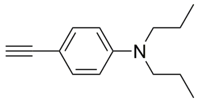

This reaction followed the general procedure for **2-R** using 400 mg (1.32 mmol) **1-3**, 0.60 mL (4.3 mmol, 3.3 eq.) trimethylsilylacetylene, 45 mg (0.064 mmol, 5 mol%) $Pd(PPh_3)_2Cl_2$, and 28 mg (0.15 mmol, 11 mol%) CuI in 10 mL deoxygenated 3:1 v/v Et_3N:THF stirred 23 h at 50 °C. The resulting oil was purified by flash chromatography on silica gel (1:4 DCM:hexanes, 0.2% Et_3N) giving 289 mg TMS protected product. The entirety of the product was deprotected using 327 mg (2.37 mmol, 2.2 eq.) potassium carbonate in 12 mL 1:10 DCM:MeOH, stirred 2 h at room temperature. The mixture was worked up as described in the general procedure giving 192 mg oil in 72% overall yield, which was carried on without further purification.

^1H NMR (500 MHz, $CDCl_3$) δ: 7.35 (d, J = 9.0 Hz, 2H), 6.57 (d, J = 9.1 Hz, 2H), 3.26 (t, J = 7.7 Hz, 4H), 2.99 (s, 1H), 1.64 (sextet, J = 7.5 Hz, 4H), 0.96 (t, J = 7.4 H, 6H)

^{13}C (125 MHz, $CDCl_3$) δ: 148.3, 133.4, 111.1, 107.4, 85.1, 74.5, 52.7, 20.4, 11.4 in agreement with the literature.[12]

2-4

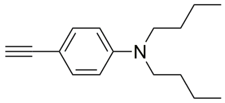

This reaction followed the general procedure for **2-R** using 600 mg (1.81 mmol) **1-4**, 0.55 mL (4.0 mmol, 2.2 eq.) trimethylsilylacetylene, 64 mg (0.09 mmol, 5 mol%) $Pd(PPh_3)_2Cl_2$, and 34 mg (0.18 mmol, 10 mol%) CuI in 10 mL deoxygenated 3:1 v/v Et_3N:THF stirred 41 h at 60 °C. The resulting

oil was purified by flash chromatography on silica gel (1:9 DCM:hexanes, then gradient to 3:7 DCM:hexanes) giving 442 mg TMS protected product. 150 mg of the product was deprotected using 224 mg (1.35 mmol, 2.0 eq.) potassium carbonate in 12 mL 1:10 DCM:MeOH, stirred 4 h at room temperature. The mixture was worked up as described in the general procedure giving 89 mg oil in 44% overall yield, which was carried on without further purification.

^{1}H NMR (500 MHz, $CDCl_3$) δ: 7.34 (d, J = 9.0 Hz, 2H), 6.55 (d, J = 9.0 Hz, 2H), 3.29 (t, J = 7.7 Hz, 4H), 2.99 (s, 1H), 1.56 (m, 4H), 1.35 (sextet, J = 7.5 Hz, 4H), 0.98 (t, J = 7.4 Hz, 6H)

^{13}C NMR (125 MHz, $CDCl_3$) δ: 148.2, 133.4, 111.0, 85.1, 74.4, 50.7, 29.3, 20.3, 14.0 in agreement with the literature.[17]

2-6

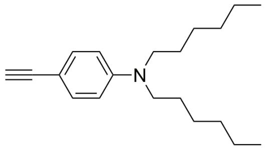

This reaction followed general procedure for **2-R** using 750 mg (1.94 mmol) **1-6**, 0.80 mL (5.8 mmol, 3.0 eq.) trimethylsilylacetylene, 68 mg (0.10 mmol, 5 mol%) $Pd(PPh_3)_2Cl_2$, and 37 mg (0.19 mmol, 10 mol%) CuI in 15 mL deoxygenated 3:1 v/v Et_3N:THF stirred 70 h at 60 °C. The resulting oil was purified by flash chromatography on silica gel (1:9 DCM:hexanes, 0.2% Et_3N) giving 551 mg TMS protected product. 197 mg of the product was deprotected using 270 mg (1.95 mmol, 3.6 eq.) potassium carbonate in 14 mL 1:10 DCM:MeOH, stirred 5 h at room temperature. The mixture was worked up as described in the general procedure giving 147 mg oil in 75% overall yield, which was carried on without further purification.

^{1}H NMR (500 MHz, $CDCl_3$) δ: 7.35 (d, J = 9.0 Hz, 2H), 6.56 (d, J = 9.0 Hz, 2H), 3.28 (t, J = 7.7 Hz, 4H), 2.99 (s, 1H), 1.59 (m, 4H), 1.34 (m, 12H), 0.94 (m, 6H)

^{13}C NMR (125 MHz, $CDCl_3$) δ: 148.2, 133.4, 111.0, 107.3, 85.1, 74.4, 50.9, 31.7, 27.2, 26.8, 22.7, 14.1 in agreement with the literature.[18]

2-8

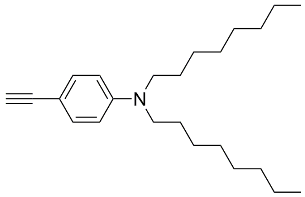

This reaction followed the general procedure for **2-R**, except for deprotection of TMS which proceeded according to a literature procedure.[17] Ethynylation was executed using 791 mg (1.78 mmol, 1.0 eq.) **1-8**, 0.65 mL (4.7 mmol, 2.6 eq.) trimethylsilylacetylene, 68 mg (0.10 mmol, 5 mol%) $Pd(PPh_3)_2Cl_2$, and 40 mg (0.21 mmol, 12 mol%) CuI in 12 mL deoxygenated 3:1 v/v Et_3N:THF stirred 17 h at room temperature. The resulting oil was purified by flash chromatography on silica gel (1:9 DCM:hexanes, then gradient to 3:7 DCM:hexanes) giving 644 mg TMS protected product. 300 mg of the product was deprotected using 1.4 mL of 35% KOH in water, dissolved in 1:5 v/v THF:MeOH. The mixture was stirred 5 h at 60 °C The mixture was worked up as described, then

purified by flash chromatography on silica gel (1:9 DCM:hexanes) giving 197 mg oil in 69% overall yield.

^{1}H NMR (500 MHz, $CDCl_3$) δ: 7.36 (d, J = 9.0 Hz, 2H) 6.55 (d, J = 9.0 Hz, 2H), 3.27 (t, J = 7.7 Hz, 4H), 2.99 (s, 1H), 1.61-1.58 (m, 4H), 1.34-1.31 (m, 20H), 0.93 (t, J = 6.8 Hz, 6H).

^{13}C NMR (125 MHz, $CDCl_3$) δ: 148.2, 133.4, 111.0, 107.4, 85.1, 74.4, 51.0, 31.86, 29.5, 29.4, 27.2, 27.2, 22.7, 14.1 in agreement with the literature.[15]

2-18

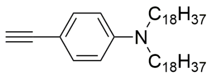

This reaction followed the general procedure for **2-R** using 400 mg (0.550 mmol, 1.0 eq.) **1-18**, 0.23 mL (1.7 mmol, 3.0 eq.) trimethylsilylacetylene, 19 mg (0.03 mmol, 5 mol%) $Pd(PPh_3)_2Cl_2$, and 11 mg (0.06 mmol, 10 mol%) CuI in 9 mL deoxygenated 3:1 v/v Et_3N:THF stirred 23 h at 70 °C. The resulting oil was purified by flash chromatography on silica gel (1:19 DCM:hexanes, then gradient to 3:17 DCM:hexanes) giving 325 mg TMS protected product. 122 mg of the product was deprotected using 400 mg (2.89 mmol, 17 eq.) potassium carbonate in 2.5 mL 1:10 DCM:MeOH, stirred 16 h at room temperature. The mixture was worked up as described in the general procedure giving 92 mg yellow oil in 72% overall yield, which was carried on without further purification.

^{1}H NMR (500 MHz, $CDCl_3$) δ: 7.34 (d, J = 9.0 Hz, 2H), 6.55 (d, J = 9.0 Hz, 2H), 3.3 (m, J = 7.7 Hz, 4H), 2.98 (s, 1H), 1.58 (m 4H), 1.33-1.29 (m, 60H), 0.90 (t, J = 7.0 Hz, 6H).

^{13}C NMR (125 MHz, $CDCl_3$) δ: 148.2, 133.4, 133.3, 111.0, 107.4, 85.1, 74.4, 50.9, 31.9, 29.7 29.6, 29.5, 29.4, (unresolved peaks 29.7-29.4) 27.2, 27.1, 22.7, 14.1

3

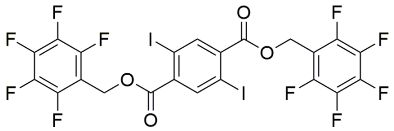

This synthesis has been reported previously.[10,19]

3-F0

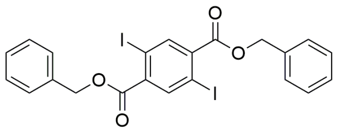

This synthesis has been reported previously.[20]

A-1

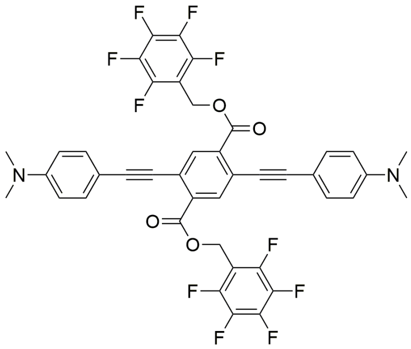

This synthesis has been reported previously .[10]

A-2

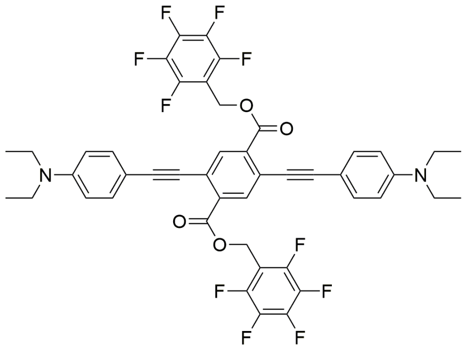

This reaction followed the general procedure for **A-R** with 100 mg (0.129 mmol, 1.0 eq.) **3**, 7 mg (0.01 mmol, 8 mol%) $Pd(PPh_3)_2Cl_2$, 5 mg (0.03 mmol, 23 mol%) CuI, and 53 mg (0.31 mmol, 2.4 eq.) **2-2** dissolved in 20 mL 1:1 (v/v) deoxygenated THF:NEt_3. The mixture was stirred 16 h at 60 °C. The resulting powder was purified by flash chromatography on silica gel (3:2 DCM:hexanes, 0.2% Et_3N), then recrystallized giving 64 mg yellow powder with green fluorescence in 57% yield. Melting point 256 °C (dec). Yellow needle with green fluorescence for single-crystal X-ray diffraction grown from slow evaporation of chloroform.

^{1}H NMR (500 MHz, $CDCl_3$) δ: 8.13 (s, 2H), 7.28 (d, J = 9 Hz, 2H), 6.61 (d, J = 9 Hz, 2H), 5.49 (s, 4H), 3.42 (q, J = 7 Hz, 8H), 1.22 (t, J = 7 Hz, 12H)

^{19}F NMR (470 MHz, $CDCl_3$) δ: -141.4 (dd, J1 = 22 Hz, J2 = 8 Hz), -152.3 (t, HJ = 21 Hz), -161.4 (td, J1 = 21 Hz, J2 = 7 Hz)

^{13}C NMR (125 MHz, $CDCl_3$) δ: 164.6, 148.1, 145.8 (d, J = 251 Hz), 141.8 (d, J = 255 Hz), 137.4 (d, J = 253 Hz), 136.0, 133.0, 132.6, 123.2, 111.0, 109.1, 108.0, 99.2, 85.5, 54.1, 44.4, 12.5

HRMS (ESI QTOF) calculated for $C_{46}H_{34}F_{10}N_2O_4$ [M+H]: 869.2437, found [M+H]: 869.2428

A-3

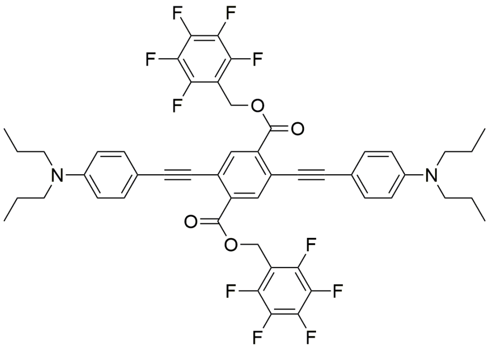

This reaction followed the general procedure for **A-R** with 102 mg (0.130 mmol, 1.0 eq.) **3**, 5 mg (0.007 mmol, 5 mol%) $Pd(PPh_3)_2Cl_2$, 8 mg (0.04 mmol, 32 mol%) CuI, and 57 mg (0.28 mmol, 2.2 eq.) **2-3** dissolved in 20 mL 1:1 (v/v) deoxygenated THF:NEt_3. The mixture was stirred 16 h at 60 °C. The resulting powder was purified by flash chromatography on silica gel (2:3 DCM:hexanes), then recrystallized giving 70 mg yellow powder with green fluorescence in 59% yield. Melting point 203.5 – 205.8 °C.

^{1}H NMR (500 MHz, $CDCl_3$) δ: 8.03 (s, 2H), 7.16 (d, J = 8.8 Hz, 4H), 6.47 (d, J = 8.8 Hz, 4H), 5.39 (s, 4H), 3.20 (t, J = 7.7 Hz, 8H), 1.56 (sextet, J = 7.5 Hz, 8H), 0.88 (t, J = 7.4 Hz, 12H)

^{19}F NMR (470 MHz, $CDCl_3$) δ: -141.5 (q, J = 10 Hz), -152.3 (t, J = 21 Hz), -161.4 (dt, J1 = 22 Hz, J2 = 8 Hz)

^{13}C NMR (125 MHz, $CDCl_3$) δ: 164.6, 148.5, 145.8 (d, J = 251 Hz), 141.8 (d, J = 256 Hz), 137.5 (d, J = 253 Hz), 136.5, 136.01, 132.9, 123.2, 111.1, 109.1 (dt, J1 = 17 Hz, J2 = 4 Hz), 107.9, 99.2, 85.5, 54.1, 52.8, 20.4, 11.4

HRMS (ESI QTOF) calculated for $C_{50}H_{42}F_{10}N_2O_4$ [M+H]: 925.3063, found [M+H]: 925.3058

A-4

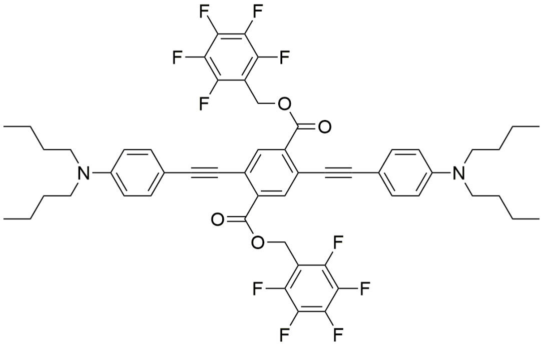

This reaction followed the general procedure for **A-R** with 97 mg mg (0.15 mmol, 1.0 eq.) **3**, 11 mg (0.016 mmol, 10 mol%) $Pd(PPh_3)_2Cl_2$, 6 mg (0.032 mmol, 20 mol%) CuI, and 98 mg (0.43 mmol, 2.8 eq.) **2-4** dissolved in 20 mL 1:1 (v/v) deoxygenated THF:NEt_3. The mixture was stirred 72 h at 60 °C. The resulting powder was purified by flash chromatography on silica gel (3:7 DCM:hexanes, then gradient to 1:1 DCM:hexanes, 0.2% Et_3N), then recrystallized giving 32 mg yellow powder with green fluorescence in 26% yield. Melting point 149.3-150.5 °C. Yellow needles with green fluorescence for single-crystal X-ray diffraction grown from slow diffusion of hexanes into a solution of chloroform.

^{1}H NMR (500 MHz, $CDCl_3$) δ: 8.13 (s, 2H), 7.26 (d, J = 8.7 Hz, 2H), 6.57 (d, J = 8.7 Hz, 2H), 5.49 (s, 4H), 3.32 (t, J = 7.7 Hz, 8H), 1.43 (m, 8H), 1.39 (m, 8H), 1.00 (t, J = 7.4 Hz, 12H)

^{19}F NMR (282 MHz, $CDCl_3$) δ: -141.5 (q, J = 10 Hz), -152.3 (t, J = 21 Hz), -161.4 (dt, J_1 = 21 Hz, J_2 = 8 Hz)

^{13}C NMR (125 MHz, $CDCl_3$) δ: 164.64, 145.8 (d, J = 253.5 Hz), 141.8 (d, J = 257.0 Hz), 137.5 (d, J = 253.5 Hz), 123.16, 111.05, 109.08 (t, J = 15.4 Hz), 107.81, 99.20, 85.48, 54.11, 50.71, 29.31, 20.31, 13.97

HRMS (ESI QTOF) calculated for $C_{54}H_{50}F_{10}N_2O_4$ [M+H]: 981.3689, found [M+H]: 981.3688

A-6

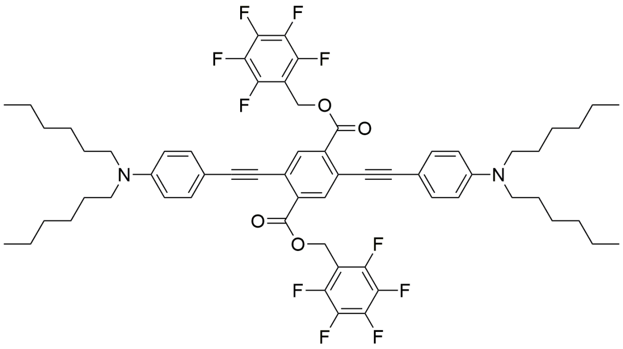

This reaction followed the general procedure for **A-R** with 97 mg (0.13 mmol, 1.0 eq.) **3**, 7 mg (0.01 mmol, 8 mol%) $Pd(PPh_3)_2Cl_2$, 5 mg (0.03 mmol, 20 mol%) CuI, and 81 mg (0.28 mmol, 2.3 eq.) **2-6** dissolved in 20 mL 1:1 (v/v) deoxygenated THF:NEt_3. The mixture was stirred 21 h at 60 °C. The resulting powder was purified by flash chromatography on silica gel (1:3 DCM:hexanes, then gradient to 3:7 DCM:hexanes, 0.5% Et_3N), then recrystallized giving 67 mg yellow powder with green fluorescence in 49% yield. Melting point 149.9-159.9 °C.

^{1}H NMR (500 MHz, $CDCl_3$) δ: 8.11 (s, 2H), 7.24 (d, J = 8.8 Hz, 4H), 6.54 (d, J = 9.0 Hz, 4H), 5.47 (s, 4H), 3.29 (t, J = 7.7 Hz, 8H), 1.60 (m, 8H), 1.34 (m, 24H), 0.92 (t, 12H)

^{19}F NMR (470 MHz, $CDCl_3$) δ: -141.4 (dd, J1 = 22.6 Hz, J2 = 7.9 Hz), -152.3 (t, J = 20.9 Hz), -161.4 (td, J1 = 21.5 Hz, J2 = 7.3 Hz)

^{13}C NMR (125 MHz, $CDCl_3$) δ: 164.6, 148.4, 145.8 (d, J = 245 Hz), 141.9 (d, J = 254 Hz), 137.5 (d, J = 252 Hz), 136.0, 132.9, 132.6, 123.2, 111.0, 109.1 (t, J = 18 Hz), 107.8 99.2, 85.5, 54.1, 51.0, 31.7, 27.1, 26.8, 22.7, 14.0

HRMS (ESI QTOF) calculated for $C_{62}H_{66}F_{10}N_2O_4$ [M+H]: 1093.4941, found [M+H]: 1093.4954

A-8

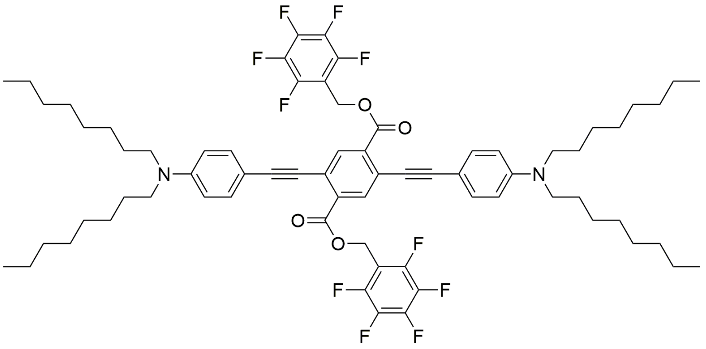

This reaction followed the general procedure for **A-R** with 101 mg (0.130 mmol, 1.0 eq.) **3**, 6 mg (0.009 mmol, 7 mol%) $Pd(PPh_3)_2Cl_2$, 8 mg (0.04 mmol, 32 mol%) CuI, 94 mg (0.28 mmol, 2.1 eq.) **2-8** dissolved in 23 mL 1:1 (v/v) deoxygenated THF:NEt_3. The mixture was stirred 17 h at 60 °C. The resulting powder was purified by flash chromatography on silica gel (1:4 DCM:hexanes) giving 124 mg yellow powder with green fluorescence in 89% yield. Melting point 107.1-108.3 °C. Yellow needles with green fluorescence for single-crystal X-ray diffraction grown from slow evaporation of a solution of dichloromethane with layered hexanes.

^{1}H NMR (500 MHz, $CDCl_3$) δ: 8.13 (s, 2H), 7.28 (d, J = 8.8 Hz, 4H), 6.56 (d, J = 8.9 Hz, 4H), 5.49 (s, 4H), 3.31 (t, J = 7.7 Hz, 8H), 1.62 (m, 8H), 1.36 - 1.32 (m, 40H), 0.93 (t, J = 6.9 Hz, 6H)

^{19}F NMR (470 MHz, $CDCl_3$) δ: -141.5 (q, J = 10 Hz), -152.57 (t, J = 21 Hz), -161.3 (dt, J_1 = 21 Hz, J_2 = 7 Hz)

^{13}C NMR (125 MHz, $CDCl_3$) δ: 164.6, 148.5, 145.8 (d, J = 252 Hz), 141.8 (d, J= 256 Jz), 137.5 (d, J = 252 Hz), 136.0, 132.9, 132.6, 123.2, 111.0, 109.1 (t, J = 19 Hz), 107.8, 99.2, 85.5, 54.1, 51.0, 31.8, 29.5, 29.3, 27.2, 27.1, 22.7, 14.1

HRMS (ESI QTOF) calculated for $C_{70}H_{82}F_{10}N_2O_4$ [M+H]: 1205.6193, found [M+H]: 1205.6187

A-18

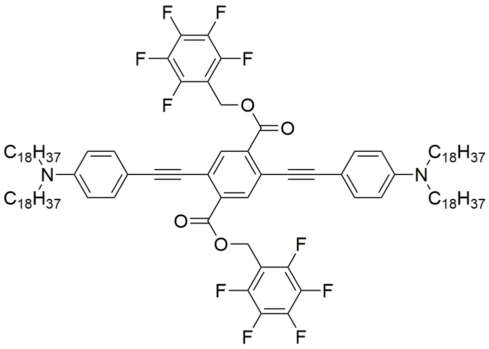

This reaction followed the general procedure for **A-R** with 47 mg (0.060 mmol, 1.0 eq.) **3**, 10 mg (0.014 mmol, 24 mol%) $Pd(PPh_3)_2Cl_2$, 10 mg (0.053 mmol, 87 mol%) CuI, and 92 mg (0.15 mmol,

2.5 eq.) **2-18** dissolved in 10 mL 1:1 (v/v) deoxygenated THF:NEt_3. The mixture was stirred 41 h at 60 °C. The resulting powder was purified by flash chromatography on silica gel (9:11 DCM:hexanes, 0.2% Et_3N), then recrystallized giving 89 mg yellow powder with green fluorescence. Successive recrystallizations from DCM/hexanes, titruation with hexanes, and recrystallization from $CHCl_3$/MeOH carried out to remove impurity giving excessive integration in ^{1}H NMR alkyl region 1.26-1.19 and 0.81 ppm (likely residual hexanes). Recrystallization from DCM/decane removed species giving excess integration, giving 7 mg in 6% final yield. Melting point 75.8–76.4 °C.

^{1}H NMR (500 MHz, $CDCl_3$) δ: 8.03 (s, 2H), 7.17 (d, J = 8.7 Hz, 4H), 6.46 (d, J = 9.3 Hz, 4H), 5.39 (s, 4H), 3.21 (t, J = 7.6 Hz, 8H), 1.52 (broad s, 8H), 1.26-1.19 (m, 120H), 0.81 (t, J = 7.4 Hz, 12H)

^{19}F NMR (282 MHz, $CDCl_3$) δ: -141.4 (dt, J1 = 22 Hz, J2 = 8 Hz), -152.2 (t, J = 21 Hz), -161.3 (td, J1 = 18 Hz, J2 = 7 Hz)

^{13}C NMR (125 MHz, $CDCl_3$) δ: 164.6, 148.4, 145.8 (d, J = 258 Hz), 141.8 (d, J = 255 Hz), 137.6 (d, J = 251 Hz), 136.0, 132.9, 132.6, 123.2, 111.0, 109.1, 107.8, 99.2, 85.5, 54.1, 51.0, 31.9, 29.7, 29.6, 29.5, 29.4, 27.2, 27.1, 22.7, 14.1

HRMS (ESI QTOF) calculated for $C_{110}H_{162}F_{10}N_2O_4$ $[M+2H]^{+2}$: 883.6266, found $[M+2H]^{+2}$: 884.1255

A-1F0

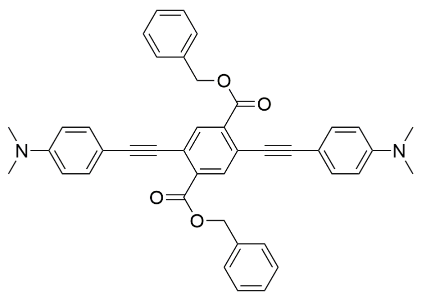

This reaction followed the general procedure for **A-R** with 46 mg (0.077 mmol, 1.0 eq.) **3-F0**, 2 mg (0.002 mmol, 2 mol%) $Pd(PPh_3)_2Cl_2$, 1 mg (0.005 mmol, 7 mol%) CuI, and 31 mg (0.21 mmol, 2.8 eq.) 4-ethynyl-N,N-dimethylaniline dissolved in 10 mL 1:1 (v/v) deoxygenated THF:NEt_3. The mixture was stirred 72 h at 60 °C. The resulting powder was purified by flash chromatography on silica gel (7:3 DCM:hexanes, then gradient to pure DCM), then recrystallized giving 23 mg orange powder with yellow fluorescence in 47% yield. Melting point 200.8-202.6 °C (dec). Red prisms with orange fluorescence for single crystal X-ray diffraction were grown from slow evaporation of a solution of dichloromethane with layered hexanes.

^{1}H NMR (500 MHz, $CDCl_3$) δ: 8.19 (s, 2H), 7.52 (d, J = 8.2 Hz, 4H), 7.38 (m, 10H), 6.64 (d, J = 8.3 Hz, 4H), 5.46 (s, 4H), 3.02 (s, 12H)

^{19}F NMR (470 MHz, $CDCl_3$) δ: No signal

^{13}C NMR (125 MHz, $CDCl_3$) δ: 165.4, 150.4, 135.8, 135.7, 133.3, 133.1, 128.6, 128.4, 128.3, 122.9, 111.7, 109.6, 98.6, 86.3, 67.3, 40.2

HRMS (ESI QTOF) calculated for $C_{42}H_{36}N_2O_4$ [M+H]: 633.2753, found [M+H]: 633.2751

3. NMR Data

^{1}H NMR of **1-18**

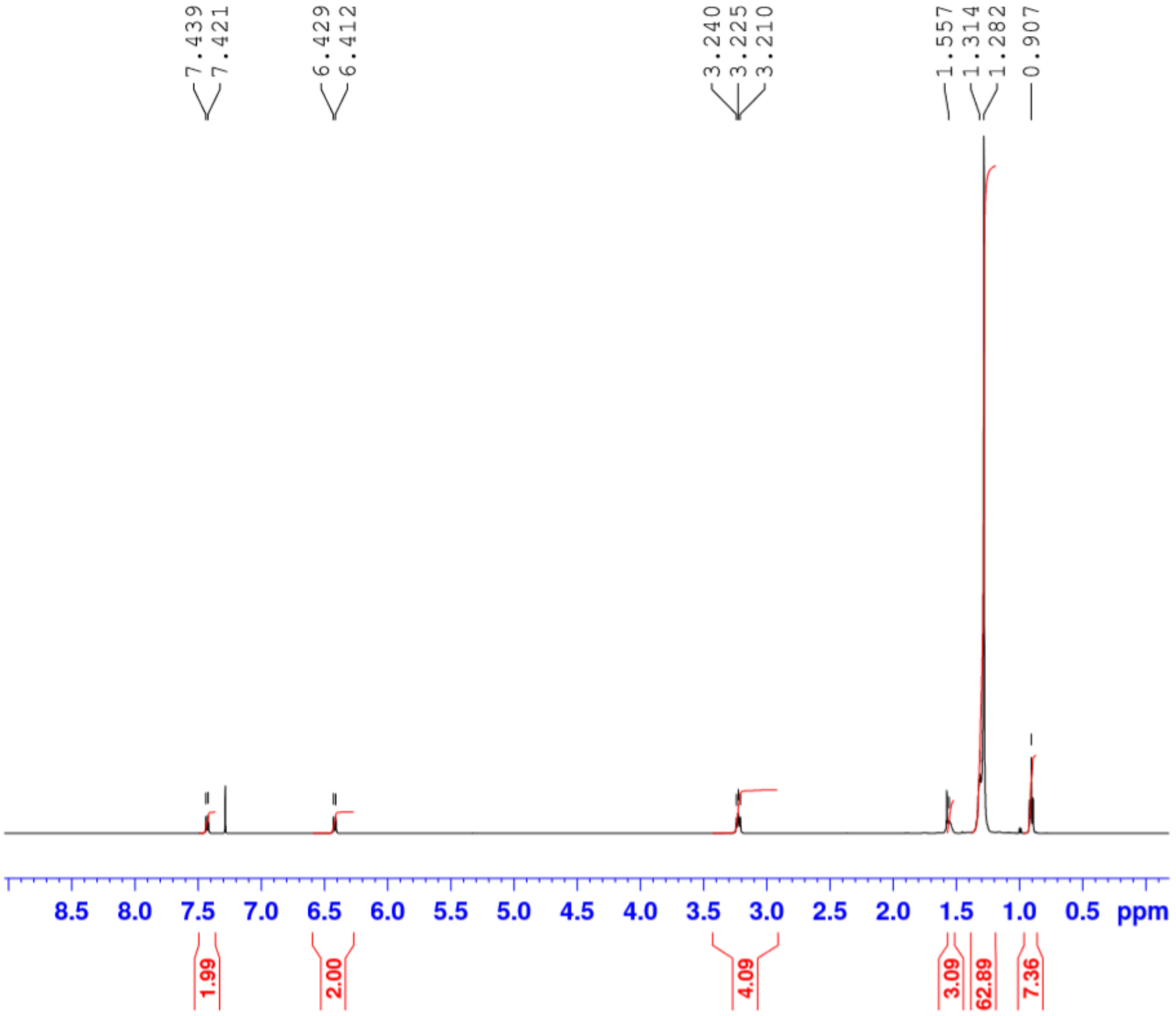

^{13}C NMR of **1-18**

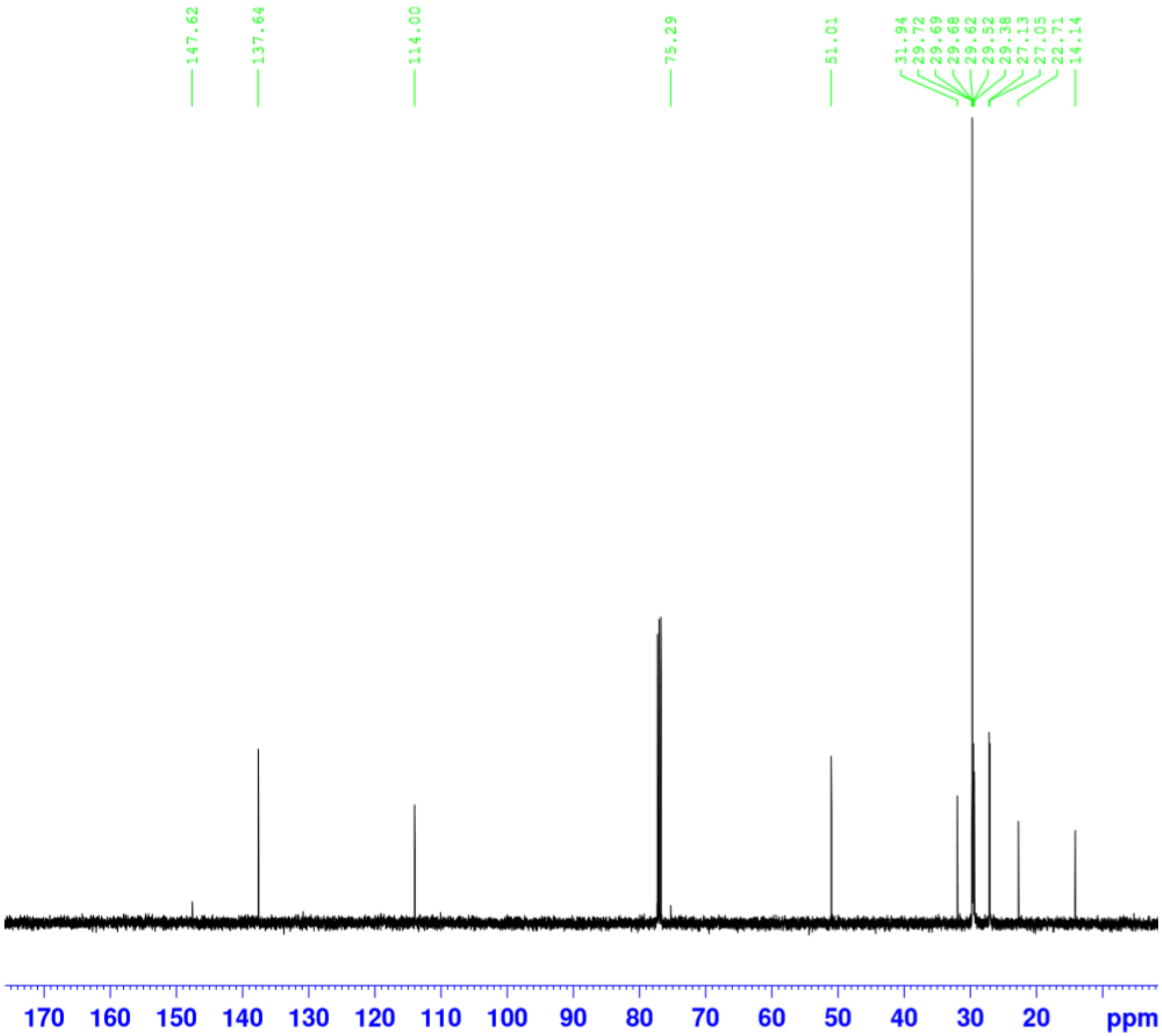

^{1}H NMR of **2-18**

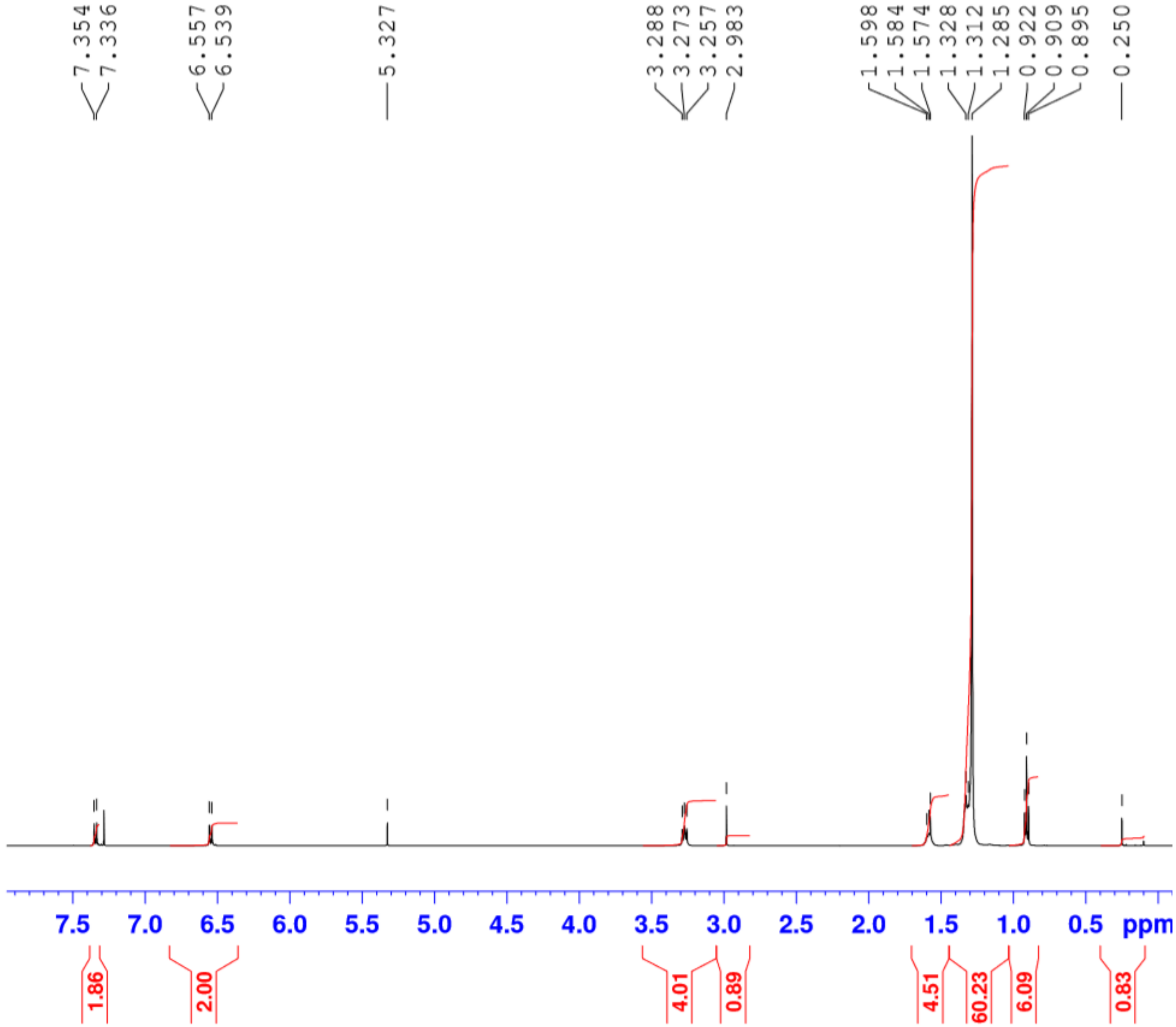
7.354
7.336
6.557
6.539
5.327
3.288
3.273
3.257
2.983
1.598
1.584
1.574
1.328
1.312
1.285
0.922
0.909
0.895
0.250
7.5
7.0
6.5
6.0
5.5
5.0
4.5
4.0
3.5
3.0
2.5
2.0
1.5
1.0
0.5
ppm
1.86
2.00
4.01
0.89
4.51
60.23
6.09
0.83

^{13}C NMR of **2-18**

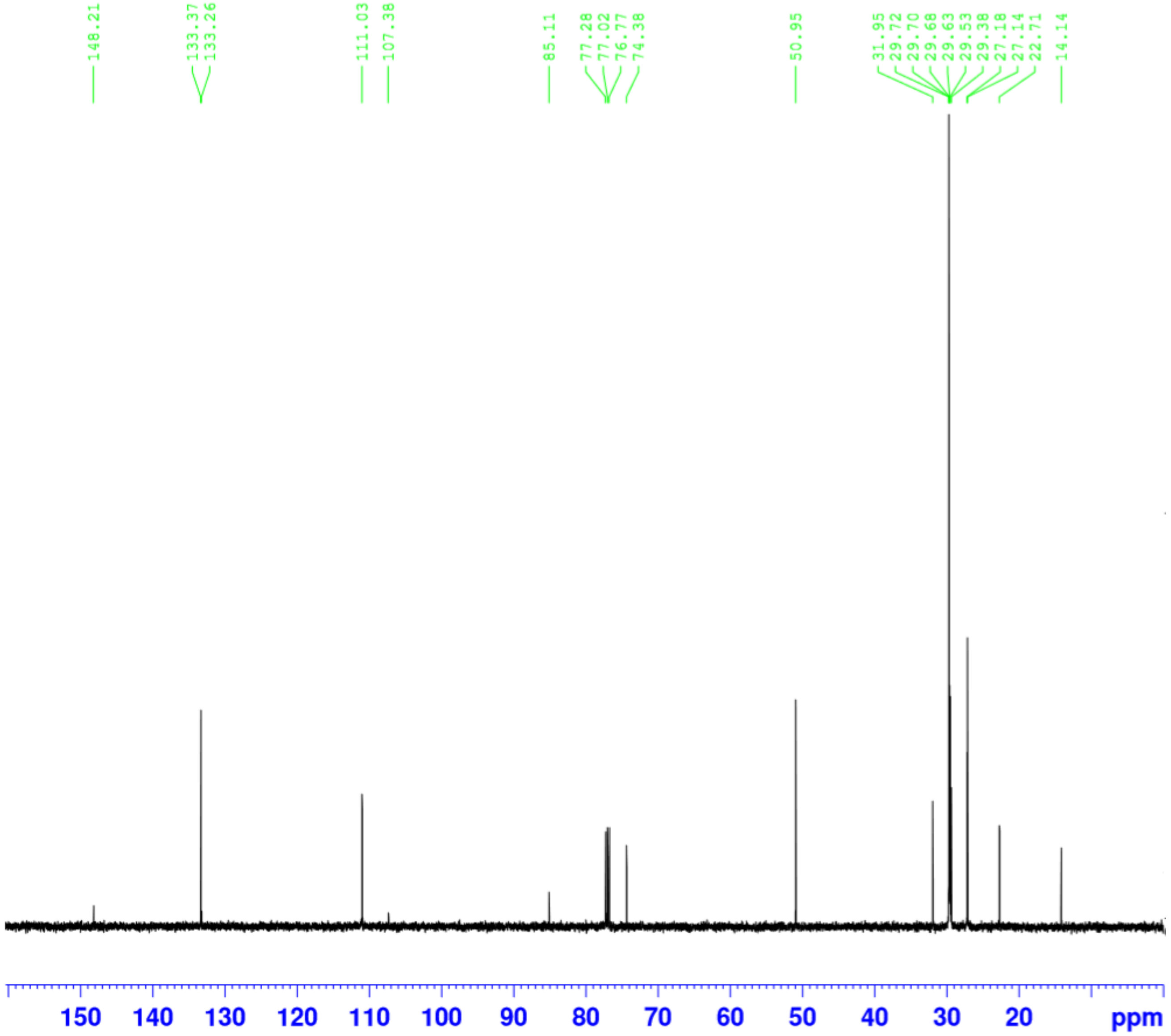

^{1}H NMR of **A-2**

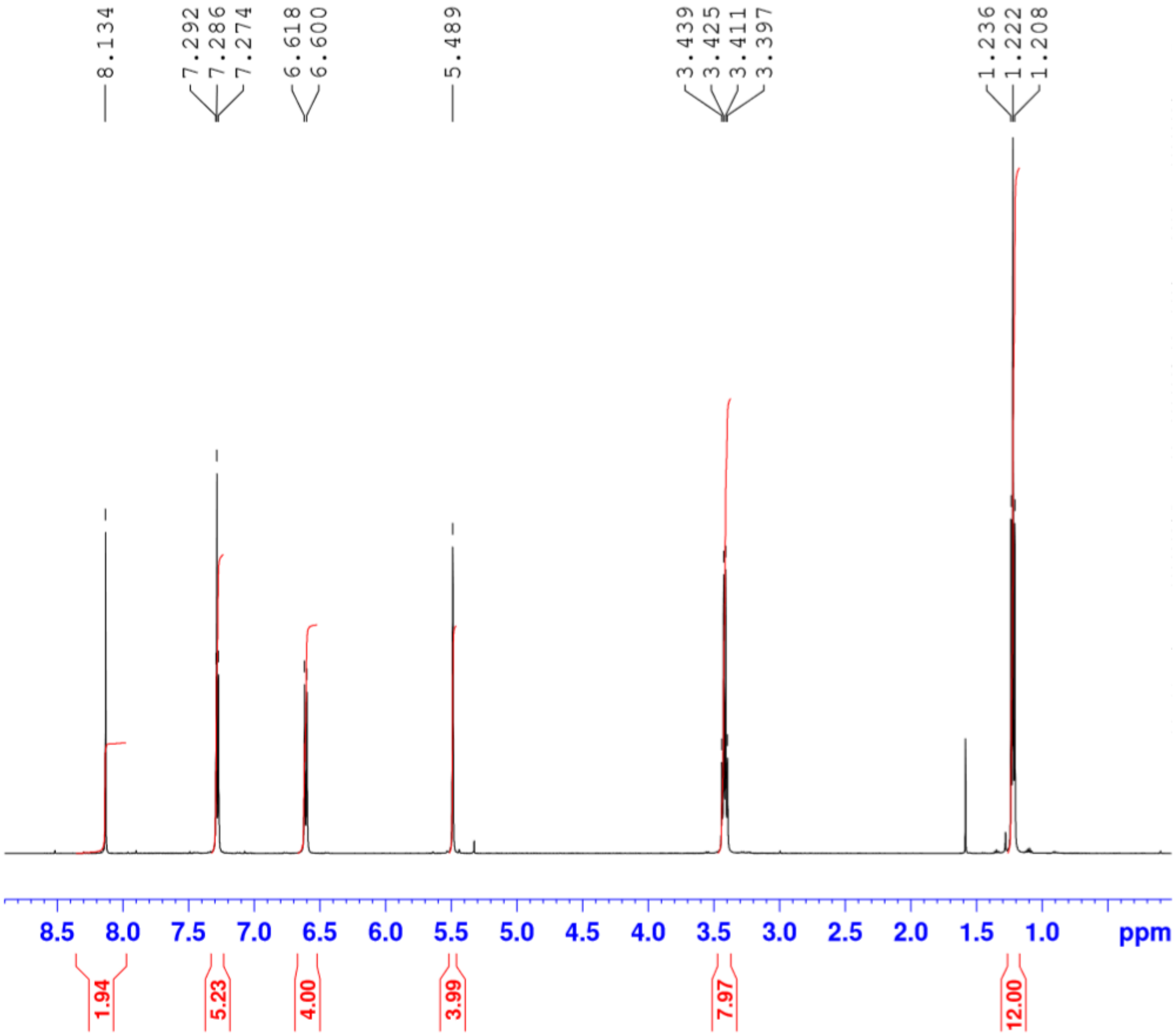
8.134
7.292
7.286
7.274
6.618
6.600
5.489
3.439
3.425
3.411
3.397
1.236
1.222
1.208
8.5
8.0
7.5
7.0
6.5
6.0
5.5
5.0
4.5
4.0
3.5
3.0
2.5
2.0
1.5
1.0
ppm
1.94
5.23
4.00
3.99
7.97
12.00

^{19}F NMR of **A-2**

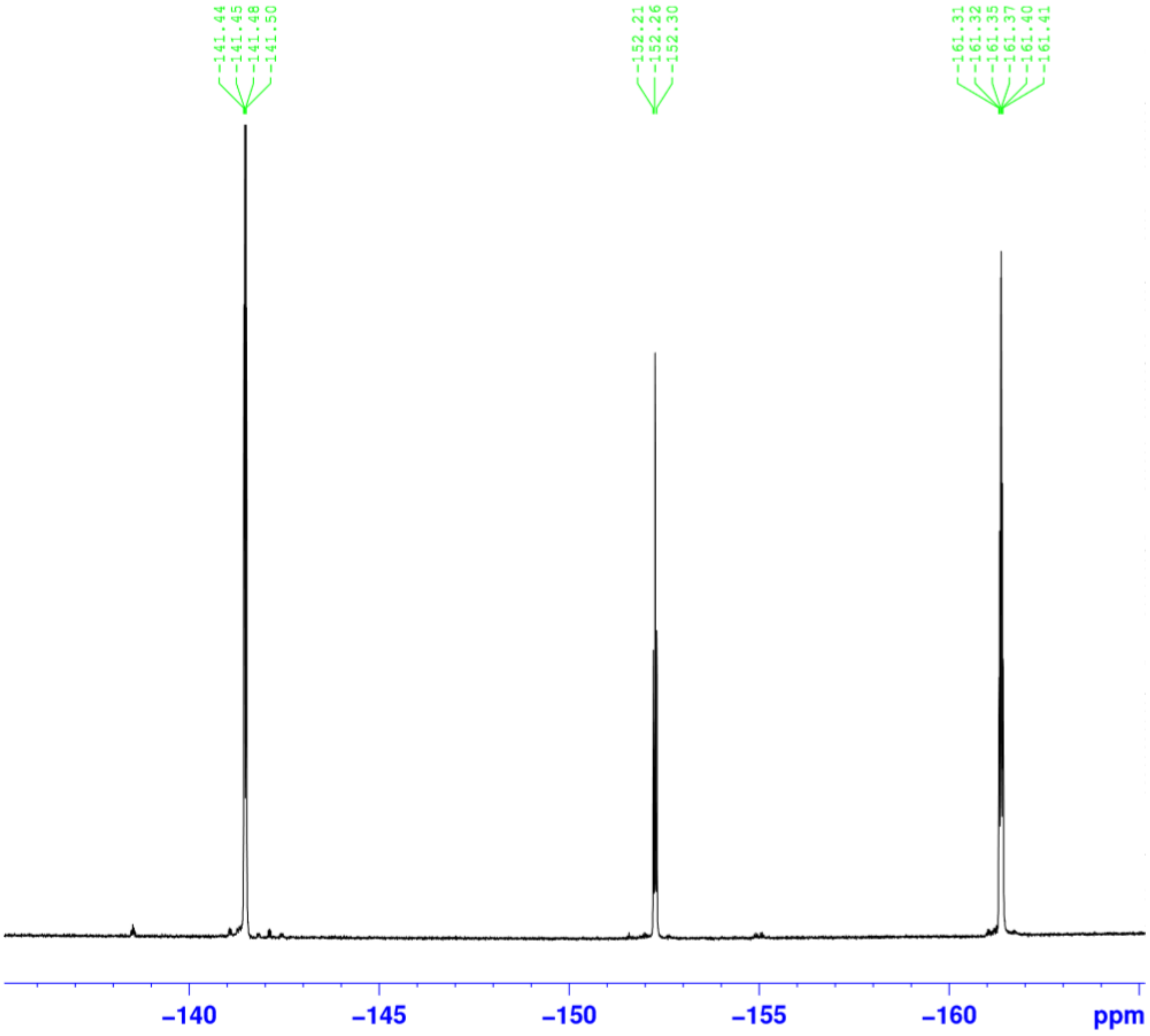
-141.44
-141.45
-141.48
-141.50
-152.21
-152.26
-152.30
-161.31
-161.32
-161.35
-161.37
-161.40
-161.41
-140
-145
-150
-155
-160
ppm

^{13}C NMR of **A-2**

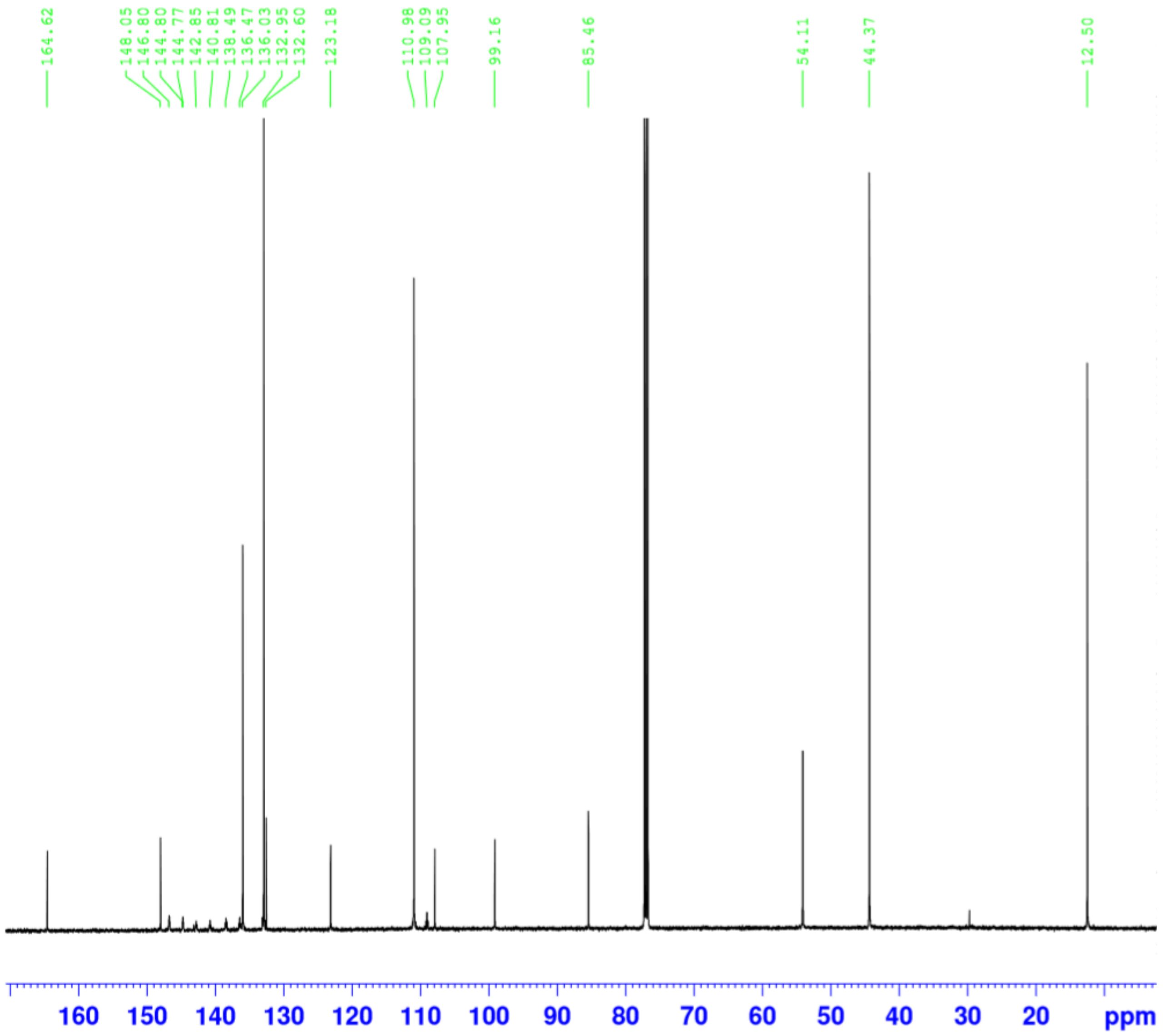
164.62
148.05
146.80
144.80
144.77
142.85
140.81
138.49
136.47
136.03
132.95
132.60
123.18
110.98
109.09
107.95
99.16
85.46
54.11
44.37
12.50
160
150
140
130
120
110
100
90
80
70
60
50
40
30
20
ppm

^{1}H NMR of **A-3**

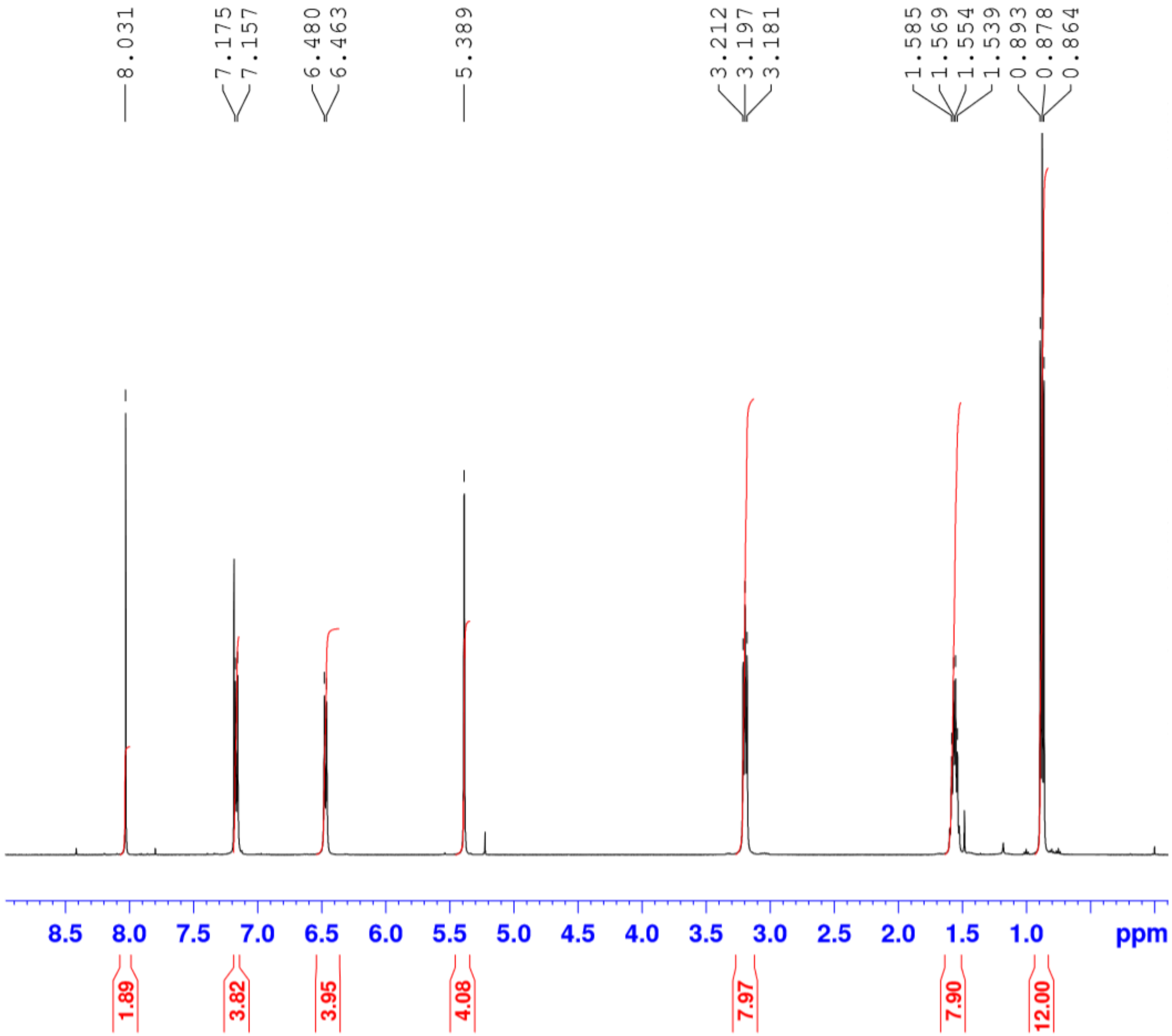
8.031
7.175
7.157
6.480
6.463
5.389
3.212
3.197
3.181
1.585
1.569
1.554
1.539
0.893
0.878
0.864
8.5
8.0
7.5
7.0
6.5
6.0
5.5
5.0
4.5
4.0
3.5
3.0
2.5
2.0
1.5
1.0
ppm
1.89
3.82
3.95
4.08
7.97
7.90
12.00

^{19}F NMR of **A-3**

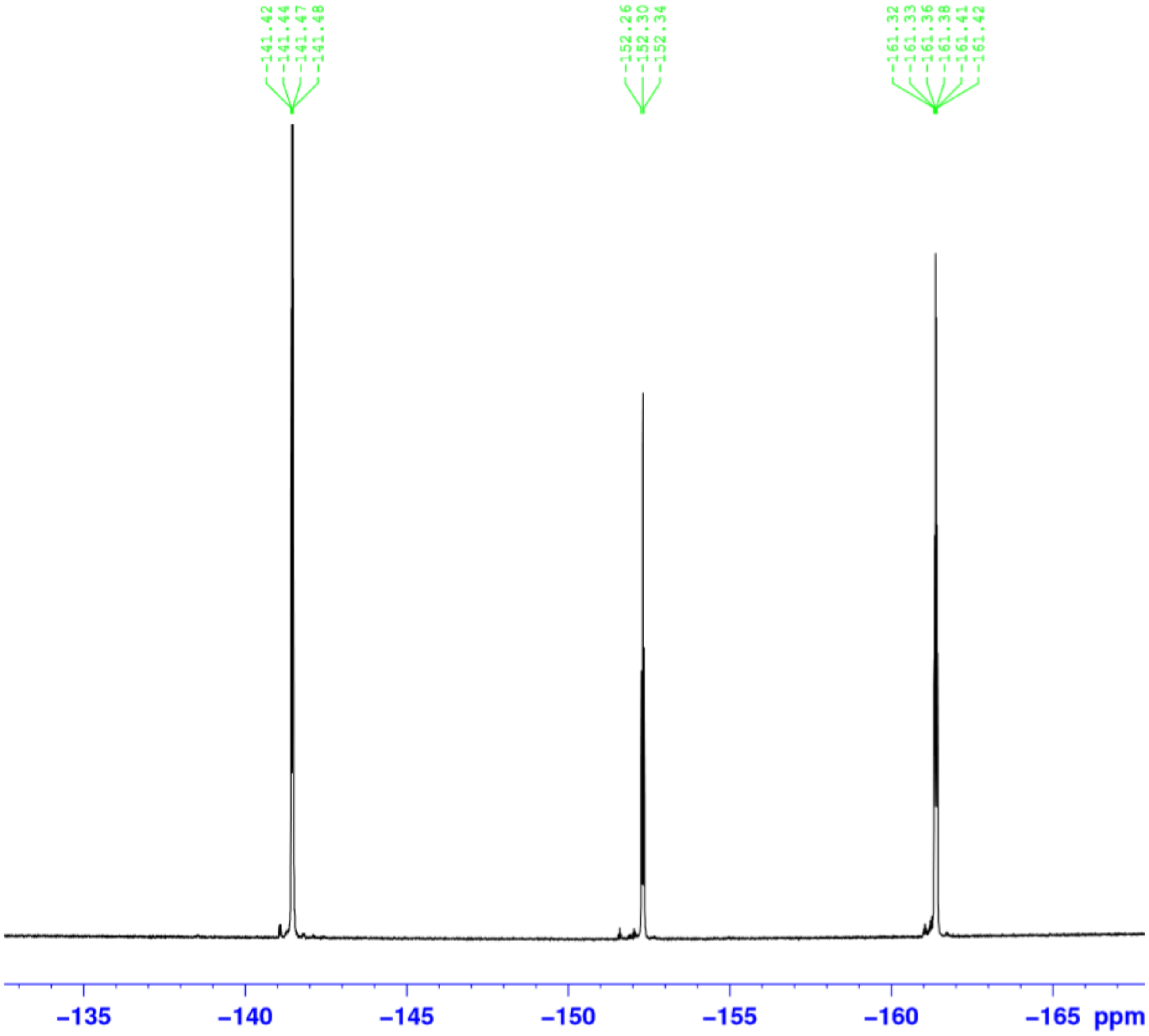
-141.42
-141.44
-141.47
-141.48
-152.26
-152.30
-152.34
-161.32
-161.33
-161.36
-161.38
-161.41
-161.42
-135
-140
-145
-150
-155
-160
-165
ppm

^{13}C NMR of **A-3**

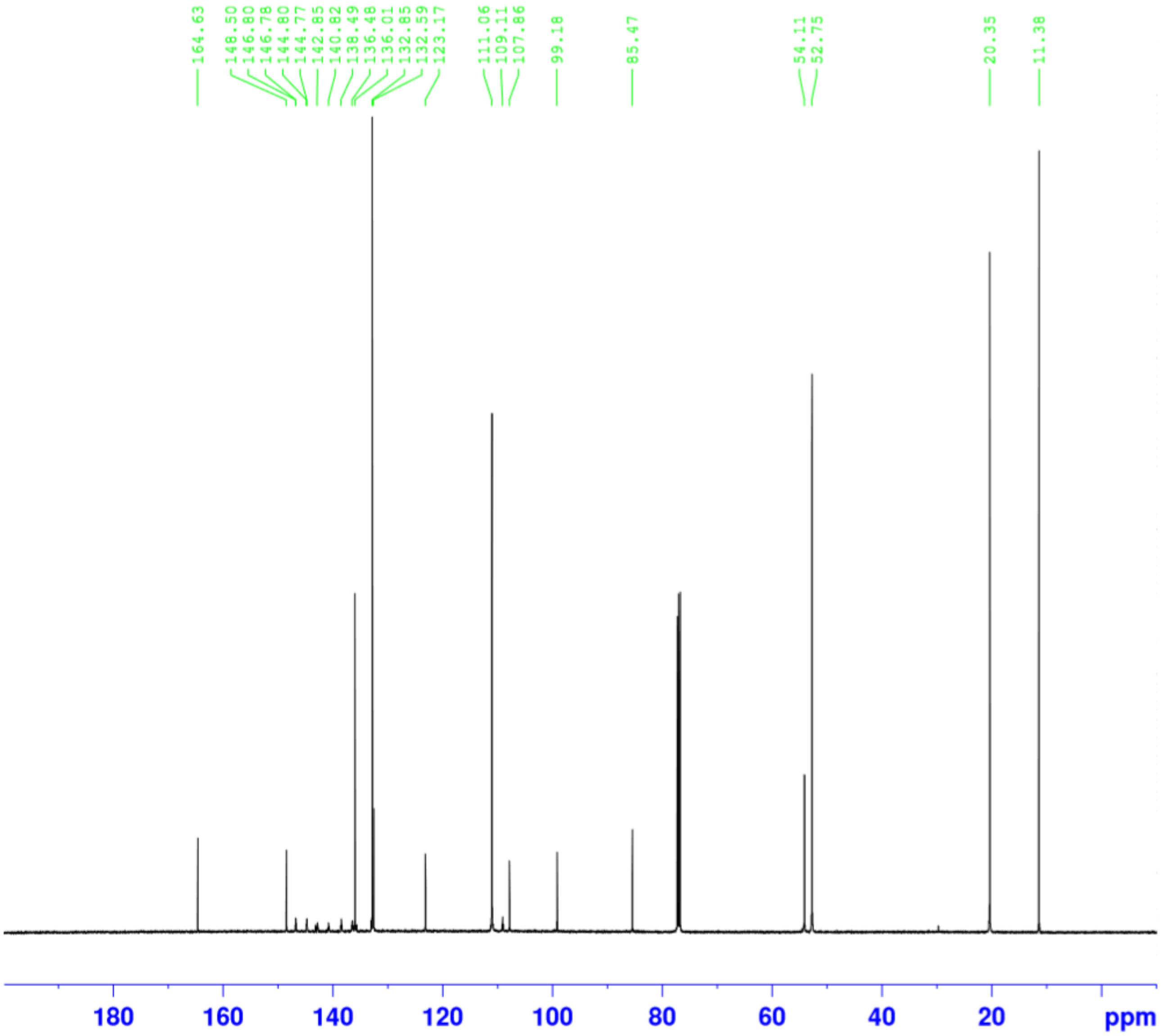
164.63
148.50
146.80
146.78
144.80
144.77
142.85
140.82
138.49
136.48
136.01
132.85
132.59
123.17
111.06
109.11
107.86
99.18
85.47
54.11
52.75
20.35
11.38
180
160
140
120
100
80
60
40
20
ppm

^{1}H NMR of **A-4**

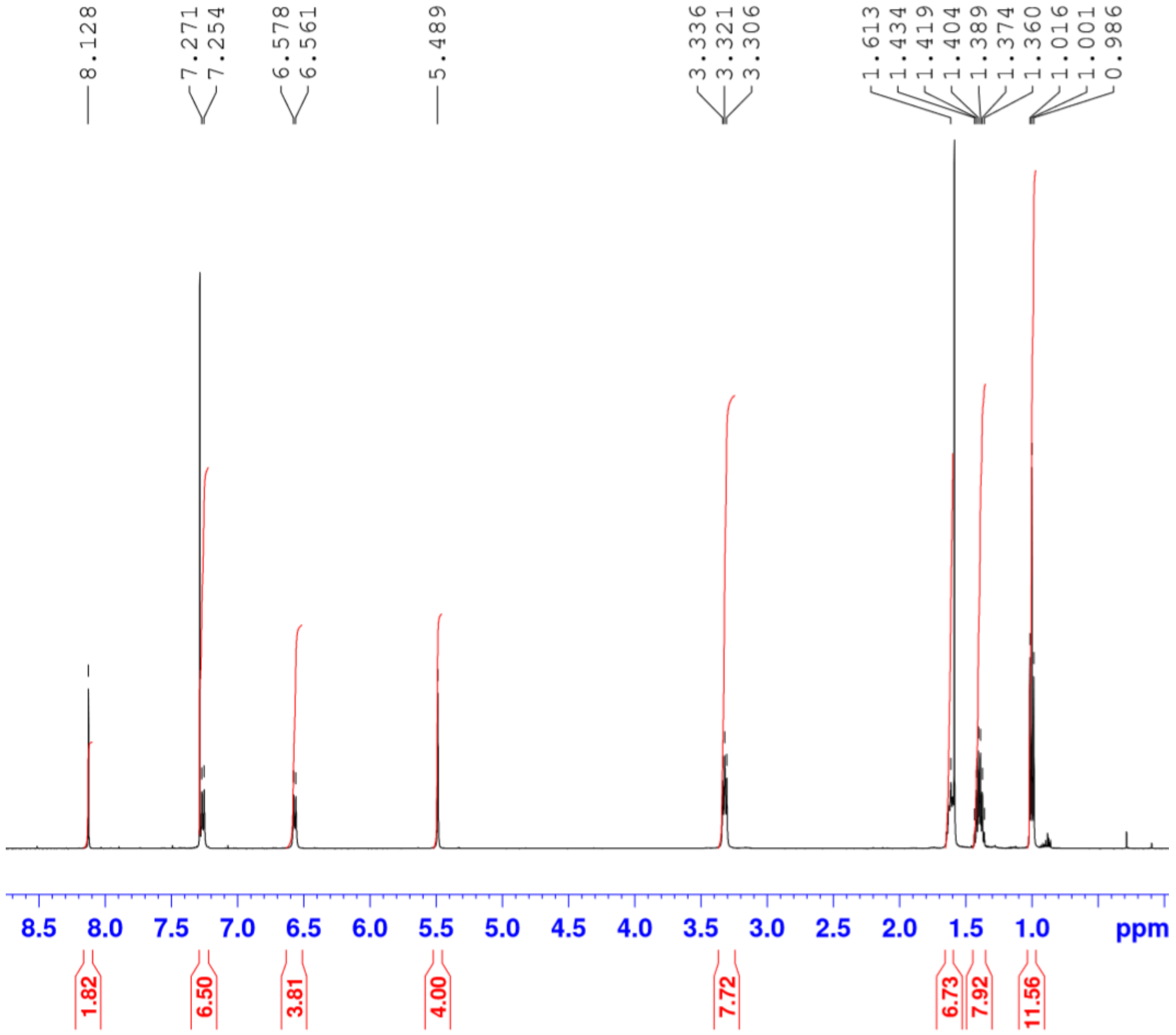
8.128
7.271
7.254
6.578
6.561
5.489
3.336
3.321
3.306
1.613
1.434
1.419
1.404
1.389
1.374
1.360
1.016
1.001
0.986
8.5 8.0 7.5 7.0 6.5 6.0 5.5 5.0 4.5 4.0 3.5 3.0 2.5 2.0 1.5 1.0 ppm
1.82
6.50
3.81
4.00
7.72
6.73
7.92
11.56

^{19}F NMR of **A-4**

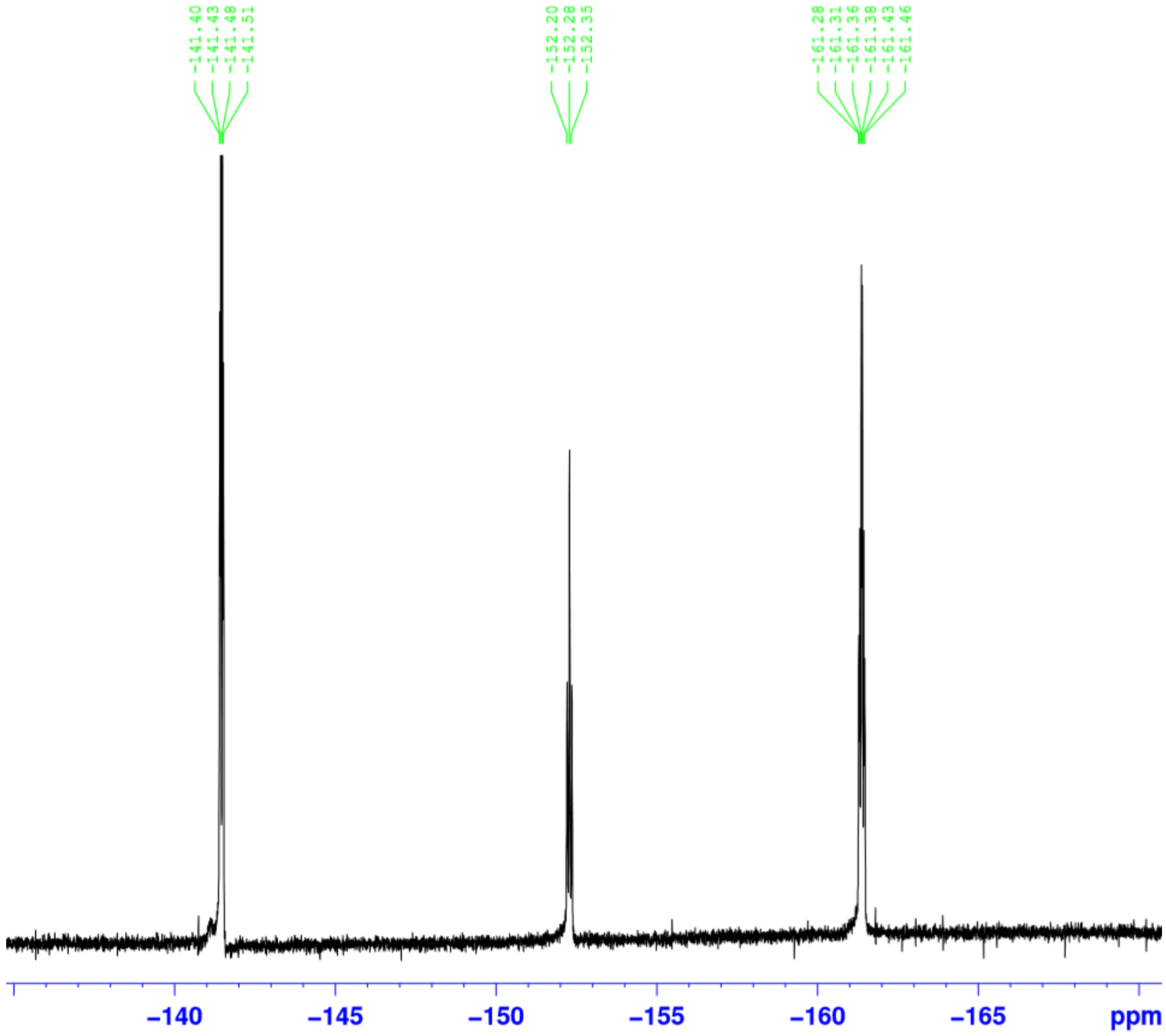
-141.40
-141.43
-141.48
-141.51
-152.20
-152.28
-152.35
-161.28
-161.31
-161.36
-161.38
-161.43
-161.46
-140
-145
-150
-155
-160
-165
ppm

^{13}C NMR of **A-4**

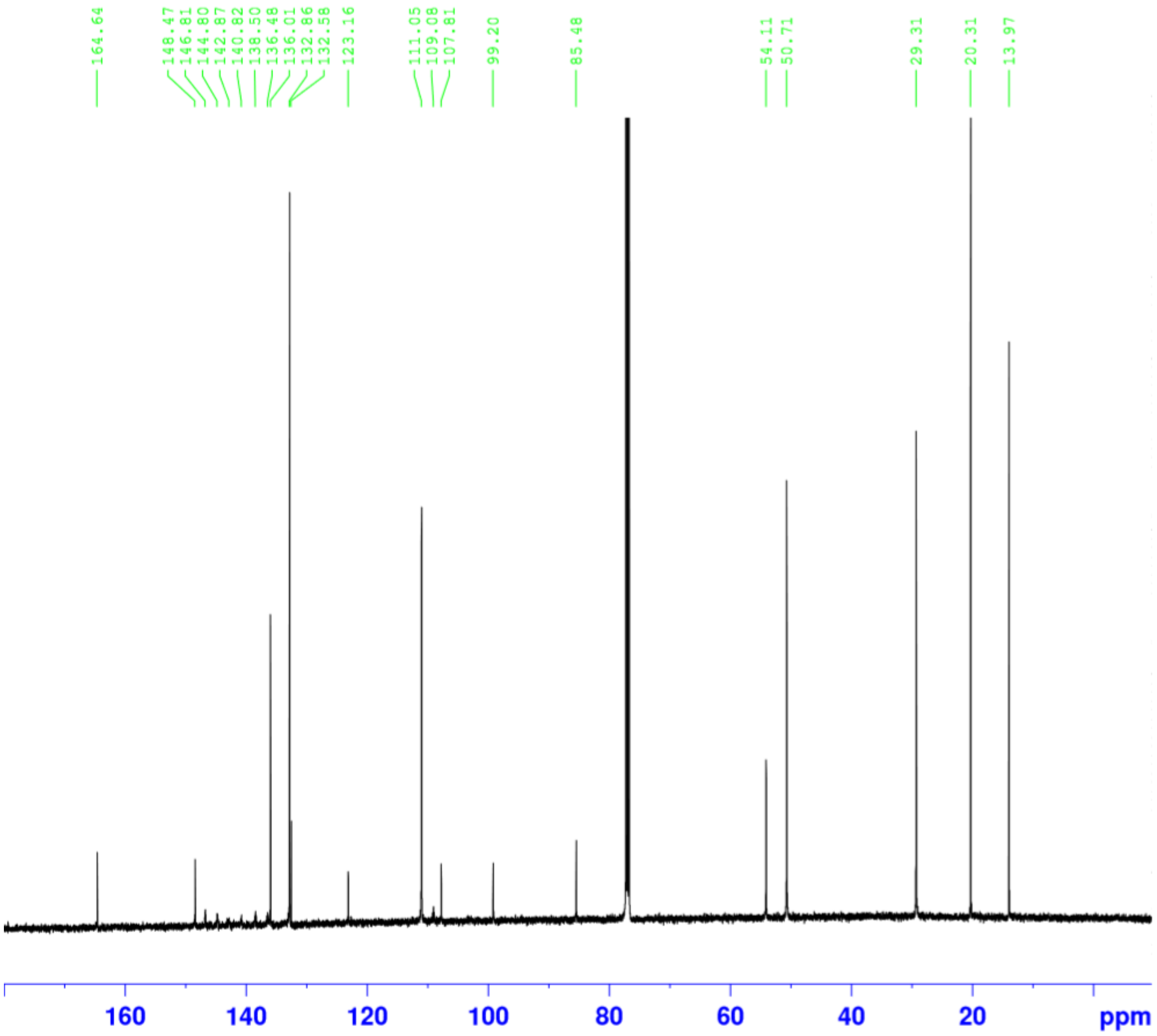
164.64
148.47
146.81
144.80
142.87
140.82
138.50
136.48
136.01
132.86
132.58
123.16
111.05
109.08
107.81
99.20
85.48
54.11
50.71
29.31
20.31
13.97
160
140
120
100
80
60
40
20
ppm

^{1}H NMR of **A-6**

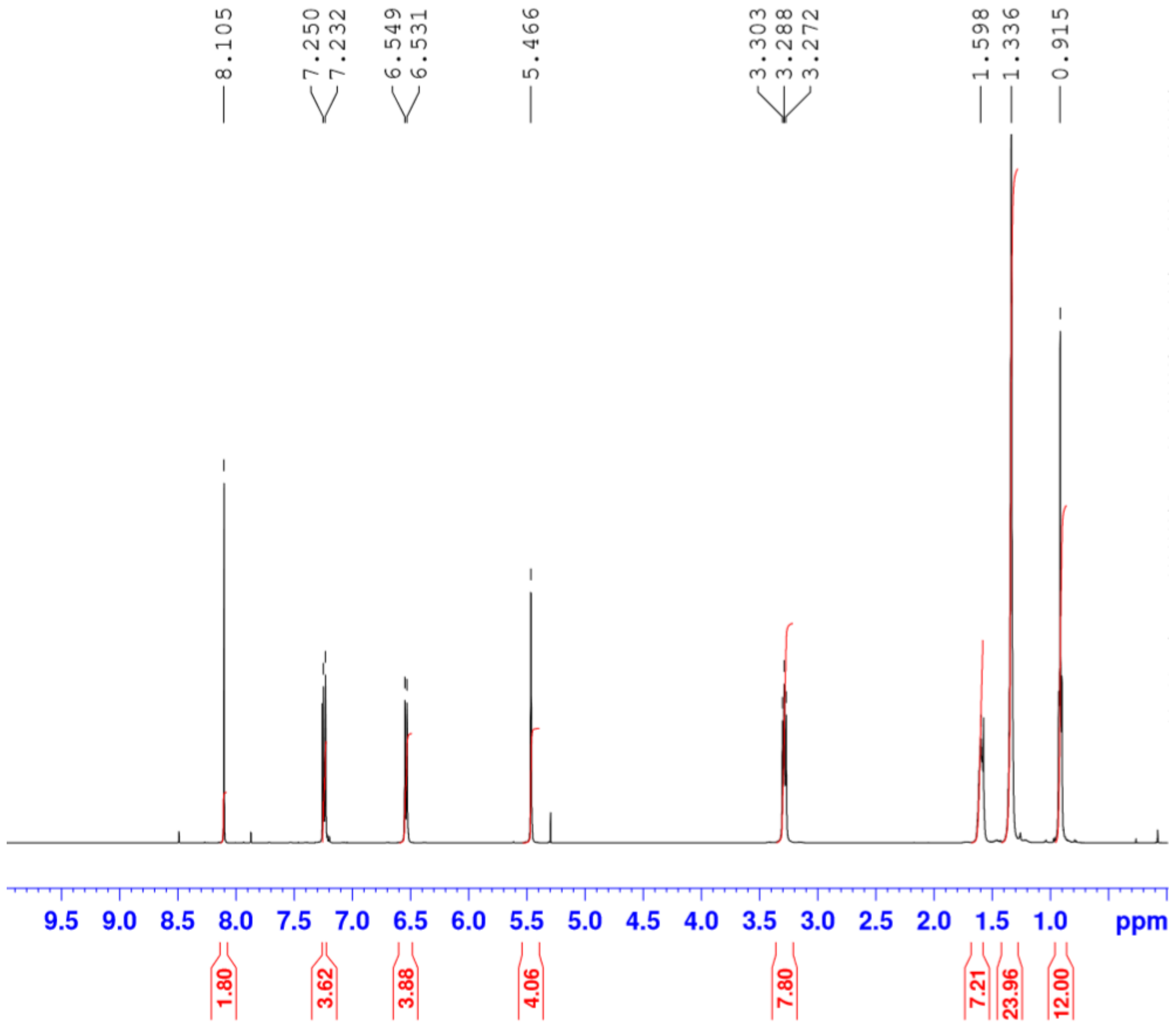
8.105
7.250
7.232
6.549
6.531
5.466
3.303
3.288
3.272
1.598
1.336
0.915
9.5 9.0 8.5 8.0 7.5 7.0 6.5 6.0 5.5 5.0 4.5 4.0 3.5 3.0 2.5 2.0 1.5 1.0 ppm
1.80
3.62
3.88
4.06
7.80
7.21
23.96
12.00

^{19}F NMR of **A-6**

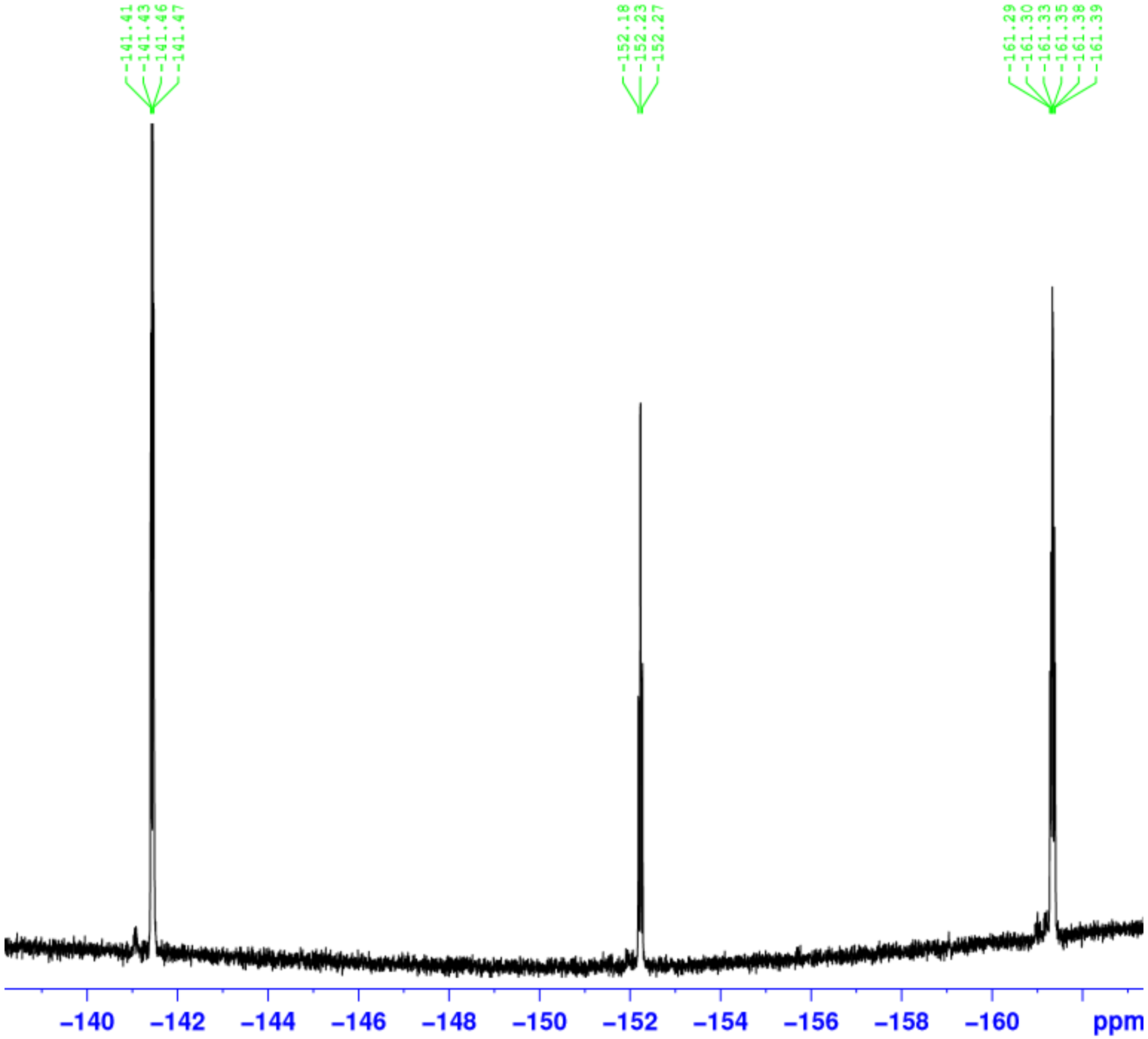
-141.41
-141.43
-141.46
-141.47
-152.18
-152.23
-152.27
-161.29
-161.30
-161.33
-161.35
-161.38
-161.39
-140
-142
-144
-146
-148
-150
-152
-154
-156
-158
-160
ppm

^{13}C NMR of **A-6**

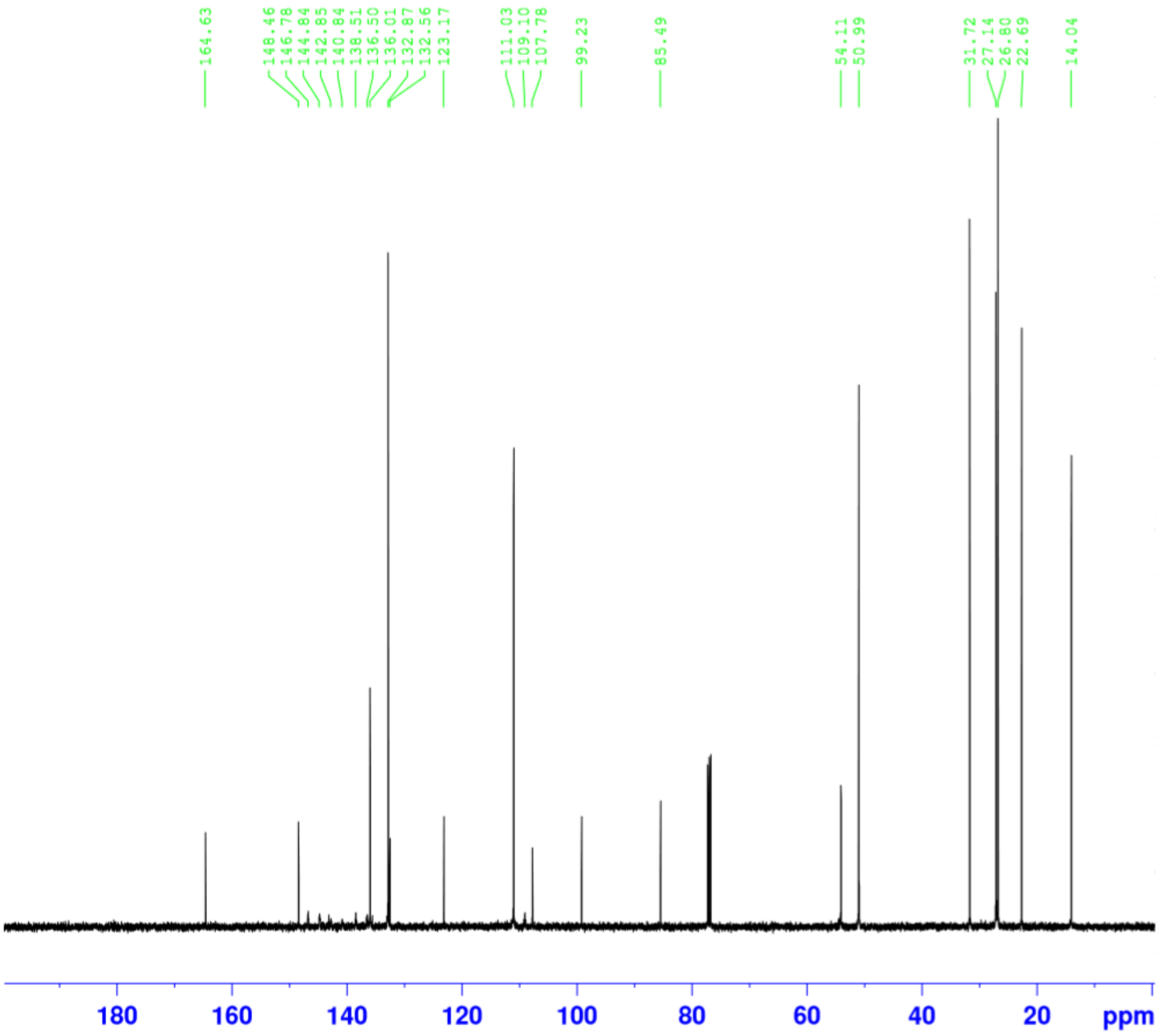
164.63
148.46
146.78
144.84
142.85
140.84
138.51
136.50
136.01
132.87
132.56
123.17
111.03
109.10
107.78
99.23
85.49
54.11
50.99
31.72
27.14
26.80
22.69
14.04
180
160
140
120
100
80
60
40
20
ppm

^{1}H NMR of **A-8**

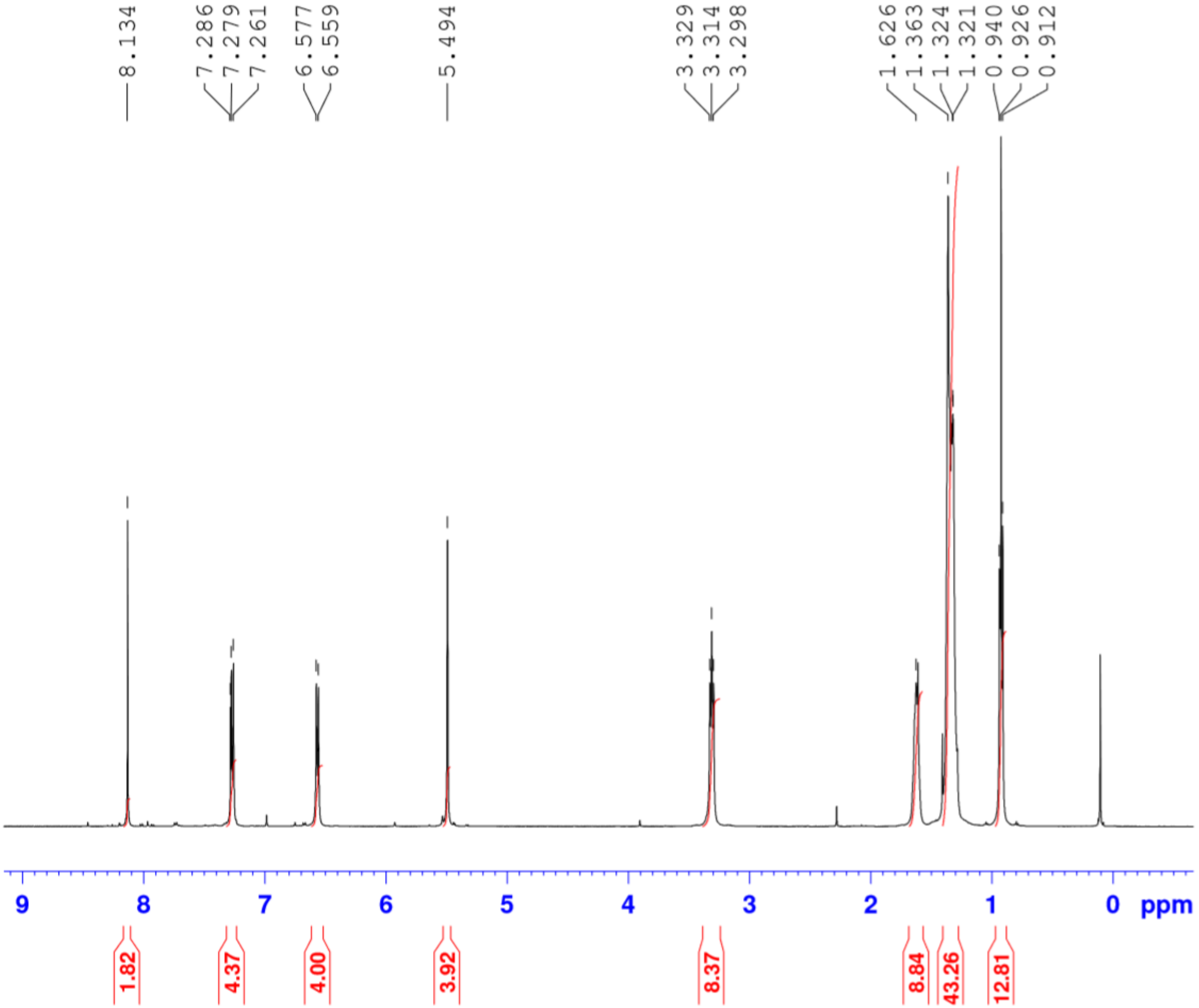
8.134
7.286
7.279
7.261
6.577
6.559
5.494
3.329
3.314
3.298
1.626
1.363
1.324
1.321
0.940
0.926
0.912
9
8
7
6
5
4
3
2
1
0
ppm
1.82
4.37
4.00
3.92
8.37
8.84
43.26
12.81

^{19}F NMR of **A-8**

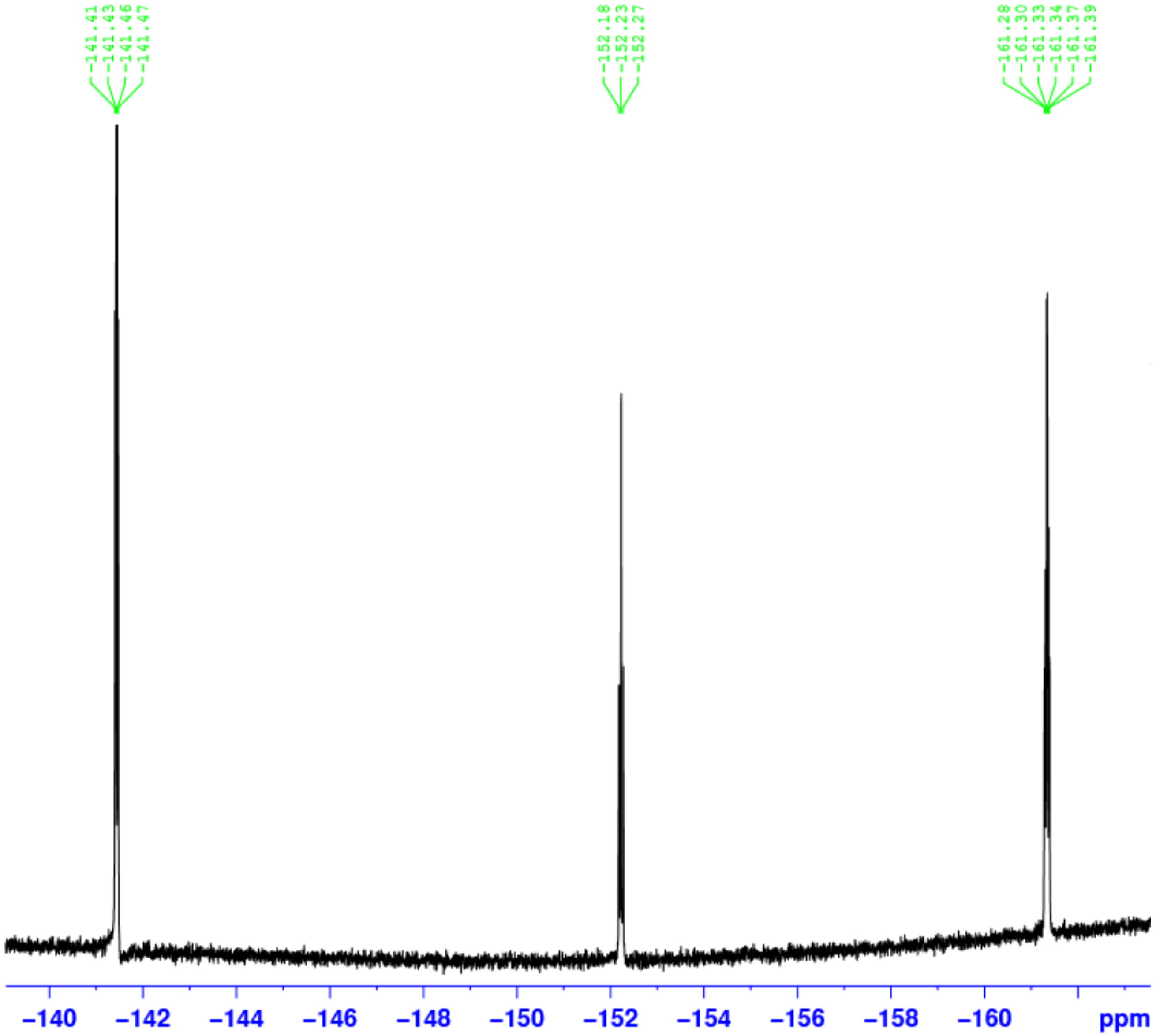
-141.41
-141.43
-141.46
-141.47
-152.18
-152.23
-152.27
-161.28
-161.30
-161.33
-161.34
-161.37
-161.39
−140
−142
−144
−146
−148
−150
−152
−154
−156
−158
−160
ppm

^{13}C NMR of **A-8**

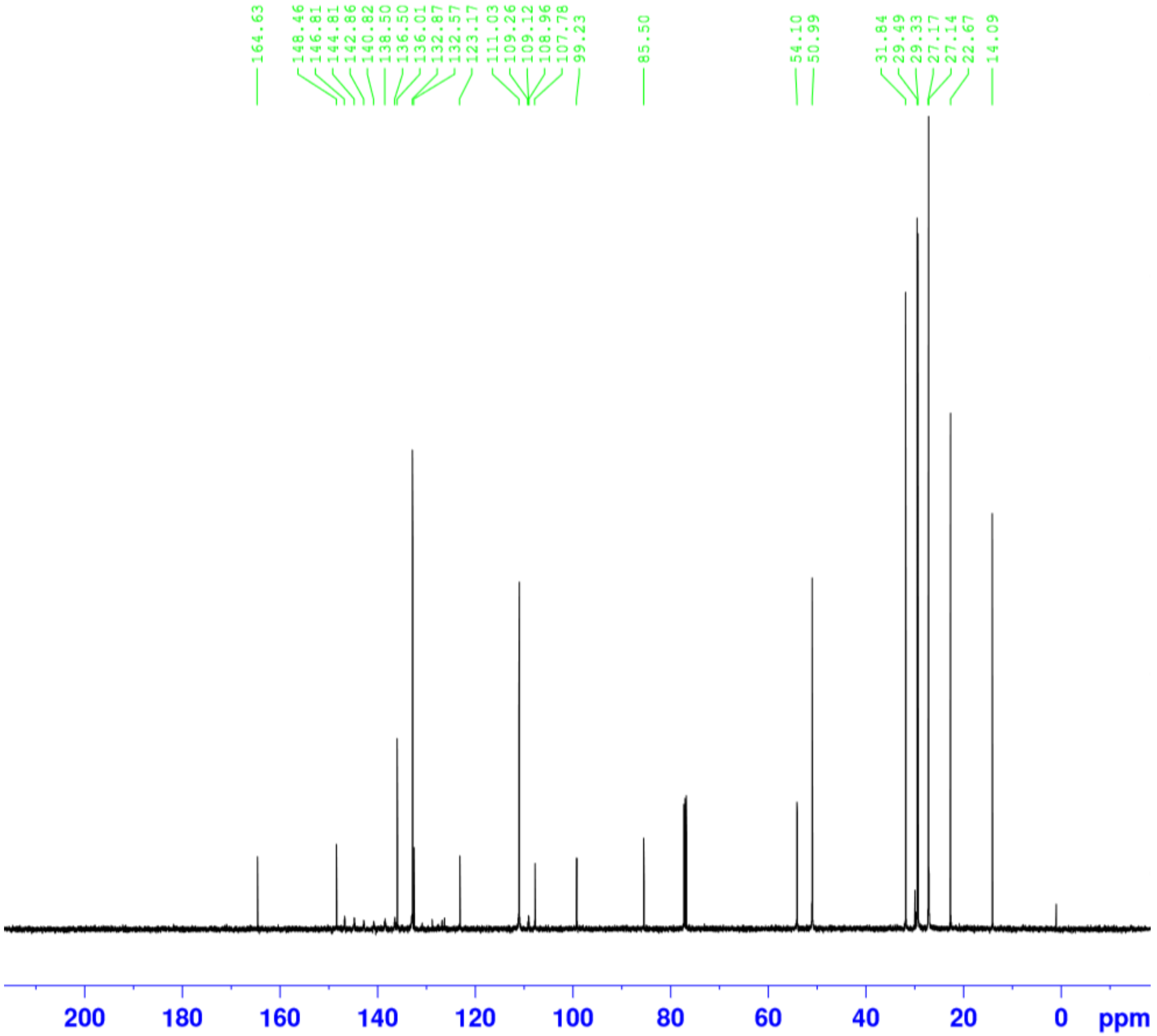
164.63
148.46
146.81
144.81
142.86
140.82
138.50
136.50
136.01
132.87
132.57
123.17
111.03
109.26
109.12
108.96
107.78
99.23
85.50
54.10
50.99
31.84
29.49
29.33
27.17
27.14
22.67
14.09
200
180
160
140
120
100
80
60
40
20
0
ppm

^{1}H NMR of **A-18**

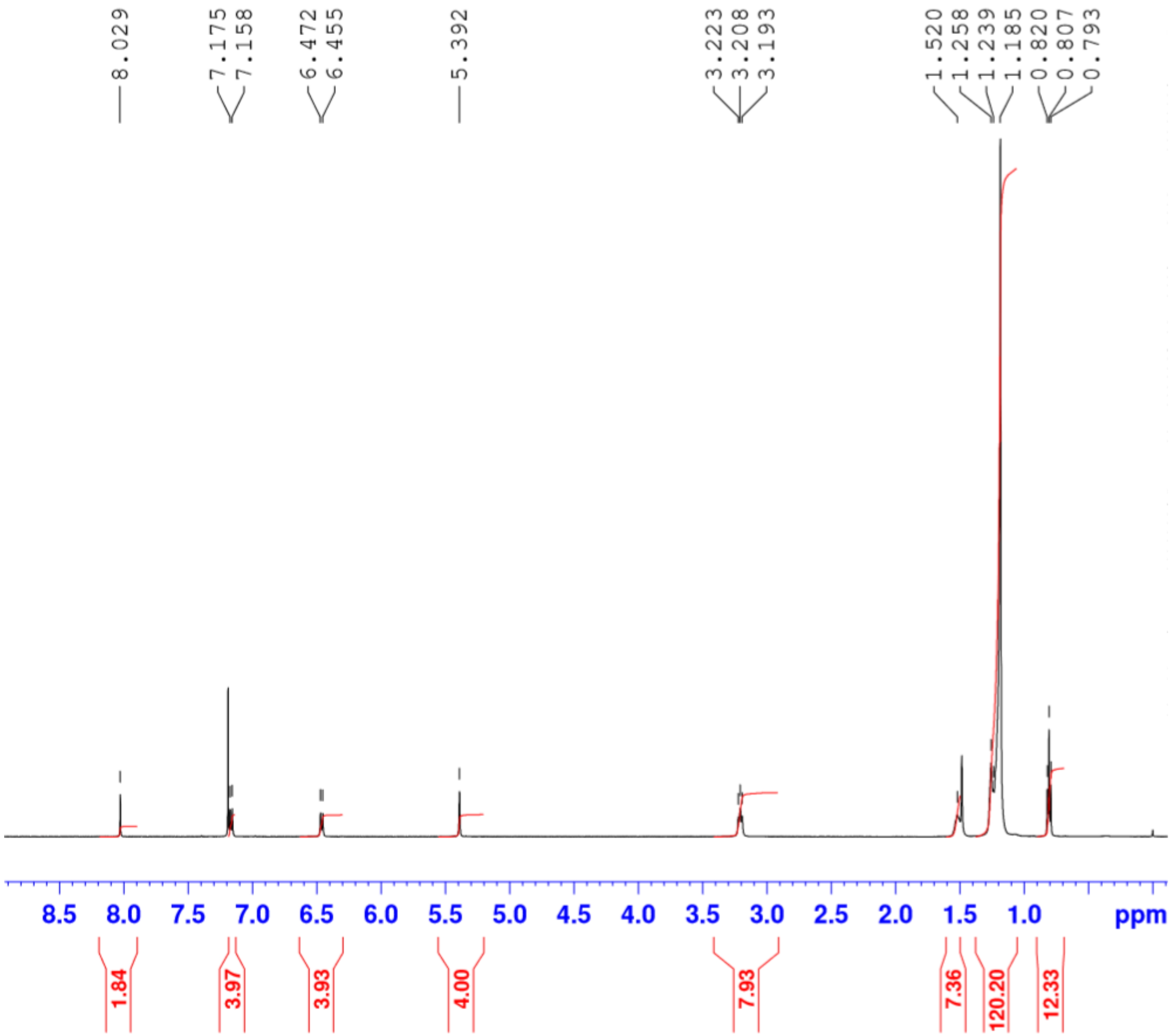
8.029
7.175
7.158
6.472
6.455
5.392
3.223
3.208
3.193
1.520
1.258
1.239
1.185
0.820
0.807
0.793
8.5 8.0 7.5 7.0 6.5 6.0 5.5 5.0 4.5 4.0 3.5 3.0 2.5 2.0 1.5 1.0 ppm
1.84
3.97
3.93
4.00
7.93
7.36
120.20
12.33

^{19}F NMR of **A-18**

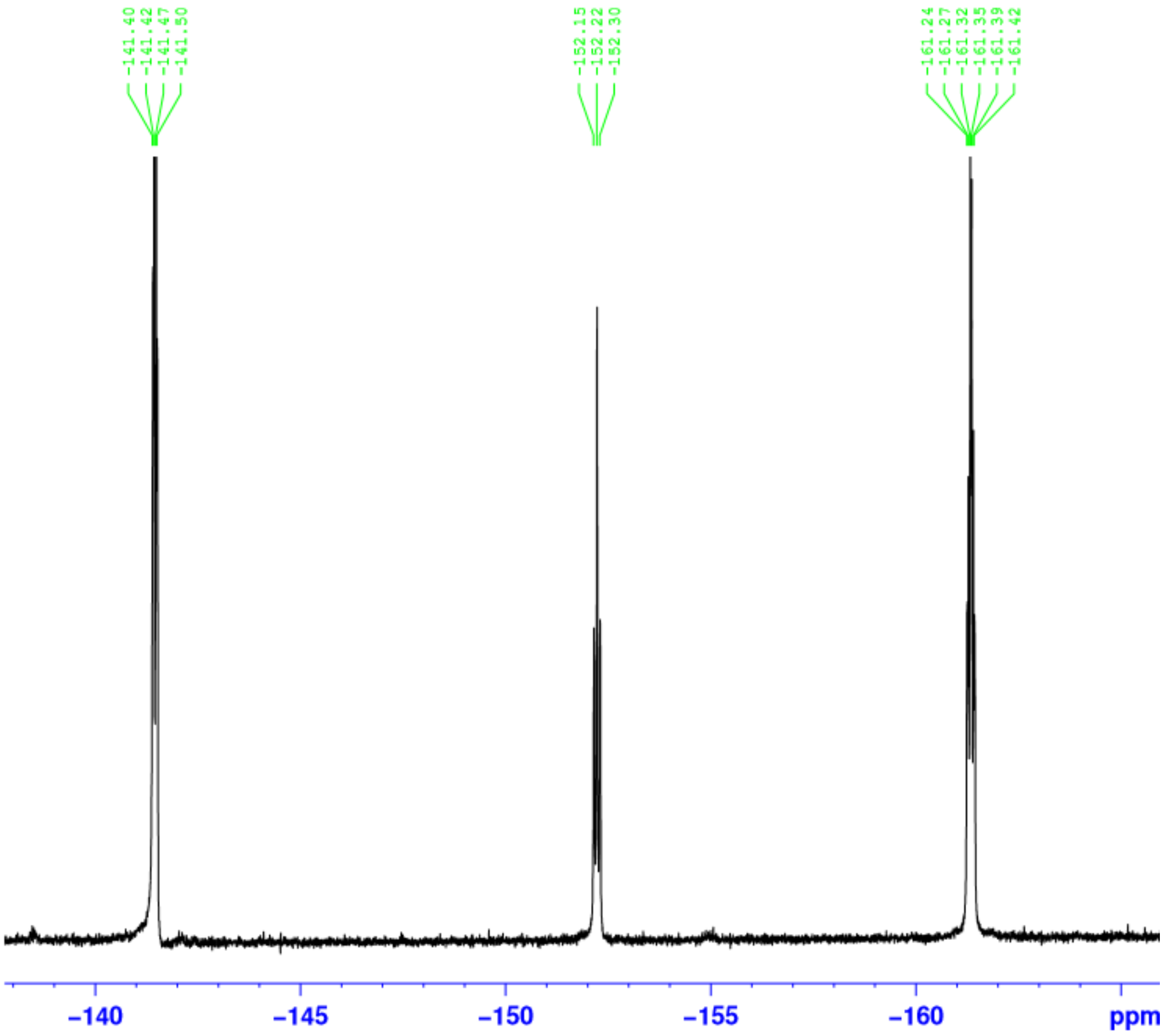

^{13}C NMR of **A-18**

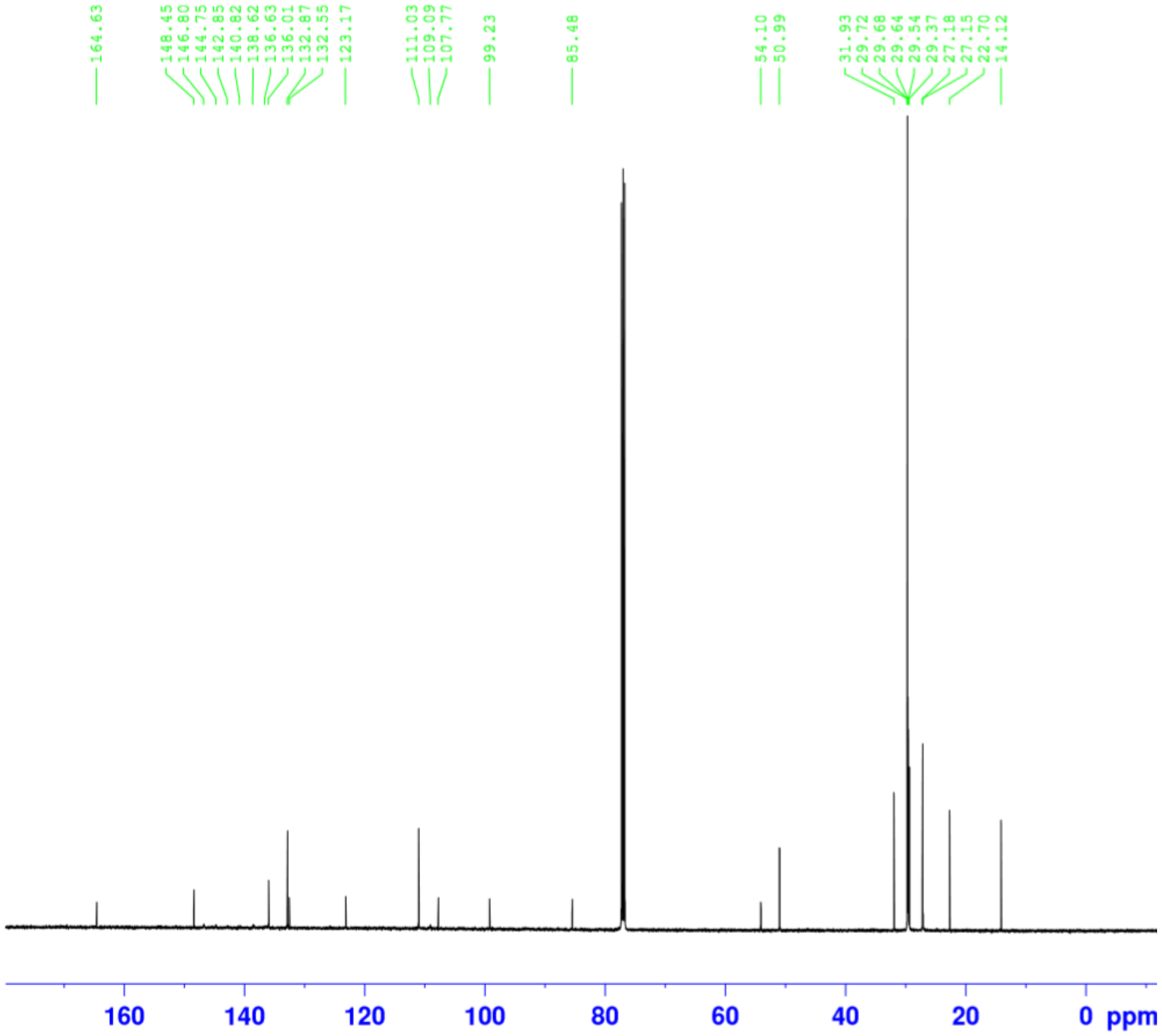

^{1}H NMR of **A-1F0**

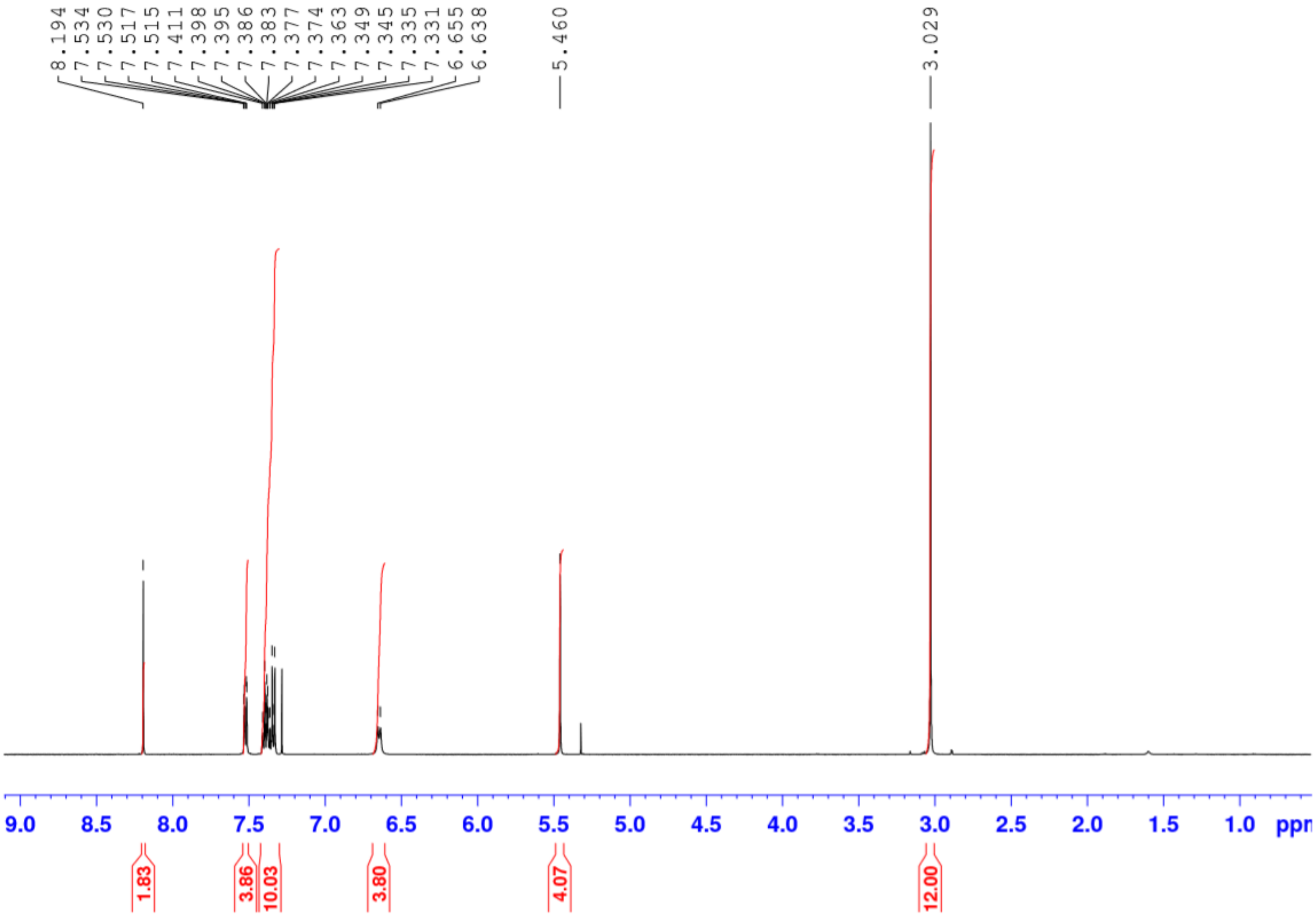

^{19}F NMR of **A-1F0**

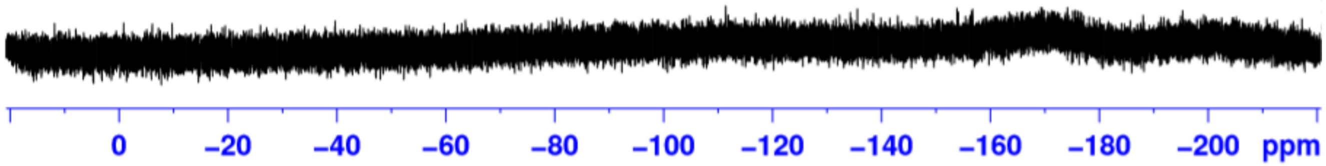

^{13}C NMR of **A-1F0**

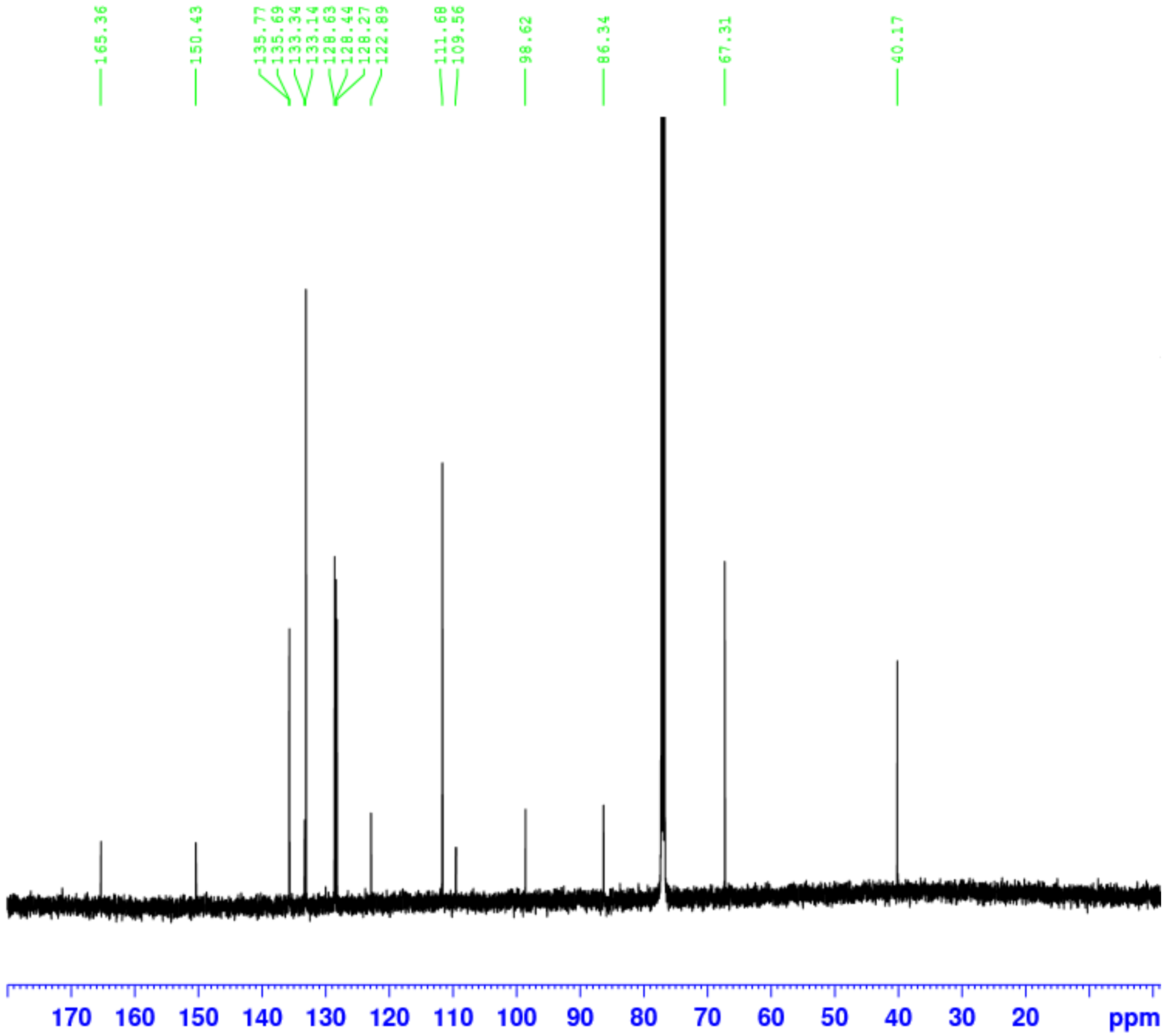

4. High Resolution Mass Spectrometry

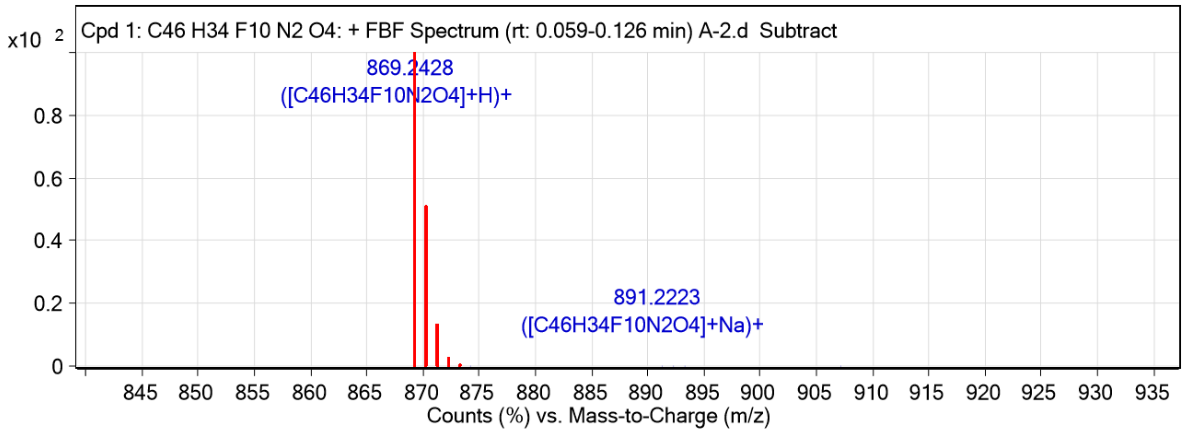

MS of **A-2**

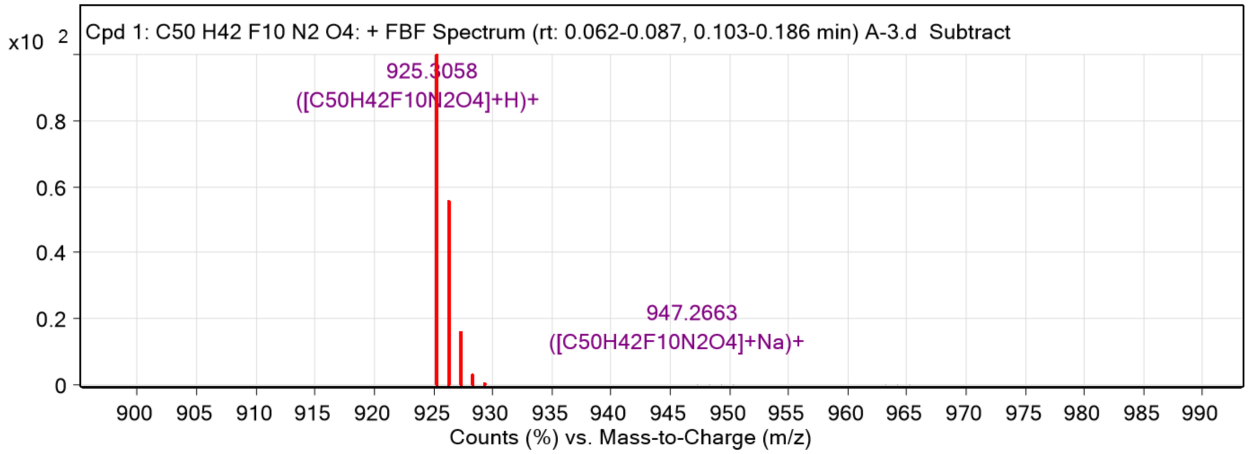

MS of **A-3**

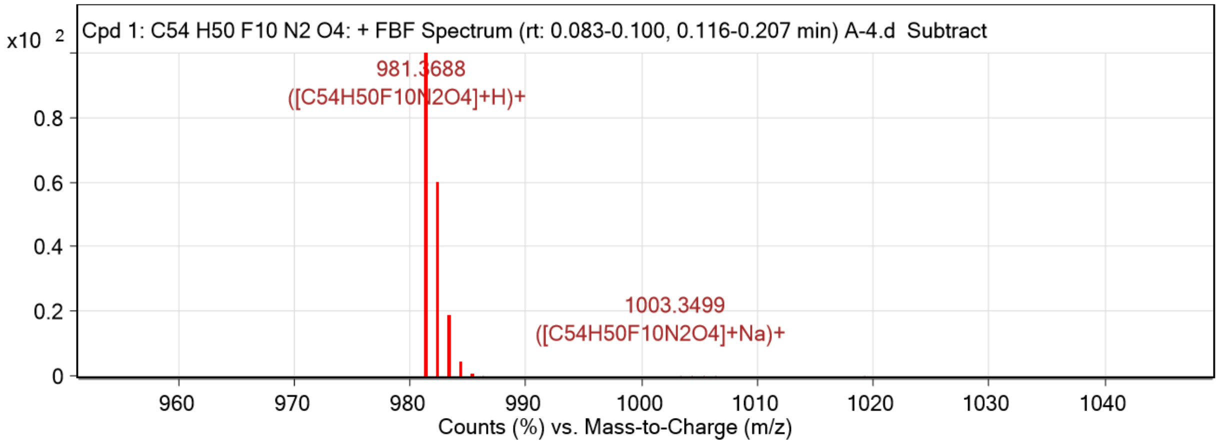

MS of **A-4**

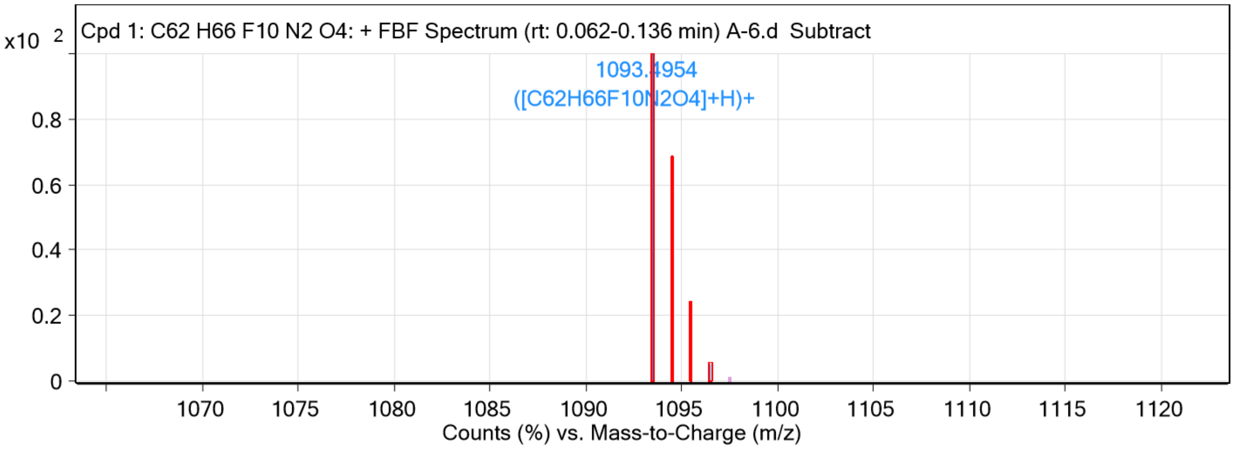

MS of **A-6**

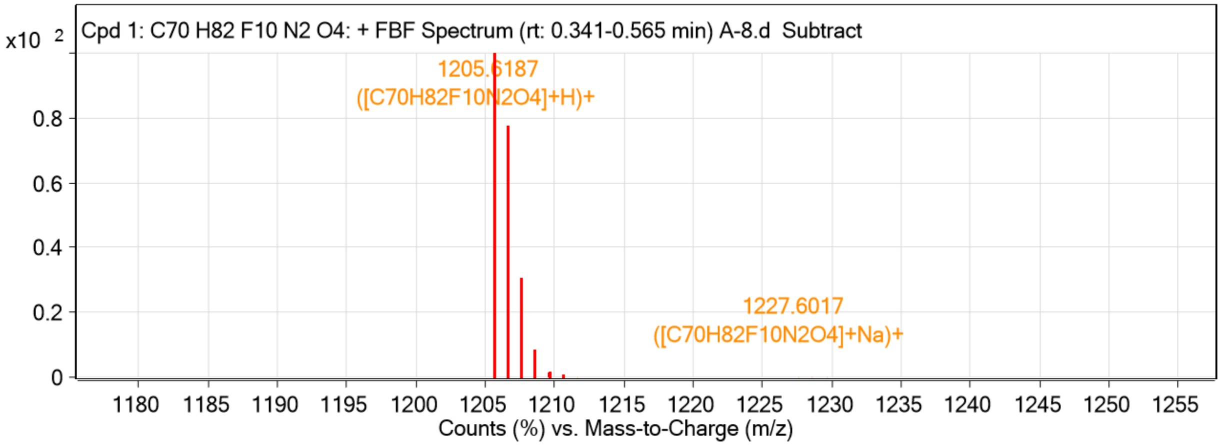

MS of **A-8**

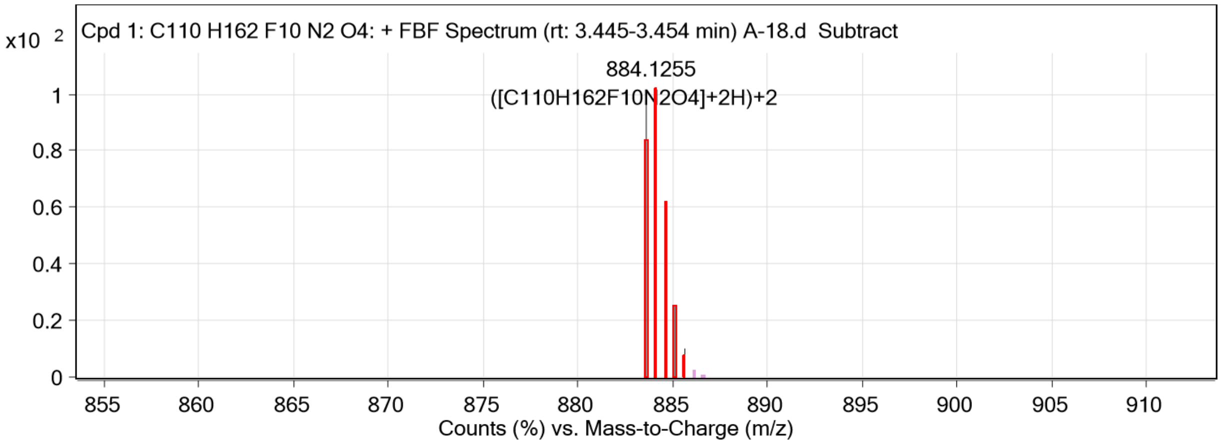

MS of **A-18**

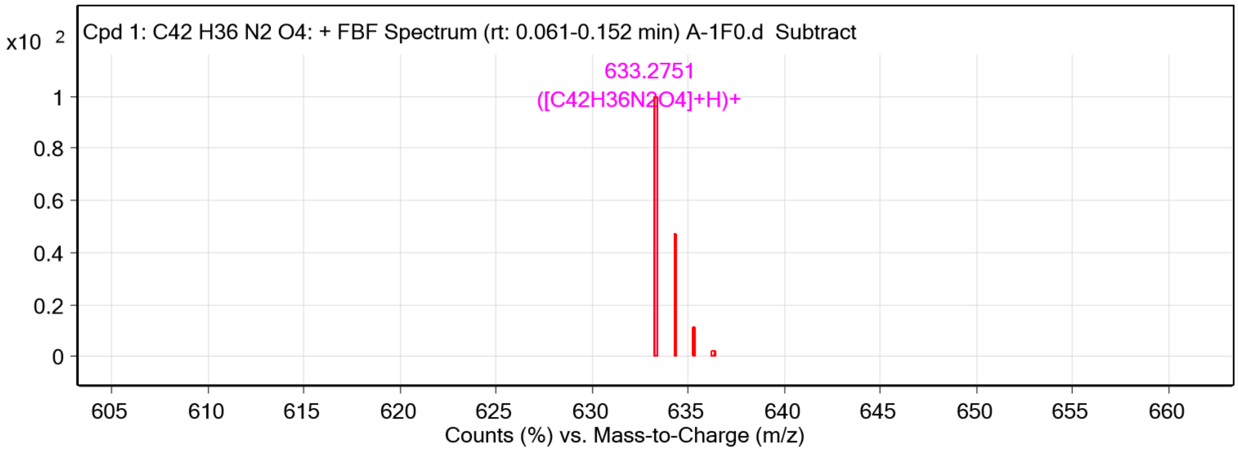

MS of **A-1F0**

5. Photophysical Data in Solution

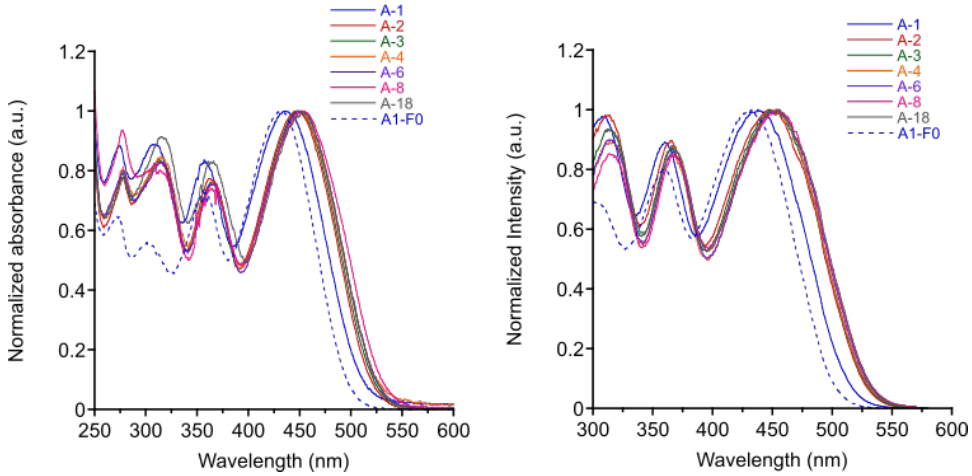

*Figure S1. Absorbance (left) and excitation spectra (right) of **A-R** compounds in chloroform solution.*

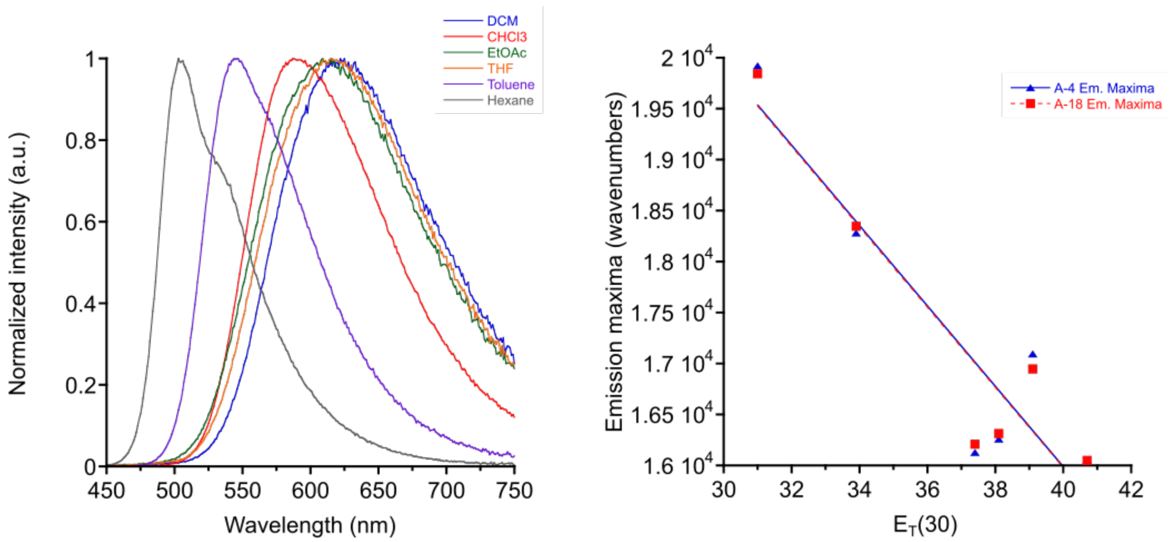

Figure S2. Emission spectra for A-18 (left) in various solvents and plot of A-4 and A-18 emission maxima energy against $E_T(30)$ values.

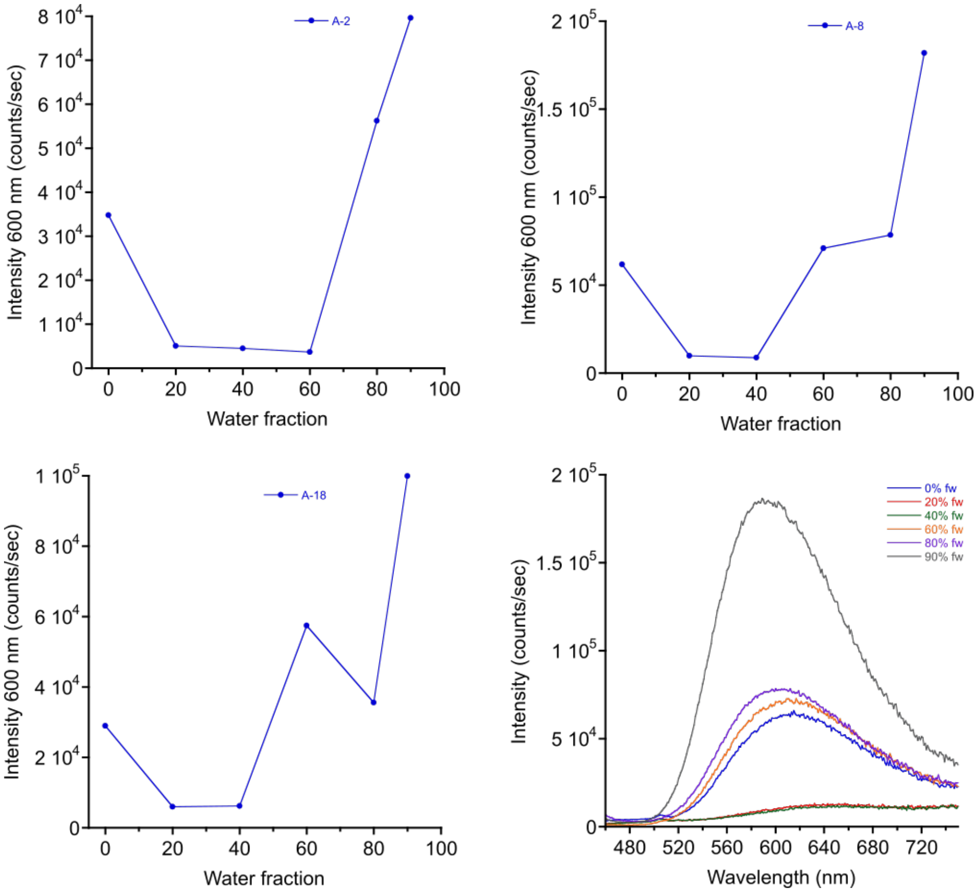

*Figure S3.Plots of fluorescence intensity at λem = 600 nm with increasing water fraction (fw) in THF/water mixtures for **A-2**, **A-8**, **A18**. (Lower right) Sample plot of emission spectra with increasing f_w for **A-8**. Spectra show modest hyspochromic shift with fluorescence turn-on.*

6. Additional crystal structure perspectives

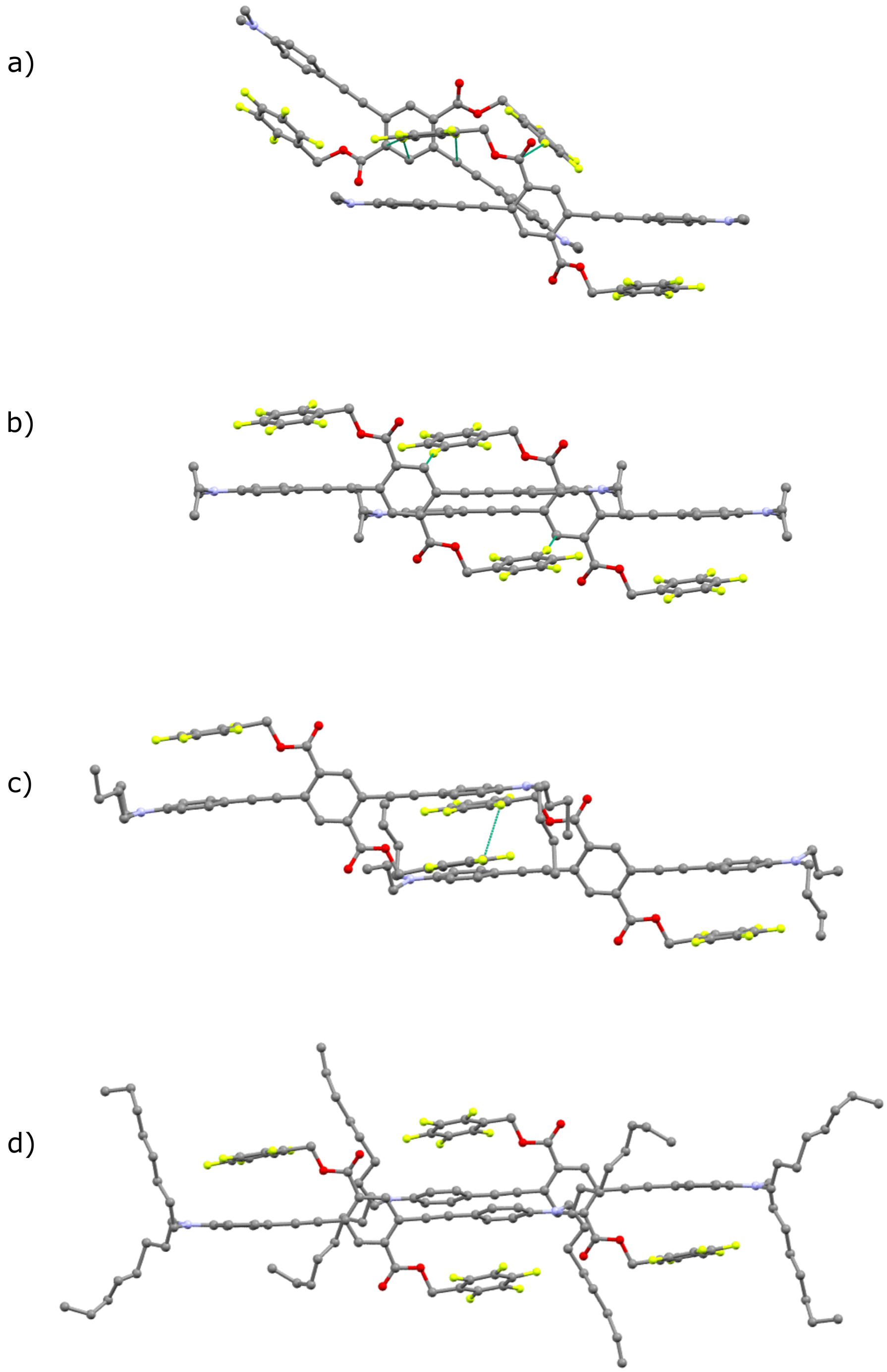

*Figure S4. Views of crystal structures along PE backbone. PE on left is in the front, while PE on the right side lies in the plane of neighboring molecules behind the left unit, showing intermolecular close contacts between neighboring stacks of PEs in a) **A-1** b) **A-2** c) **A-4** d) **A-8** Hydrogen atoms and disordered carbon atoms in alkyl chains (**A-4**, **A-8**) omitted for clarity.*

7. MC Response in Thin Films

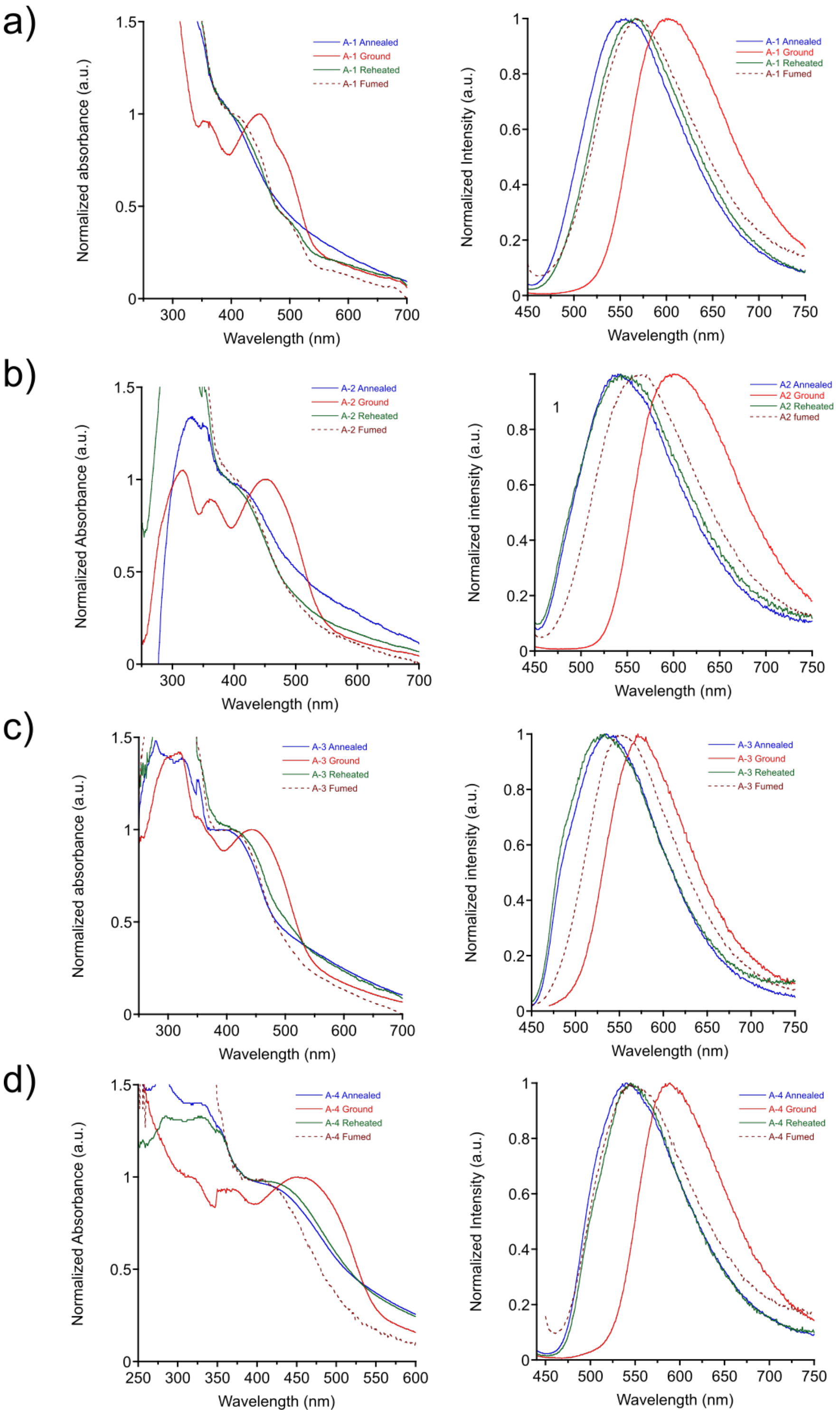

Figure S5. Absorbance (left) and emission (right) spectra of annealed, ground, reheated, and fumed samples of a) ***A-1****, b)* ***A-2****, c)* ***A-3****, d)* ***A-4.***

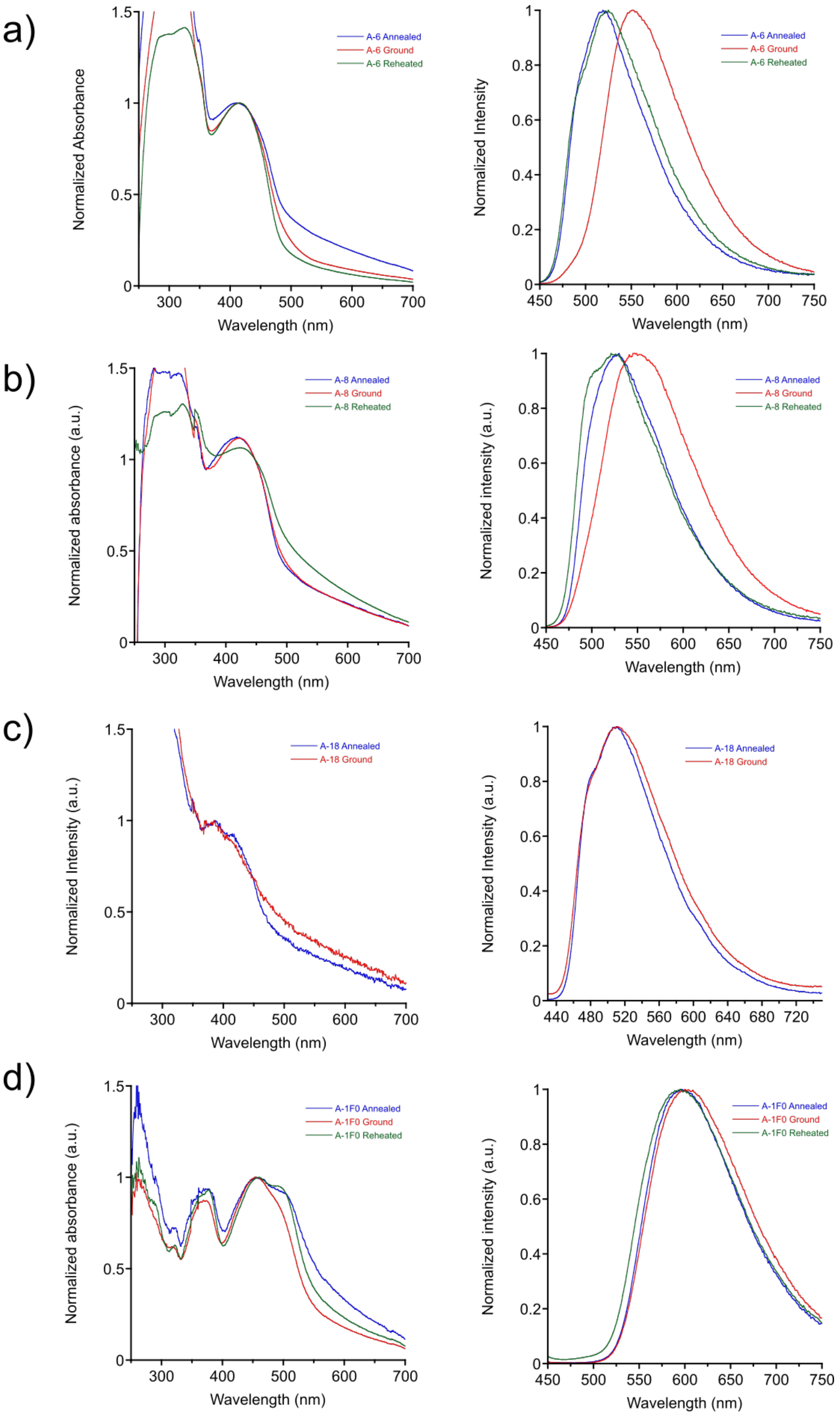

Figure S6. Absorbance (left) and emission (right) spectra of annealed, ground, reheated, and fumed samples of a) ***A-6****, b)* ***A-8****, c)* ***A-18****, d)* ***A-1F0.***

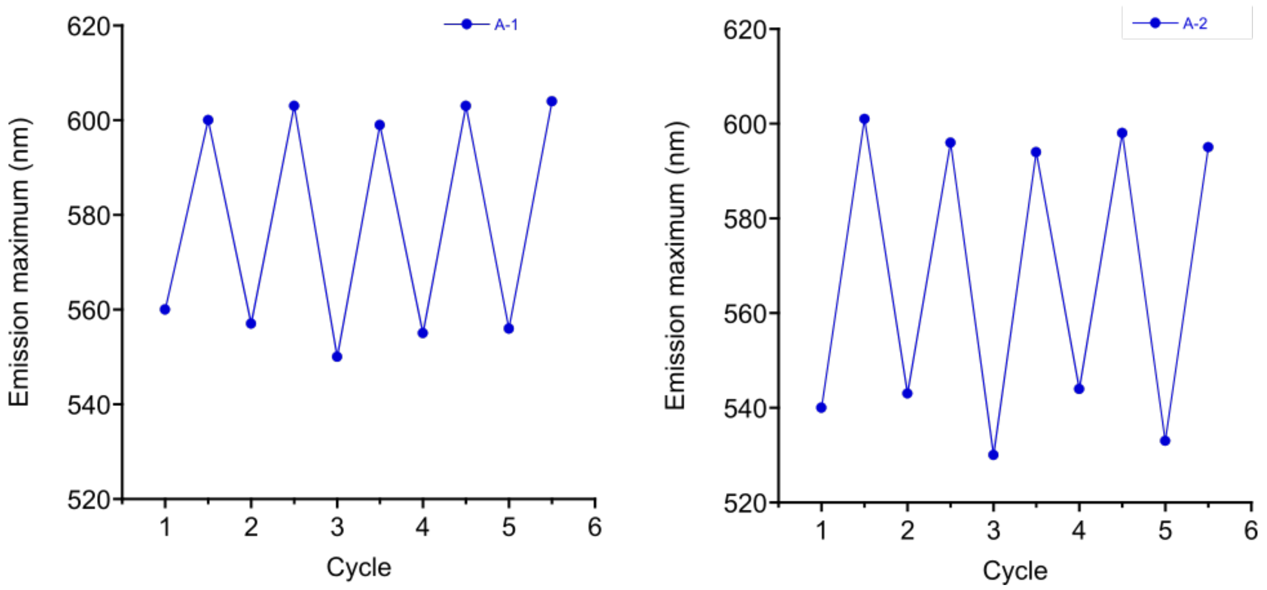

*Figure S7. Plots of emission maxima for **A-1** and **A-2** over 5 heat-grinding cycles heating at 220 °C.*

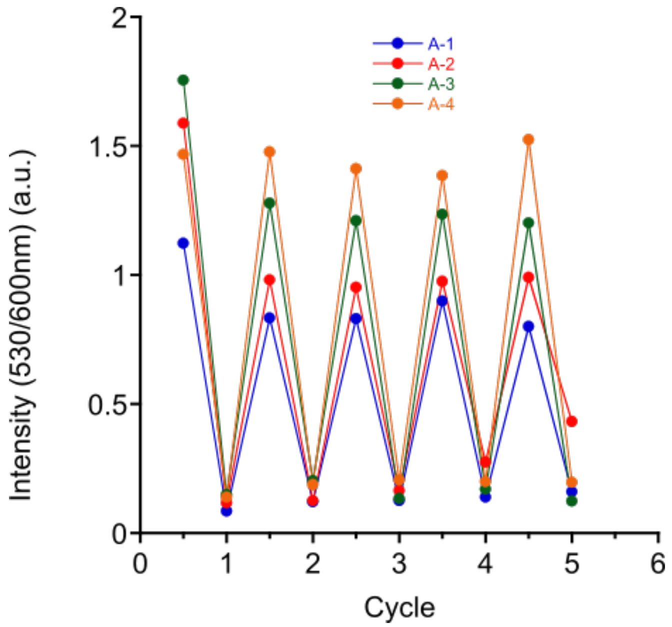

*Figure S8. Ratio of emission intensity measured at 530 nm / 600 nm for **A-1 - A-4** over five heat-grinding cycles, heating at 100 °C.*

8. DSC Data

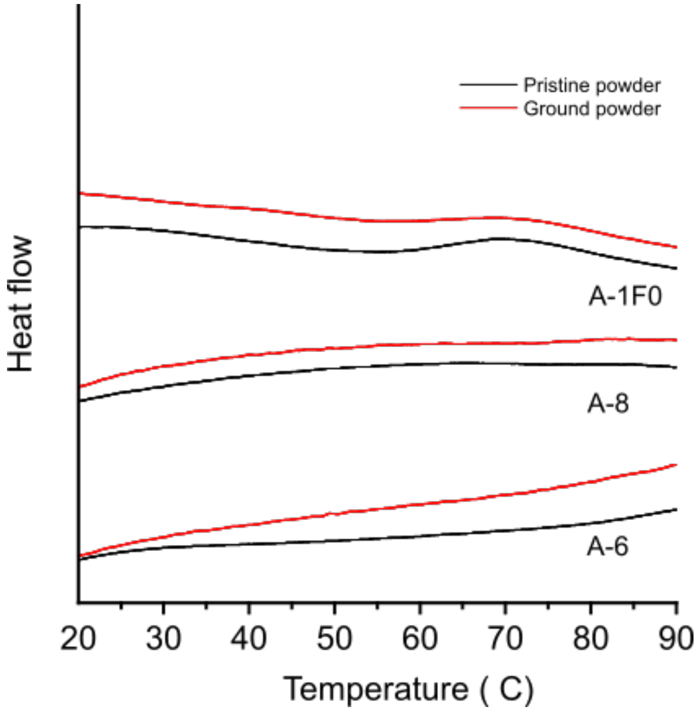

*Figure S9. DSC traces of pristine and ground powders for **A-6**, **A-8**, and **A-1F0**. First heats only.*

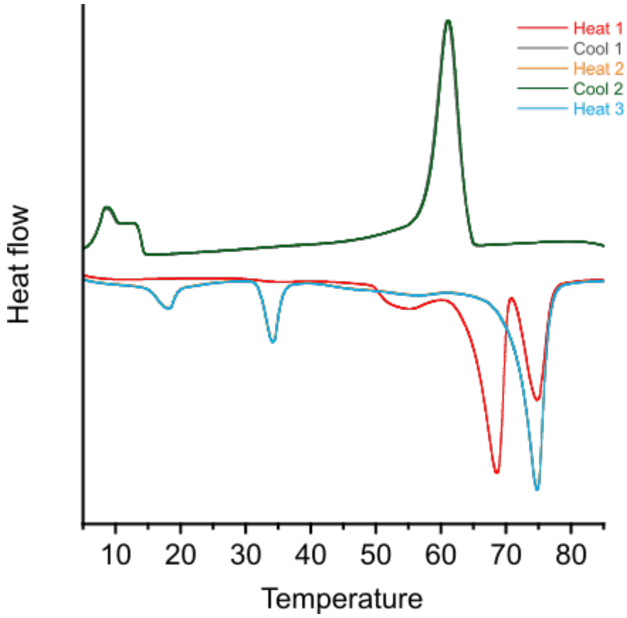

*Figure S10. **A-18** Pristine powder heated to melt. Compound shows polymorphism in first melt, then reversible endotherms in second and third heats at 18 and 34 °C.*

9. PXRD Data

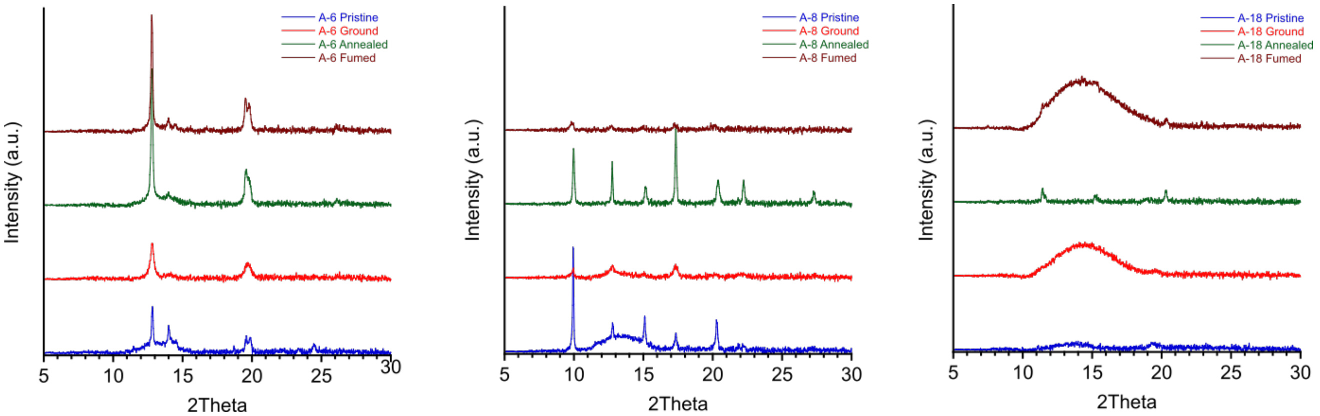

Figure S11. PXRD patterns of pristine, ground, annealed, and fumed films of ***A-6***, ***A-8***, *and* ***A-18.***

10. MC Response of A-4 in polymer hosts

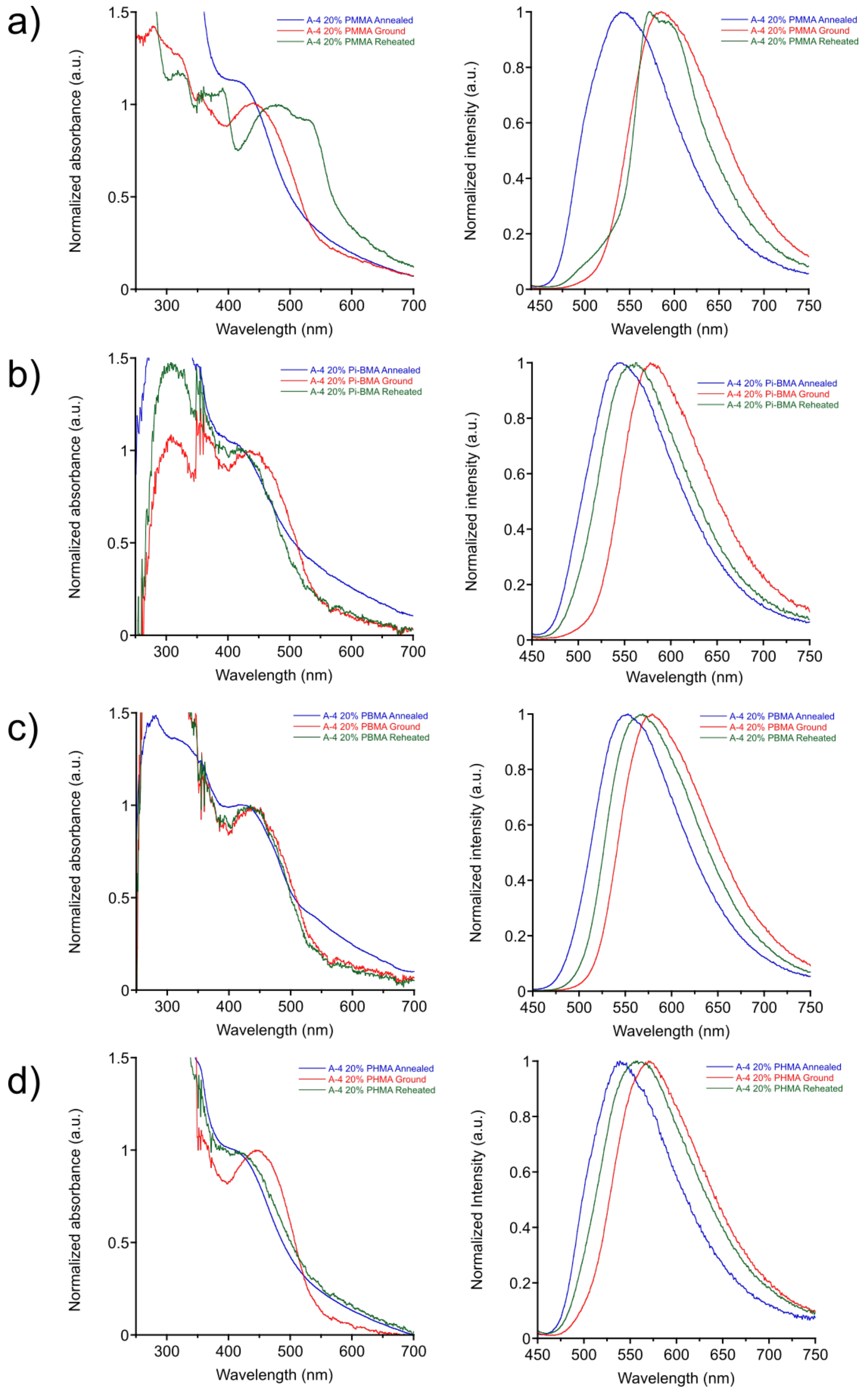

*Figure S12. Response of **A-4** in polymer hosts a) poly(methyl methacrylate) T_g = 99 °C, annealed 130 °C b) poly(isobutyl methacrylate) T_g = 55 °C, annealed 80 °C (no further recovery at higher temp) c) poly(butyl methacrylate) T_g = 20 °C, annealed 80 °C (decomposition at higher temp) and d) poly(hexyl methacrylate) T_g = -5 °C, annealed 100 °C.*

11. Crystallographic Tables

A-2

Table 1. Crystal data and structure refinement for NEt2-F5.

Identification code	SS170131_0m_a	
Empirical formula	C46 H34 F10 N2 O4	
Formula weight	868.75	
Temperature	100(2) K	
Wavelength	0.71073 Å	
Crystal system	Triclinic	
Space group	P-1	
Unit cell dimensions	a = 7.6139(6) Å	α= 95.814(2)°.
	b = 10.1143(8) Å	β= 92.348(2)°.
	c = 12.8470(10) Å	γ = 93.463(2)°.
Volume	981.35(13) $Å^3$	
Z	1	
Density (calculated)	1.470 Mg/m^3	
Absorption coefficient	0.126 mm^{-1}	
F(000)	446	
Crystal size	1.000 x 0.500 x 0.500 mm^3	
Theta range for data collection	3.056 to 37.394°.	
Index ranges	-12<=h<=12, -17<=k<=17, -21<=l<=21	
Reflections collected	31384	
Independent reflections	10179 [R(int) = 0.0273]	
Completeness to theta = 25.242°	99.6 %	
Absorption correction	Semi-empirical from equivalents	
Refinement method	Full-matrix least-squares on F^2	
Data / restraints / parameters	10179 / 0 / 282	
Goodness-of-fit on F^2	1.047	
Final R indices [I>2sigma(I)]	R1 = 0.0538, wR2 = 0.1443	
R indices (all data)	R1 = 0.0746, wR2 = 0.1567	
Extinction coefficient	n/a	
Largest diff. peak and hole	0.807 and -0.238 e.$Å^{-3}$	

A-4

Table 1. Crystal data and structure refinement for A-4_ss02_anis2.

Identification code	A-4_ss02_anis2	
Empirical formula	C54 H50 F10 N2 O4	
Formula weight	947.04	
Temperature	100(2) K	
Wavelength	1.54178 Å	
Crystal system	Triclinic	
Space group	P-1	
Unit cell dimensions	a = 12.3920(7) Å	α= 95.161(3)°.
	b = 17.6527(9) Å	β= 103.759(3)°.
	c = 18.8864(10) Å	γ = 106.881(3)°.
Volume	3783.4(4) $Å^3$	
Z	4	
Density (calculated)	1.663 Mg/m^3	
Absorption coefficient	0.975 mm^{-1}	
F(000)	1984	
Crystal size	0.336 x 0.169 x 0.104 mm^3	
Theta range for data collection	2.445 to 62.385°.	
Index ranges	-13<=h<=14, -20<=k<=20, -21<=l<=20	
Reflections collected	64095	
Independent reflections	11878 [R(int) = 0.0630]	
Completeness to theta = 62.385°	98.7 %	
Refinement method	Full-matrix least-squares on F^2	
Data / restraints / parameters	11878 / 61 / 901	
Goodness-of-fit on F^2	3.541	
Final R indices [I>2sigma(I)]	R1 = 0.2346, wR2 = 0.5424	
R indices (all data)	R1 = 0.3115, wR2 = 0.5936	
Extinction coefficient	n/a	
Largest diff. peak and hole	2.446 and -0.601 e.$Å^{-3}$	

A-8

Table 1. Crystal data and structure refinement for A-8_anis_5.

Identification code	A-8_anis_5	
Empirical formula	C70 H82 F10 N2 O4	
Formula weight	1205.37	
Temperature	100(2) K	
Wavelength	1.54184 Å	
Crystal system	Monoclinic	
Space group	$P2_1/c$	
Unit cell dimensions	a = 22.741 Å	α= 90°.
	b = 7.648 Å	β= 96.81°.
	c = 35.273 Å	γ = 90°.
Volume	6091.7 $Å^3$	
Z	4	
Density (calculated)	1.314 Mg/m^3	
Absorption coefficient	0.846 mm^{-1}	
F(000)	2552	
Crystal size	0.320 x 0.085 x 0.030 mm^3	
Theta range for data collection	1.956 to 70.211°.	
Index ranges	-27<=h<=27, -9<=k<=9, -42<=l<=42	
Reflections collected	106366	
Independent reflections	11434 [R(int) = 0.0744]	
Completeness to theta = 67.684°	100.0 %	
Absorption correction	Semi-empirical from equivalents	
Max. and min. transmission	0.7533 and 0.6149	
Refinement method	Full-matrix least-squares on F^2	
Data / restraints / parameters	11434 / 308 / 979	
Goodness-of-fit on F^2	1.159	
Final R indices [I>2sigma(I)]	R1 = 0.0901, wR2 = 0.1722	
R indices (all data)	R1 = 0.1071, wR2 = 0.1794	
Extinction coefficient	n/a	
Largest diff. peak and hole	0.665 and -0.444 e.$Å^{-3}$	

A-1F0

Table 1. Crystal data and structure refinement for A-1F0.

Identification code	A-1F0_SS051016_0m_a	
Empirical formula	C42 H36 N2 O4	
Formula weight	632.73	
Temperature	103(2) K	
Wavelength	0.71073 Å	
Crystal system	Triclinic	
Space group	P-1	
Unit cell dimensions	a = 8.8034(5) Å	α= 102.1170(10)°.
	b = 9.6631(6) Å	β= 107.2990(10)°.
	c = 10.5046(6) Å	γ = 102.2830(10)°.
Volume	797.11(8) Å^3	
Z	1	
Density (calculated)	1.318 Mg/m^3	
Absorption coefficient	0.085 mm^{-1}	
F(000)	334	
Crystal size	1.000 x 0.500 x 0.500 mm^3	
Theta range for data collection	2.855 to 33.399°.	
Index ranges	-13<=h<=13, -14<=k<=14, -16<=l<=16	
Reflections collected	22040	
Independent reflections	6175 [R(int) = 0.0273]	
Completeness to theta = 25.242°	99.8 %	
Absorption correction	Semi-empirical from equivalents	
Refinement method	Full-matrix least-squares on F^2	
Data / restraints / parameters	6175 / 0 / 219	
Goodness-of-fit on F^2	1.033	
Final R indices [I>2sigma(I)]	R1 = 0.0466, wR2 = 0.1223	
R indices (all data)	R1 = 0.0609, wR2 = 0.1328	
Extinction coefficient	n/a	
Largest diff. peak and hole	0.596 and -0.196 e.Å^{-3}	

References

1 K. Suzuki, A. Kobayashi, S. Kaneko, K. Takehira, T. Yoshihara, H. Ishida, Y. Shiina, S. Oishi and S. Tobita, *Phys. Chem. Chem. Phys.*, 2009, **11**, 9850–9860.

2 G. A. Reynolds and K. H. Drexhage, *Opt. Commun.*, 1975, **13**, 222–225.

3 G. M. Sheldrick, *Acta Crystallogr. Sect. A*, 1990, **46**, 467–473.

4 G. M. Sheldrick, *Acta Crystallogr. Sect. A Found. Crystallogr.*, 2007, **64**, 112–122.

5 G. M. Sheldrick, No Title, http://shelx.uni-ac.gwdg.de/SHELX/index.php.

6 P. Müller, *Crystallogr. Rev.*, 2009, **15**, 57–83.

7 C. Aurisicchio, B. Ventura, D. Bonifazi and A. Barbieri, *J. Phys. Chem. C*, 2009, **113**, 17927–17935.

8 K. D. Theriault and T. C. Sutherland, *Phys. Chem. Chem. Phys.*, 2014, **16**, 12266–12274.

9 M. G. Vivas, D. L. Silva, L. De Boni, Y. Bretonniere, C. Andraud, F. Laibe-Darbour, J.-C. Mulatier, R. Zaleśny, W. Bartkowiak, S. Canuto and C. R. Mendonca, *J. Phys. Chem. B*, 2012, **116**, 14677–14688.

10 S. A. Sharber, R. N. Baral, F. Frausto, T. E. Haas, P. Müller and S. W. Thomas, *J. Am. Chem. Soc.*, 2017, **139**, 5164–5174.

11 E. Kolvari, A. Amoozadeh, N. Koukabi, S. Otokesh and M. Isari, *Tetrahedron Lett.*, 2014, **55**, 3648–3651.

12 C. Dengiz, O. Dumele, S. I. Kato, M. Zalibera, P. Cias, W. B. Schweizer, C. Boudon, J. P. Gisselbrecht, G. Gescheidt and F. Diederich, *Chem. - A Eur. J.*, 2014, **20**, 1279–1286.

13 L. Chen, Y. He, F. Liu, J. Liu, H. Zhang, F. Huo, S. Bo, H. Xiao and Z. Zhen, *Mater. Lett.*, 2017, **196**, 230–233.

14 D. Lehnherr, M. Adam, A. H. Murray, R. Mcdonald, F. Hampel and R. R. Tykwinski, 2017, **314**, 303–314.

15 W. Huang and H. Chen, *Macromolecules*, 2013, **46**, 2032–2037.

16 S. Ast, H. Müller, R. Flehr, T. Klamroth, B. Walz and H.-J. Holdt, *Chem. Commun.*, 2011, **47**, 4685.

17 S. Rihn, P. Retailleau, A. De Nicola, G. Ulrich and R. Ziessel, *J. Org. Chem.*, 2012, **77**, 8851–8863.

18 C. Aurisicchio, B. Ventura, D. Bonifazi and A. Barbieri, *J. Phys. Chem. C*, 2009, **113**, 17927–17935.

19 R. H. Pawle, T. E. Haas, P. Müller and S. W. Thomas III, *Chem. Sci.*, 2014, **5**, 4184–4188.

20 R. H. Pawle, A. Agarwal, S. Malveira, Z. C. Smith and S. W. Thomas, *Macromolecules*, 2014, **47**, 2250–2256.
